# Identifying new solid forms of a labile pharmaceutical compound by additive assisted mechanochemistry

**DOI:** 10.1039/d6mr00025h

**Published:** 2026-05-19

**Authors:** Azhidhack Hadjipour, Nutifafa Yao Crown, Gayatri Gayatri, Oisín N. Kavanagh

**Affiliations:** a School of Pharmacy, Newcastle University Newcastle upon Tyne UK Oisin.Kavanagh@newcastle.ac.uk

## Abstract

Mechanochemical processing of labile active pharmaceutical ingredients (APIs) presents a fundamental trade-off: added solvent can accelerate solid form transformations yet may simultaneously promote chemical degradation. This study aims to explore this tension with an oxidation prone model pharmaceutical, cysteamine. Specifically, we examine the effects and interactions of antioxidant incorporation, reactant stoichiometry, ball to powder ratio (BPR), milling duration, and solvent assisted grinding on solid form screening success rates and yield. We find that the incorporation of ascorbic acid (ASC) as an antioxidant markedly improved resistance to oxidative degradation under a large set of milling conditions. Systematic variation of ASC molar ratios revealed an optimal composition at 1 : 1 : 2 (cysteamine : coformer : ASC) which reproducibly improved cysteamine recovery (90–110%, *n* = 3) and promoted the formation of a new crystalline phase, verified by PXRD. However, increasing the ASC ratio beyond 1 : 1 : 5 led to diminished product quality, indicating a threshold beyond which performance declines. These findings establish practical design insights for solid-form screening of chemically fragile pharmaceuticals.

## Abstract 2

Ascorbic acid-assisted mechanochemistry enables the discovery and stabilisation of new cysteamine solid forms.

## Graphical abstract

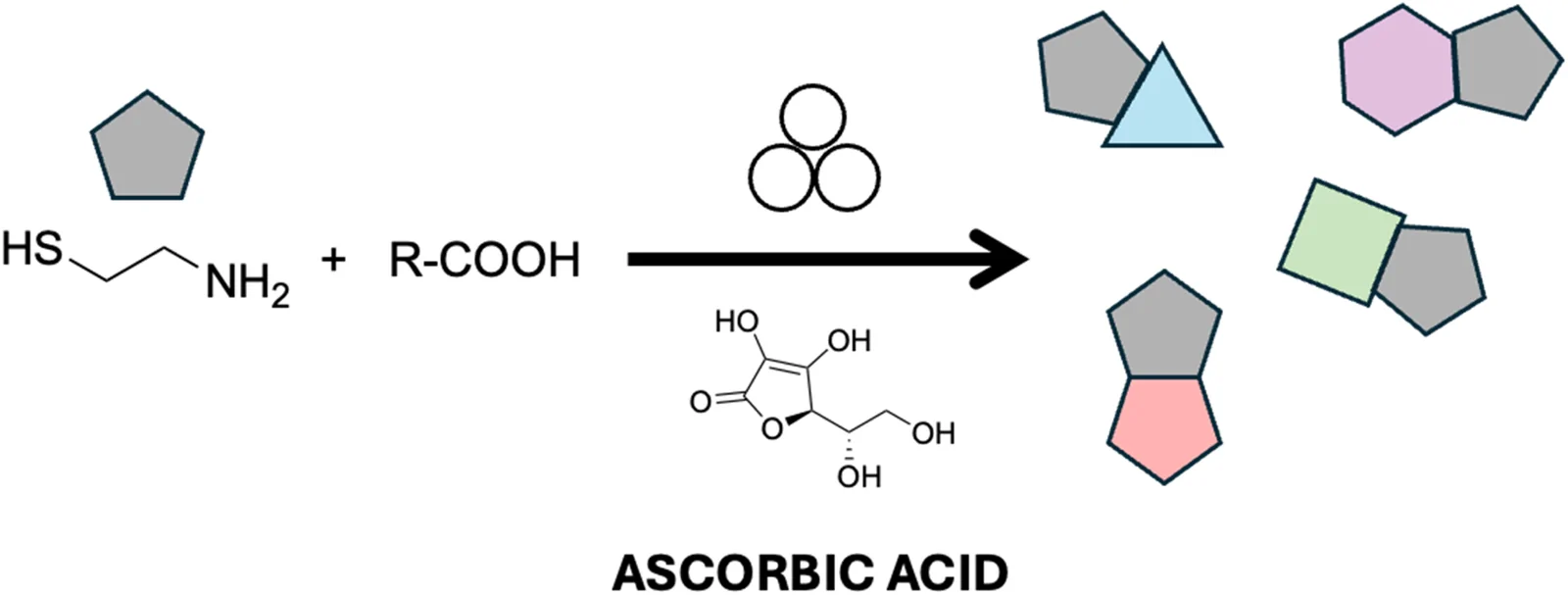

## 1. Introduction

Mechanochemistry has emerged as a powerful and sustainable approach to solid-state synthesis,^1^ and when applied to labile active pharmaceutical ingredients prone to oxidation or hydrolysis, can enable the discovery of new pharmaceutically acceptable forms which might be challenging to achieve through traditional solution based methods.^2^ This is sometimes referred to as crystal engineering. A key concept in the utilisation of crystal engineering approaches is that there is no change in the molecular structure of the medicine. This could enable drug companies to seek regulatory approval for their medicines using data from previous safety and efficacy investigations to bring a new product to market. In this way, crystal engineering can fine-tune the properties of pharmaceuticals with minimal regulatory and financial burden *i.e.*, through the FDA 505(b)(2) pathway and/or Article 10 of Directive 2001/83/EC.^3,4^

Cysteamine is a highly hygroscopic pharmaceutical which is susceptible to oxidative degradation,^5^ it is used to treat cystinosis, a rare disease. Despite good outcomes for patients who are treated with cysteamine, patient adherence is a limiting factor in the progression of the disease. Cysteamine adherence must be tightly controlled to supress cystine accumulation – particularly at night^6^ – yet perfect adherence drops to 50% in patients ≥11 years of age, with 44% of patients reporting that the unpleasant smell affected their motivation for adherence.^7^ Cystagon® (the immediate release formulation) requires patients to regimentally take their medicine every 6 hours, interrupting sleep and further contributing to non-adherence. PROCYSBI® was developed using enteric-coating technology to overcome these factors. However, a recent review highlights a large volume of literature reports describing dosage inconsistencies with enteric-coated formulations; in fact, dosage variability with PROCYSBI® is just as large as Cystagon®. In addition, the stability issues associated with this form mean that PROCYSBI® needs to be refrigerated and – once opened – has a shelf-life of 30 days, increasing cost. This, combined with the clinical factors detailed above served as a motivation for this study.

While we know that the addition of solvent to mechanochemical reactions can improve solid form screening success rates and can improve yields,^8^ this same solvent can accelerate decomposition, particularly for compounds that are labile.^9,10^ Indeed, oxidative mechanochemical reactions are known to proceed easily in the present of ambient moisture,^11^ several studies have reported the successful mechanochemical processing of sensitive organic compounds *e.g.*, peptides,^12^ nucleosides^13^ susceptible to hydrolysis, oxidation or thermal decomposition. In addition, the milling media can also act as a catalyst for these reactions *e.g.*, stainless steel, a common, cheap material used to construct milling jars and media.^14^ At other times, the coformer could accelerate the degradation of the active pharmaceutical ingredient and thus, the goal is to capture the ideal solid at some optimum set of conditions.^15^ Kinetic behaviour is strongly influenced by milling time, as overextended milling can lead to degradation pathways, this has been monitored comprehensively by *in situ*^16,17^ and *ex situ*^18^ analysis.

In this study we wanted to evaluate the possible mitigating effect of incorporating antioxidant additives, such as ascorbic acid, into the mechanochemical reaction mixture, and to explore the effects of systematic changes to reaction conditions in a solid form screen for labile pharmaceuticals.

## 2. Materials and methods

Refer to SI.

## 3. Results & discussion

To examine the effect of additives and mechanochemical conditions on the success rates of synthesis, we systematically increased the complexity of each series of experiments with a set of predefined coformers. Table 1 summarises the outcome of these experiments in a traffic light system for cysteamine. Ellman's assay was used to confirm the presence of cysteamine in the obtained phases. A successful hit was defined as a flowing powder form, consisting of a new crystalline phase confirmed *via* PXRD. In addition, these mixtures were required to reproducibly (*n* = 3) return an acceptable percentage yield of cysteamine (90–110%) in the final ball-milled mixture.

**Table 1:** The ball-milling outcome of cysteamine mixtures are presented in a traffic light system. Mixtures with reproducible (*n* = 3) percentage yields of 90–110% in 
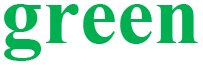
, not collectable samples (NC) in 
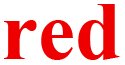
, and not in range (NIR), *i.e.*, yield <90% or >110%, and/or not reproducible (NR) or physical mixture (PM) in 
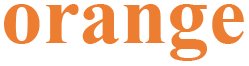
 colours

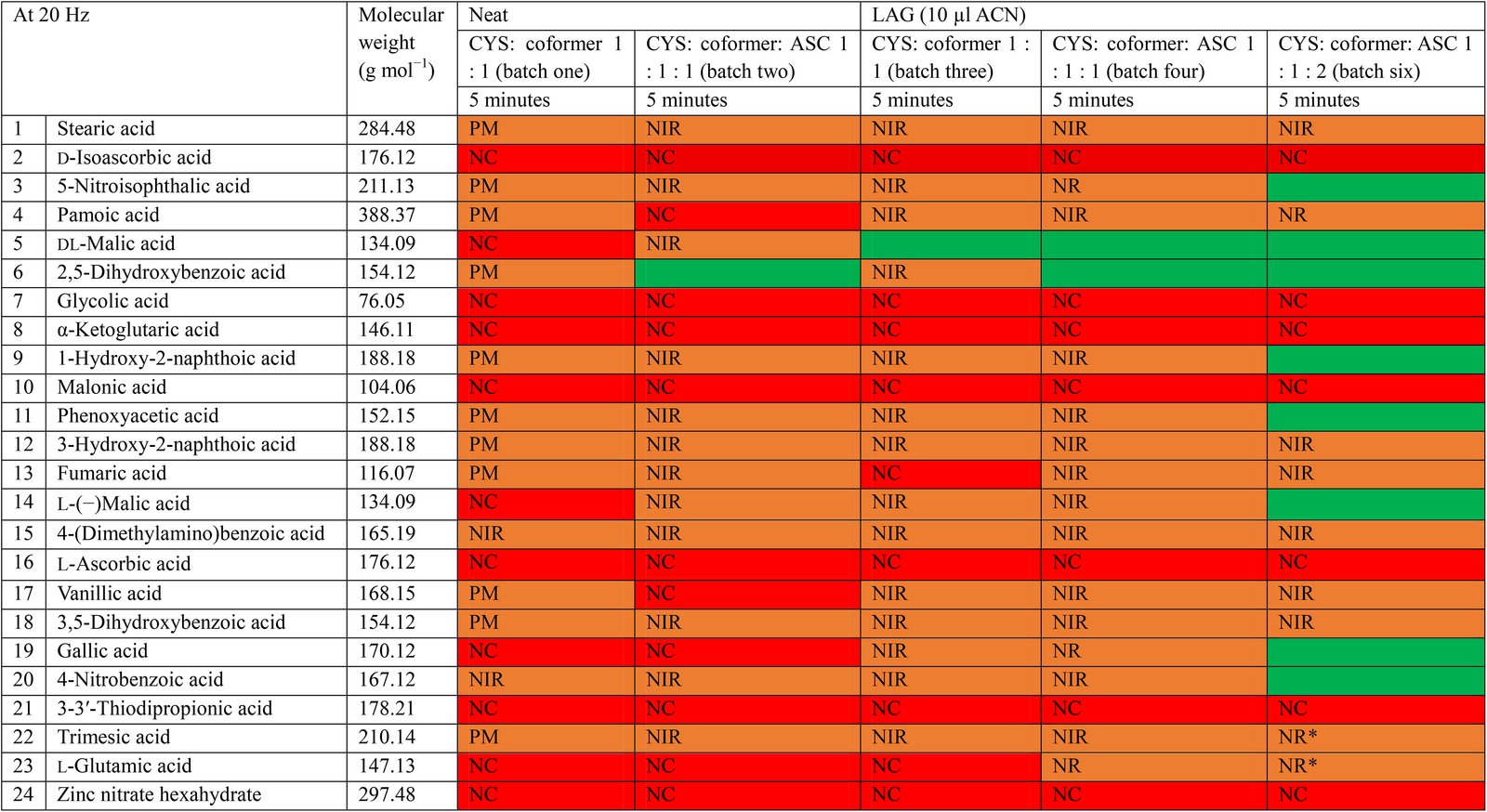

### 3.1. Results from screening: cysteamine

**Fig. 1:**
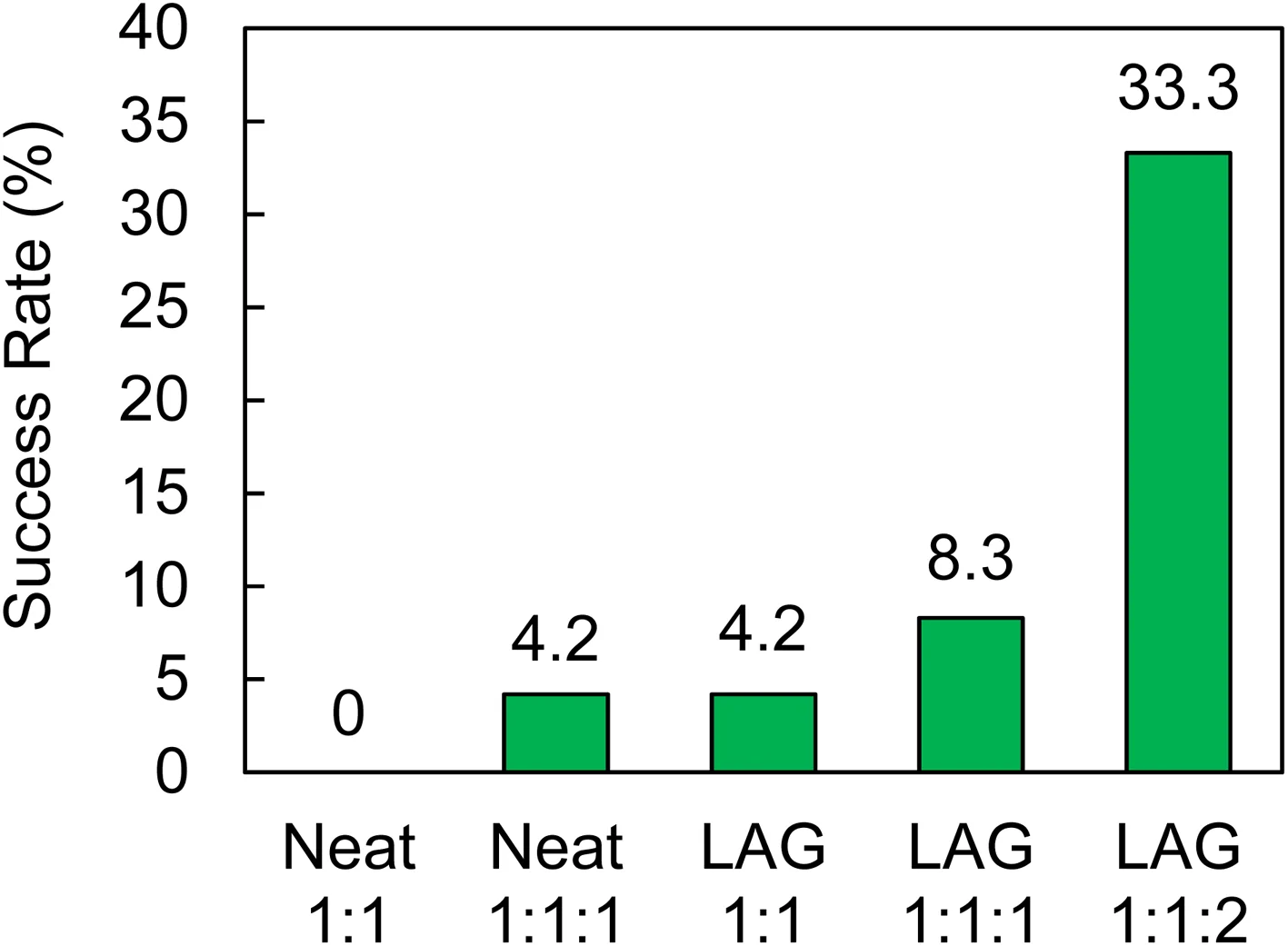
The screening success rates for various reaction conditions, where 1 : 1 refers to the molar equivalents of API: coformer and 1 : 1 : 1, API: coformer: ascorbic acid.

Initially, a neat 1 : 1 molar ratio mixture of cysteamine and each nominated coformer was ball milled at 20 Hz frequency and 5 minutes. The choice of lower range frequency and shorter milling time were selected due to the labile nature of cysteamine, as previous studies have shown that higher frequency and longer milling time can increase the temperature inside the mill which could cause product degradation.^19^ We realised that the addition of ascorbic acid and solvent, generally improved the chance of either obtaining a powder form from the neat experiment or improved the percentage yield in the final products (Fig. 1 and Table 1, S1–S4).

### 3.2. Addition of ascorbic acid to cysteamine containing mixtures

**Fig. 2:**
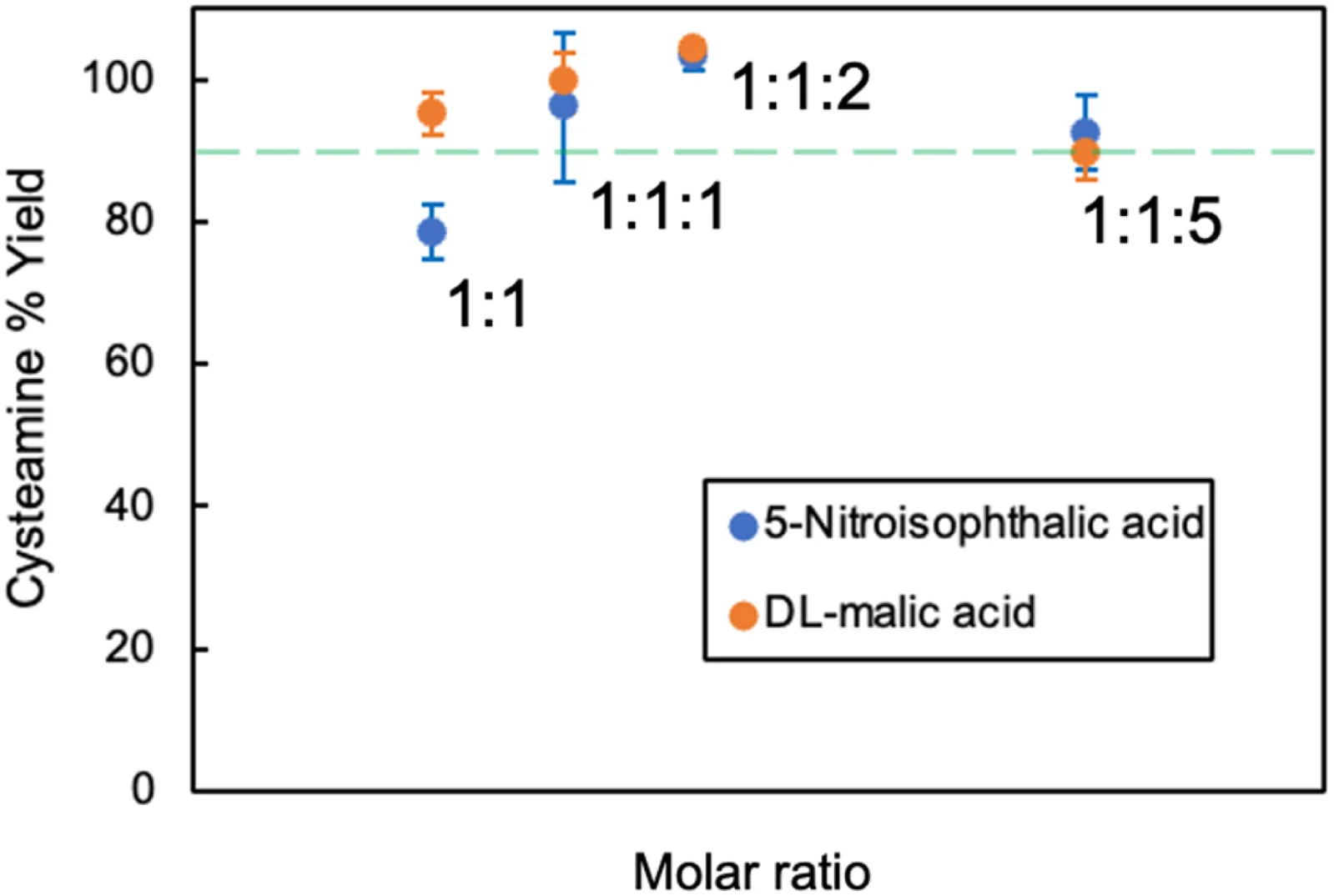
The influence of variable molar ratios of ascorbic acid to experimented mixtures *vs.* average percentage yield of cysteamine (*n* = 3); where 1 : 1 : 1 represents API: coformer: ascorbic acid. The milling frequency of 20 Hz, milling duration of 5 minutes, and LAG (10 µl ACN) are fixed for both mixtures through the experiment. The region of accepted range for cysteamine yield (90–110%) is marked in green dash line box. Standard error bars (standard deviation) reflect the reproducibility of the mixtures in relation to cysteamine percentage yield with 1 : 1 : 2 molar ratio demonstrating the optimum cysteamine percentage yield and reproducible results with minimum standard error.

The effect of ascorbic acid (ASC) on cysteamine containing mixtures was then systematically evaluated. Fig. 2 presents the results obtained for the 5-nitroisophthalic acid and dl-malic acid systems. As shown, incorporation of ASC up to a defined molar ratio (1 : 1 : 2 cysteamine: coformer: ascorbic acid) exerted a beneficial effect on the cysteamine percentage yield in the final mixtures. Beyond this ratio, this trend is reversed, indicating a detrimental impact associated with excessive ASC addition.

The improvement in cysteamine yield upon ASC addition may be attributed to its antioxidant properties, which can mitigate cysteamine degradation during milling. The observed decline in performance at elevated ASC concentrations could be rationalised by alterations in the ball to powder ratio (BPR). Specifically, increasing the ASC content could reduce the effective BPR, restricting the mobility of the milling ball. This reduction in available free volume could diminish the impact efficiency of the rotating ball, leading to decreased mechanical energy transfer to the mixture and consequently a reduced reaction rate between cysteamine and the acid coformer.

To ensure that the observed effects of ascorbic acid (ASC) addition on the milling outcome were not as a result of changes in the ball-to-powder ratio, an additional series of milling experiments was performed in which ASC was replaced with an inert component, NaCl, using an equivalent mass. As shown in Table 2, the incorporation of inert NaCl resulted either in samples that could not be collected or physical mixtures of the starting materials which revealed apparent decreased crystallinity, as evidenced by reduced peak sharpness in the corresponding PXRD patterns (Fig. S17–S19). This outcome demonstrates that the formation of the new crystalline phase and the improved cysteamine yields in the ASC containing mixtures (Fig. S1–S16 and Table S6) arise from the specific role of ascorbic acid rather than from changes in the ball to powder ratio (BPR), since the total mass and resulted BPR remained constant when ASC was replaced by NaCl.

**Table 2:** Investigating the influence of ascorbic acid addition *vs.* potential ball to powder ratio effects. Sodium chloride (NaCl) was used as an inert component for further investigation. Ascorbic acid was replaced with NaCl to achieve the same total mass of API: coformer: NaCl as previously prepared mixtures of 1 : 1 : 1, 1 : 1 : 2 & 1 : 1 : 5. The mixtures were ball-milled at frequency of 20 Hz for 5 minutes at the nominated LAG condition. Non collectable samples and simple physical mixtures of the starting materials are presented as NC and PM, respectively

Acid-coformer	Mixture code	Cysteamine (mg)	Acid (mg)	CYS : coformer : NaCl ratio	NaCL (mg)	ACN (µl)	Texture	Result (R1)	Result (R2)	Result (R3)
5-Nitroisophthalic acid	AvB-1	77	211	1 : 1: 1	176	10	Powder	PM	PM	PM
	AvB-2	77	211	1 : 1: 2	352	10	Powder	PM^a^	PM^a^	PM^a^
	AvB-3	77	211	1 : 1: 5	881	10	Powder	PM^a^	PM^a^	PM^a^
dl-Malic acid	AvB-4	77	134	1 : 1: 1	176	10	Gummy	NC	NC	NC
	AvB-5	77	134	1 : 1: 2	352	10	Powder	PM	PM	PM
	AvB-6	77	134	1 : 1: 5	881	10	Powder	PM	PM	PM
1-Hydroxy-2-naphthoic acid	AvB-7	77	188	1 : 1: 1	176	10	Powder	PM	PM	PM
	AvB-8	77	188	1 : 1: 2	352	10	Powder	PM^a^	PM^a^	PM^a^
	AvB-9	77	188	1 : 1: 5	881	10	Powder	PM^a^	PM^a^	PM^a^

a Decreased crystallinity.

A further noteworthy observation emerges when these results are compared with their counterparts lacking NaCl (Table S3, batch three). The decrease in BPR due to NaCl led to diminished performance relative to the corresponding batch three experiments. This reduction can be attributed to the increased filling degree inside the milling vessel for the NaCl containing mixtures and its consequent effects on both the BPR and the mechanical action of the milling media.

To investigate the influence of milling duration and its interplay with ASC on the final mixtures, a series of milling experiments were conducted for durations ranging from 1 to 30 minutes under LAG conditions with 1 : 1 : 1 molar ratio of cysteamine: coformer: ASC (Table 3). These were compared with 5 minute millings performed at 1 : 1 : 0 and 1 : 1 : 2 molar ratios. As presented in the Table 3, S3, and S6, increasing the milling duration to 10 minutes while maintaining the ascorbic acid molar ratio at 1 : 1 : 1 yields outcomes for 1-hydroxy-2-naphthoic acid that are comparable in range and reproducibility to those obtained from the 1 : 1 : 2 mixture milled for 5 minutes. A similar trend is observed for dl-malic acid and 5-nitroisophthalic acid at 10 and 15 minutes. However, extending the milling duration to 30 minutes results in a decline in product quality, indicating a limitation associated with prolonged milling in the context of a labile product such as cysteamine.

**Table 3:** Presents the influence of milling duration on cysteamine % yield for different ball-milled mixtures at LAG condition (10 µl ACN) at 20 Hz. 
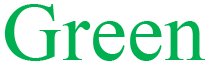
 represents the reproducibility (*n* = 3) of the results that are in the acceptable range of 90–110%. 
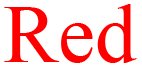
 represents not collectable mixture, and 
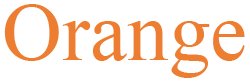
 represents not in range (NIR) or not reproducible (NR) result

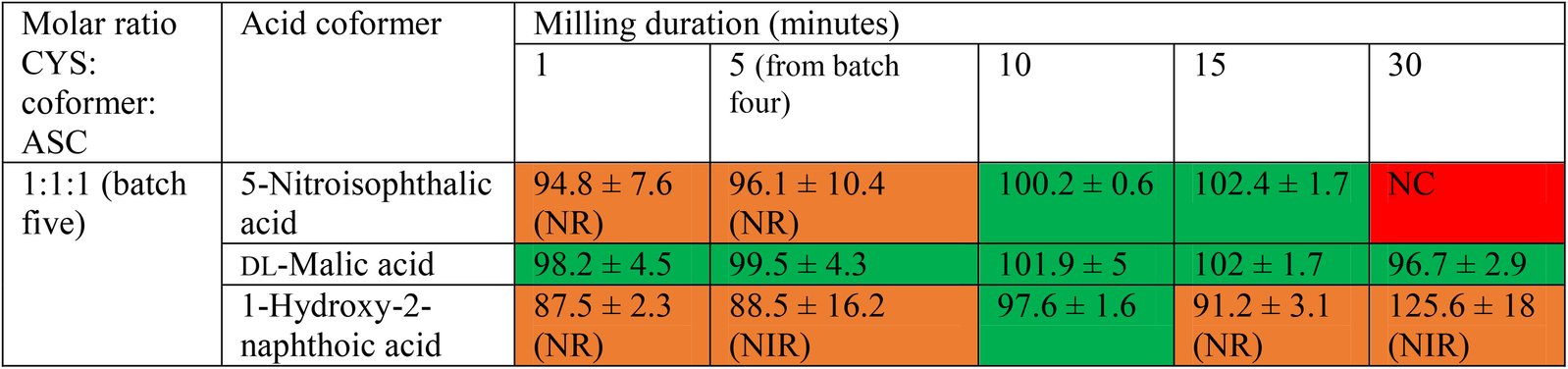

Further comparison of the results obtained at 5 minutes for the 1 : 1 : 0 and 1 : 1 : 2 molar ratios demonstrate the clear advantage of incorporating ascorbic acid into the mixture. Overall, these findings indicate that although the addition of ascorbic acid substantially enhances the outcome, careful control of milling duration is also essential. Ascorbic acid appears to protect cysteamine from degradation, provided that it is used within an appropriate molar range, thereby avoiding the negative effects associated with reduced BPR.

### 3.3. Synthesis of cysteamine bitartrate (CYS428)

Targeted synthesis of cysteamine bitartrate was undertaken because it is the active pharmaceutical ingredient used in both Cystagon® and PROCYSBI®. As a well-established salt form comprising cysteamine and tartaric acid, it serves as an appropriate benchmark for evaluating the newly generated crystal forms. Preparing this material in-house therefore enables direct assessment of intrinsic physicochemical properties of these crystal forms relative to the clinically used standard.

An initial attempt to prepare cysteamine bitartrate (CYS248) was carried out using a slurry/solution-based method adapted from US2018/0193292A1. A mixture of l-(+)-tartaric acid (6.5 mmol, 1 eq.) and l-ascorbic acid (0.13 mmol, 1/50 eq.) in 5 mL ethanol was heated to 60 °C, followed by the addition of cysteamine (6.5 mmol, 1 eq.) dissolved in 5 mL ethanol. The reaction mixture was stirred at 70–75 °C for 45 minutes and gradually cooled to 0 °C. The resulting solid was filtered, washed with cold ethanol and dried. The material obtained was a sticky, consolidated mass instead of a free-flowing crystalline product. Consequently, the slurry route was not pursued further, and attention was redirected towards mechanochemical preparation. Mechanochemical screening was next undertaken to identify suitable milling conditions for the preparation of cysteamine bitartrate (CYS248), the results are summarised below in Table 4.

**Table 4:** Product outcomes for cysteamine bitartrate preparation screening. Conditions varied include molar ratios of cysteamine, l-(+)-tartaric acid and ASC, solvent, frequency and milling time^a^

Run	Cysteamine (eq.)	l-(+)-tartaric acid (eq.)	ASC (eq.)	Solvent (10 µL)	Frequency (Hz)	Time (mins)	Result
R1	1	1	2	ACN	20	5	X
R2	1	1	1	ACN	20	5	X
R3	1	2	2	ACN	20	5	X
R4	1	2	1	ACN	20	5	X
R5	1	2	2	ACN	30	15	X
R6	1	2	—	ACN	20	5	X
R7	1	2	2	MeOH	20	10	X
R8	1	1	2	MeOH	20	10	X
R9	1	1	1	MeOH	20	10	X
R10	1	1	—	—	20	10	Crystal form L1
R11	1	1	1	—	20	10	X
R12	1	1	—	—	20	30	X
R13	1	1	—	MeOH	20	30	Crystal forms L1 and L2
R14	1	1	—	—	20	25	X

a *X* = Either a sticky solid or a physical mixture was obtained.

**Fig. 3:**
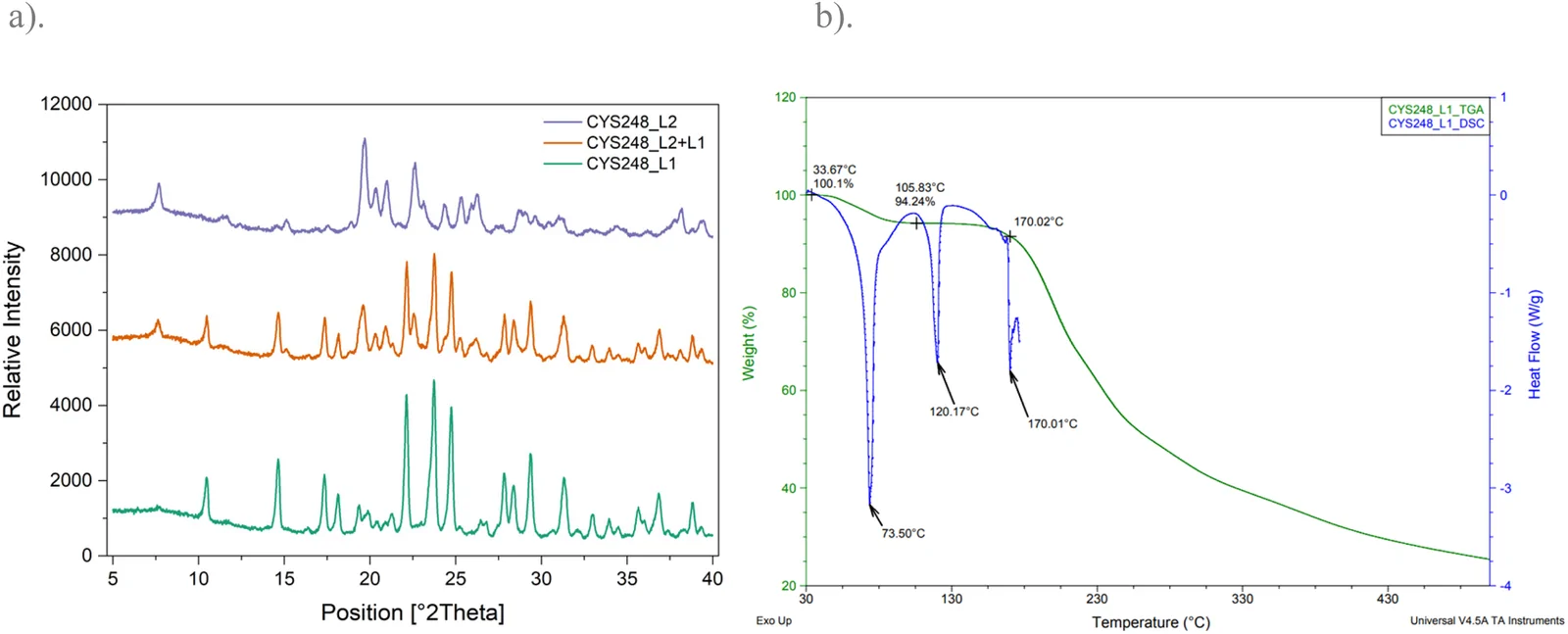
(a). Comparison of the PXRD diffractograms of cysteamine bitartrate (CYS248) crystal forms: L2, a mixture of L1 and L2, and L1. (b). Combined DSC thermogram and TGA endotherm of CYS248 crystal form L1.

The solids obtained from run 10 and run 13 were analysed by PXRD and the resultant diffraction patterns were compared with those of crystal forms L1 and L2 reported in patent US2018/0193292A1. These comparisons indicated that run 10 produced form L1 exclusively, whereas run 13 yielded a mixture of forms L1 and L2. PXRD monitoring over time showed that form L2 transformed into form L1 under ambient conditions (Fig. 3a).

Further characterisation of the crystal form L1 of CYS248 was carried out by investigating its thermal behaviour *via* DSC and TGA (Fig. 3b). The TGA curve showed an initial mass loss of approximately 6% roughly between 34 and 106 °C, consistent with the release of one molar equivalent of water from a monohydrate. This event coincided with a characteristic endothermic peak at approximately 74 °C, indicative of dehydration, which is consistent with the patent literature. Additionally, the DSC trace displayed a second, sharp endothermic peak at approximately 120 °C, occurring after the initial loss of water, which is likely an indication of the melting point of the anhydrous form. Following this, a broader high-temperature endotherm accompanied by significant mass loss was observed above 170 °C, corresponding to thermal decomposition.

These results confirmed that crystal form L1 of CYS248 corresponds to the thermodynamically stable hydrated form of cysteamine bitartrate. This conclusion was supported by the fact that the cysteamine bitartrate structure deposited in the Cambridge Structural Database (REFCODE: BILKIS) is also a hydrate. Consequently, the conditions used in run 10 were selected as the preferred mechanochemical method for preparing cysteamine bitartrate.

### 3.4. Confirming new crystal phase formation in successful hits

A series of additional experiments were performed to confirm the formation of a new crystal phase in our successful hits, and to understand the fate of the excess ASC used above 1 : 1: 1 molar ratio of the starting materials.

NaCl as an inert material was used to replace one of the three components at each synthesis, at exact mass of the replaced component, *i.e.*, NaCl: acid coformer: ASC molar ratio 1 : 1: 2 (A1), acid coformer: CYS: NaCl molar ratio 1 : 1: 2 (A2), and NaCl: CYS: ASC molar ratio 1 : 1: 2 (A3) were obtained applying the identical milling parameters to the original mixture of batch 6. In addition, cysteamine: ascorbic acid and ascorbic acid: acid coformer mixtures both at 1 : 1 molar ratios of the starting materials were prepared by neat milling at 5 minutes milling and 20 Hz. This provided a clear understanding of potential crystal formation for binary components without the impact of excess ASC.

Following PXRD analysis of the synthesised mixtures, the PXRD patterns of starting materials were analysed, *i.e.*, A1–A3, CYS: ASC, ASC: acid coformer *vs.* the final green mixtures of batch 6 (Fig. S22, S26, S29, S32, S35, S38, S41, and S44). Further comparison was made between A1–A3 *vs.* the final green mixtures of batch 6 (Fig. S23, S27, S30, S33, S36, S39, S42, and S45). A clear distinction between our identified hit and the aforementioned PXRD patterns is observed.

Differential Scanning Calorimetry (DSC) analysis of the aforementioned mixtures further confirmed a distinct endothermic peak of the final mixtures (Fig. S20, S25, S28, S31, S34, S37, S40, and S43) compared to the starting materials and A1–A3 thermograms. This further confirmed the presence of a distinct crystal phase.

Fourier Transform Infrared Spectroscopy (FTIR) also demonstrated a broader and deeper curve between 2500–3300 cm^−1^ indicating stronger/new hydrogen bonding compared to the starting materials and A1–A3 (Fig. S21). The presence of O–H sharp spikes in the final mixture in the region of 3300–3500 cm^−1^ along with no major change in the C=O region of about 1700 cm^−1^ further demonstrated the presence of ASC in the final mixture. Also, the lack of significant new peaks at 1550 cm^−1^ could indicate that the coformer (5-nitroisophthalic acid) is involved in hydrogen bonding rather than proton transfer. Furthermore, Fig. S24 presents a stack of FTIR spectrums for the successful hits in batch 6, indicating the presence of ASC, as before. This is in line with the PXRD patterns obtained for successful hits (Fig. S48) that indicate the presence of excess ASC along new crystal forms.

Complementary experiments were performed to further investigate the potential antioxidant effect of ascorbic acid on cysteamine degradation while ball milling in process. Cysteamine percentage yield was measured (Table S8), using Ellman's assay, for both cysteamine and a mixture of cysteamine–ascorbic acid (neat 1 : 1 molar ratio) at 5, 10, 15, and 30 minutes milling durations. A clear superiority of cysteamine degradation prevention in the mixtures containing ascorbic acid is observed (Fig. S46 and S47).

## 4. Conclusion

We found that the addition of ascorbic acid as an antioxidant to the cysteamine containing mixtures effectively suppresses the oxidation of cysteamine to enable the synthesis and improved yield of new cysteamine phases. Furthermore, ascorbic acid demonstrates a synergistic influence with LAG in improving the quality of the final mixtures. Our results highlight the utility of additive-enhanced mechanochemistry, when combined with controlled milling parameters, as an effective strategy for identifying new solid forms of labile pharmaceutical compounds. However, while the additive is a powerful tool for discovery, it is not a one-size-fits-all solution for every specific salt form.

## Author contributions

A. H. wrote the manuscript and led the experimental contributions. G .G. and N. Y. C. completed ball milling and PXRD experiments. O. K. was responsible for supervision, conceptualisation, and writing the manuscript.

## Conflicts of interest

There are no conflicts to declare.

## Supplementary Material

MR-003-D6MR00025H-s001

## Data Availability

All data is available in the supplemental information, we are happy to supply additional materials upon request. Supplementary information (SI) is available. See DOI: https://doi.org/10.1039/d6mr00025h.
