# Supplementary material for: Identifying new solid forms of a labile pharmaceutical compound by additive assisted mechanochemistry
Source: RSC Mechanochem. 2026 May 19;3(5):661–7. doi: 10.1039/d6mr00025h (PMC13551571; doi:10.1039/d6mr00025h)
Supplement: Supplementary file 1 [file MR-003-D6MR00025H-s001.pdf]

Supplementary Information (SI) for RSC Mechanochemistry.
This journal is © The Royal Society of Chemistry 2026

Supplementary information

# Identifying new solid forms of a labile pharmaceutical compound by additive assisted mechanochemistry

Azhidhack Hadjipour, Nutifafa Yao Crown, Gayatri Gayatri, Oisin N. kavanagh*

*School of Pharmacy, Newcastle University, Newcastle upon Tyne, UK.*

## Table of Contents


**Materials and Methods……………………………………………………………………………………………3**

**Cysteamine containing batches ............................................................................6**

**Table S1. Batch one....................................................................................................6**

**Table S2. Batch two ...................................................................................................7**

**Table S3. Batch three .................................................................................................8**

**Table S4. Batch four...................................................................................................9**

**Table S5. Batch five...................................................................................................10**

**Table S6. Batch six ...................................................................................................11**

**Table S7. Batch seven ...............................................................................................12**

**Stacks of PXRD patterns for green cysteamine containing mixtures of batch 6. ...............13**

**Figure S1. .................................................................................................................13**

**Figure S2. .................................................................................................................14**

**Figure S3. .................................................................................................................15**

**Figure S4. .................................................................................................................16**

**Figure S5. .................................................................................................................17**

**Figure S6. .................................................................................................................18**

**Figure S7. .................................................................................................................19**

**Figure S8. .................................................................................................................20**

**Figure S9. .................................................................................................................21**

**Figure S10. ...............................................................................................................22**

**Figure S11. ...............................................................................................................23**

**Figure S12. ...............................................................................................................24**

**Figure S13. ...............................................................................................................25**

**Figure S14. ...............................................................................................................26**

**Figure S15. ...............................................................................................................27**

**Figure S16. ...............................................................................................................28**

**Stacks of PXRD patterns investigating ascorbic acid addition vs ball to powder ratio........29**

**Figure S17. ...............................................................................................................29**

**Figure S18. ...............................................................................................................30**

**Figure S19. ...............................................................................................................31**

**FigureS20………………………………………………………………………………………………32**

**Figure S21………………………………………………………………………………………………33**

**Figure S22………………………………………………………………………………………………34**

## MATERIALS AND METHODS

Materials

Ellman's reagent was purchased from Sigma-Aldrich. HPLC grade acetonitrile and methanol were purchased from Scientific Laboratory Supplies, and all other materials were purchased from Tokyo Chemical Industries at the highest purity available.

Methods

*Ball milling*
All mixtures were synthesised using a ball mill apparatus (400MM, Retsch, Dusseldorf, Germany) equipped with two 10 ml stainless steel milling jars each with a grinding ball (stainless steel) of weight 13.5 g and diameter of about 15mm.

*Powder X –ray diffraction (PXRD)*
Powder X-ray diffraction was employed to characterize the solid-state forms obtained from the mechanochemical experiments. The diffraction patterns were recorded using PANalytical Empyrean Multi-Core diffractometer (Malvin Panalytical, UK) operating with Cu Kα radiation (1.5418Å) equipped with PIXcel 3D detector. The samples were gently grinded using an agate mortar and pestle to obtain a fine and homogeneous powder, which was then evenly spread onto a PXRD sample holder (disk) to minimize preferred orientation effects. Data were collected over the 2θ range of 5–40°, with a step size of 0.02° and a total acquisition time of approximately 5 minutes per sample with operating parameters set at 40kV and 40mA. These relatively rapid scans at ambient conditions were sufficient to identify phase purity against PXRD of the starting materials. Data was analysed with HighScore software to identify new phase crystallinity and further stacked using origin software for illustration purposes.

*Ellman's-Assay*
A 1.25 mM solution of 5, 5'-dithiobis-(2-nitrobenzoic acid) (DTNB) was prepared in a 50:50 methanol-water (MeOH: $H_2O$) mixture and stored in darkness. To each 200 µL experimental aliquot, 200 µL of the DTNB stock was added and adjusted to a final volume of 4 mL by the addition of 3.6 mL of PBS (0.1 M, pH 8.0), with pH of 8 being critical to ensure efficient derivatisation of thiol groups. The reaction mixtures were then incubated at ambient temperature for 15 minutes to allow complete reaction between the free thiol groups and DTNB. Following incubation, 3 mL of the derivatised solution was transferred into a quartz cuvette for spectrophotometric analysis. Care was taken to ensure that an excess of DTNB was maintained in all reactions to drive complete conversion and ensure accurate quantification. For UV-Vis, derivatisation of the free thiol produced a prominent absorbance peak at 412 nm, indicative of 5-thio-2-nitrobenzoic acid (TNB). For reference, DTNB had a $\lambda_{max}$ of 323 nm.

*Ellman's-Cysteine (free thiol) UV-vis calibration curve*
Cysteine standards of defined concentrations were prepared and analysed under identical experimental conditions using the Ellman's assay. Absorbance was measured at 412 nm, corresponding to the absorption maximum ($\lambda_{max}$) of 5-thio-2-nitrobenzoate (TNB) anion

generated upon reaction of 5,5'-dithiobis(2-nitrobenzoic acid) (DTNB) with free thiol groups. Measurements were performed in triplicate (n=3) for each standard. Thee mean absorbance values were plotted against cysteine concentration to construct a linear calibration curve, from which the corresponding regression equation was obtained as Y=13.729x-0.0001($R^2$ = 1).

*Monitoring the presence of Cysteamine in the final mixture*

The presence of free thiol in the collected samples was confirmed via Ellman's assay, utilising a UV-vis spectrophotometry. Additionally, the related free thiol concentrations and percentage yield of the cysteamine in the collected samples was calculated using UV-vis Ellman's-Cysteine (free thiol) calibration curve formula and the following formula, respectively:

Equation 1). % Yield of cysteamine in the collected sample =

$$\frac{(cysteamine\ calculated\ mass\ in\ the\ sample\ (mg)\ x\ 100)}{(cysteamine\ theoretical\ mass\ in\ the\ sample\ (mg))}$$

The above process was repeated in triplicates to ensure the reproducibility of the method and inclusion of the cysteamine percentage yields of the final mixtures, in the acceptable range of 90-110% according to ICH guidelines (ICH Q6A) and the labile nature of cysteamine.[17,18]

*Screen for cysteamine solids*

A 1:1 molar ratio of cysteamine (1 mmole, 70.62 mg) and nominated acid of choice (acid-coformer) were subjected to ball-milling with or without the addition of antioxidant (ascorbic acid). The milling process was performed both at neat (without solvent) and LAG (using acetonitrile as compatible solvent of choice) conditions. The process was performed in triplicates (n=3) to ensure the reproducibility of data.

*Batch ONE*

A 1:1 molar ratio of cysteamine: acid-coformer was milled at a fixed frequency of 20 Hz and duration of 5 minutes (neat grinding). The outcome of the mixing process in term of physical appearance and the calculated free thiol percentage yield is presented in table S1.

*Batch TWO*

Batch two was synthesised in a 1:1:1 molar ratio of Cysteamine: acid-coformer: ascorbic acid without the addition of any solvent (neat). The mixture was milled at a fixed frequency of 20 Hz and duration of 5 minutes. The outcome of the mixing process in term of physical appearance and the calculated free thiol percentage yield is presented in table S2.

*Batch THREE*

Batch three was synthesised in a 1:1 molar ratio of cysteamine: acid-coformer with the addition of 10µl acetonitrile as the solvent of choice (LAG). The mixture was milled at a fixed frequency of 20 Hz and duration of 5 minutes. The outcome of the mixing process in term of physical appearance and the calculated free thiol percentage yield is presented in table S3.

*Batch FOUR*

Batch four was synthesised in a 1:1:1 molar ratio of Cysteamine: acid-coformer: ascorbic acid with the addition of 10 µl acetonitrile as the solvent of choice (LAG). The mixture was milled at a fixed frequency of 20 Hz and duration of 5 minutes. The outcome of the mixing process in term of physical appearance and the calculated free thiol percentage yield is presented in table S4.

*Batch FIVE*

Batch five was synthesised in a 1:1:1 molar ratio of Cysteamine: acid-coformer: ascorbic acid with the addition of 10 µl acetonitrile as the solvent of choice (LAG). The mixture was milled at a frequency of 20 Hz and milling durations of 1, 10, 15 and 30 minutes separately, named as batch 5A, 5B, 5C & 5D, respectively. The outcome of the mixing processes in term of physical appearance and the calculated free thiol percentage yield is presented in table S5.

*Batch SIX*

Batch six was synthesised in a 1:1:2 molar ratio of Cysteamine: acid-coformer: ascorbic acid with the addition of 10 µl acetonitrile as the solvent of choice (LAG). The mixture was milled at a fixed frequency of 20 Hz and milling duration of 5 minutes. The outcome of the mixing process in term of physical appearance and the calculated free thiol percentage yield is presented in table S6.

*Batch Seven*

Batch seven was synthesised in a 1:1:5 molar ratio of Cysteamine: acid-coformer: ascorbic acid with the addition of 10 µl acetonitrile as the solvent of choice (LAG). The mixture was milled at a fixed frequency of 20 Hz and milling duration of 5 minutes. The outcome of the mixing process in term of physical appearance and the calculated free thiol percentage yield is presented in table S7.

*Investigating the influence of ascorbic acid addition vs potential ball to powder ratio effects*

In order to differentiate between the influences of adding ascorbic acid to the potential influence of resulting ball to powder ratio changes in the final mixtures, Sodium chloride (NaCl) was used as an inert component for further investigation (Table 2). Consequently, ascorbic acid was replaced with NaCl to achieve the same total mass of cysteamine: acid-coformer: NaCl as previously prepared mixtures of 1:1:1, 1:1:2 and 1:1:5 of cysteamine: acid-coformer: ascorbic acid, choosing DL-malic acid, 5-Nitroisophthalic acid, and 1-Hydroxy-2Naphthoic acid as acid-coformers of choice demonstrating variable outcome profiles in this regard (Figure 2 and Tables S4, S6 & S7). The potential formation of a new crystalline phase vs obtaining a physical mixture (Figure S2) was examined via PXRD.

**Cysteamine containing batches**

Table S1. Batch one synthesised by addition of 1:1 molar ratio of Cysteamine: acid-coformer at frequency of 20 Hz and milling duration of 5 minutes (neat grinding). The cysteamine in the final mixtures is presented as free thiol percentage yield for 3 repeats (n=3). PM stands for physical mixture.

| Mixture Code | Cysteamine (mg) | Acid-coformer | Acid (mg) | texture | Free thiol % Yield R1 | Free thiol % Yield R2 | Free thiol % Yield R3 | Mean ± SD (RSD%) |
|---|---|---|---|---|---|---|---|---|
| 1-1 | 77.1 ± 0.1 | Stearic acid | 284.4 ± 0.1 | Powder | PM | PM | PM | PM |
| 1-2 | 77.1 ± 0.1 | D-Isoascorbic acid | 176.1 ± 0.1 | Gummy | N/A | N/A | N/A | N/A |
| 1-3 | 77.1 ± 0.1 | 5-Nitroisophthalic acid | 211.1 ± 0.1 | Powder | PM | PM | PM | PM |
| 1-4 | 77.1 ± 0.1 | Pamoic acid | 388.3 ± 0.1 | Powder | PM | PM | PM | PM |
| 1-5 | 77.1 ± 0.1 | DL-malic acid | 134 ± 0.1 | Gummy | N/A | N/A | N/A | N/A |
| 1-6 | 77.1 ± 0.1 | 2,5-Dihydroxybenzoic acid | 154.1 ± 0.1 | Powder | PM | PM | PM | PM |
| 1-7 | 77.1 ± 0.1 | Glycolic acid | 76 ± 0.1 | Oily | N/A | N/A | N/A | N/A |
| 1-8 | 77.1 ± 0.1 | α-Ketoglutaric acid | 146 ± 0.1 | Sticky | N/A | N/A | N/A | N/A |
| 1-9 | 77.1 ± 0.1 | 1-Hydroxy-2-Naphthoic acid | 188.1 ± 0.1 | Powder | PM | PM | PM | PM |
| 1-10 | 77.1 ± 0.1 | Malonic acid | 104 ± 0.1 | Cluster | N/A | N/A | N/A | N/A |
| 1-11 | 77.1 ± 0.1 | Phenoxyacetic acid | 152.1 ± 0.1 | Powder | PM | PM | PM | PM |
| 1-12 | 77.1 ± 0.1 | 3-Hydroxy-2-naphthoic acid | 188.1 ± 0.1 | Powder | PM | PM | PM | PM |
| 1-13 | 77.1 ± 0.1 | Fumaric acid | 116 ± 0.1 | Powder | PM | PM | PM | PM |
| 1-14 | 77.1 ± 0.1 | L-(-)Malic acid | 134 ± 0.1 | Paste | N/A | N/A | N/A | N/A |
| 1-15 | 77.1 ± 0.1 | 4-(Dimethylamino)Benzoic acid | 165.1 ± 0.1 | Powder | 1.89 | 3.4 | 2.7 | 2.66 ± 0.62 (23.31) |
| 1-16 | 77.1 ± 0.1 | L-Ascorbic acid | 176.1 ± 0.1 | Sticky | N/A | N/A | N/A | N/A |
| 1-17 | 77.1 ± 0.1 | Vanillic acid | 168.1 ± 0.1 | Powder | PM | PM | PM | PM |
| 1-18 | 77.1 ± 0.1 | 3,5 Dihydroxybenzoic acid | 154.1 ± 0.1 | Powder | PM | PM | PM | PM |
| 1-19 | 77.1 ± 0.1 | Gallic acid | 170.1 ± 0.1 | Gummy | N/A | N/A | N/A | N/A |
| 1-20 | 77.1 ± 0.1 | 4-Nitrobenzoic acid | 167.1 ± 0.1 | Powder | 103.97 | 95.2 | 84.3 | 94.49 ± 8.05 (8.52) |
| 1-21 | 77.1 ± 0.1 | 3-3'-thiodipropionic acid | 178.2 ± 0.1 | Gummy | N/A | N/A | N/A | N/A |
| 1-22 | 77.1 ± 0.1 | Trimesic acid | 210.1 ± 0.1 | Powder | PM | PM | PM | PM |
| 1-23 | 77.1 ± 0.1 | L-Glutamic acid | 147.1 ± 0.1 | Sticky | N/A | N/A | N/A | N/A |
| 1-24 | 77.1 ± 0.1 | Zinc nitrate Hexahydrate | 297.4 ± 0.1 | Oily | N/A | N/A | N/A | N/A |

Table S2. Batch two synthesised in a 1:1:1 moral ratio of cysteamine: acid-coformer: ascorbic acid with no solvent addition (neat) at rotation frequency of 20 Hz and milling duration of 5 minutes. The cysteamine in the final mixtures is presented as free thiol percentage yield for 3 repeats (n=3).

| Mixture Code | Cysteamine (mg) | Acid-coformer | Acid (mg) | Ascorbic acid (mg) | Texture | Free thiol % Yield R1 | Free thiol % Yield R2 | Free thiol % Yield R3 | Mean ± SD (RSD%) |
|---|---|---|---|---|---|---|---|---|---|
| 2-1 | 77.1 ± 0.1 | Stearic acid | 284.4 ± 0.1 | 176.1 ± 0.1 | Powder | 47.6 | 51.6 | 48.2 | 49.13 ± 2.16 (4.39) |
| 2-2 | 77.1 ± 0.1 | D-Isoascorbic acid | 176.1 ± 0.1 | 0 | Sticky | N/A | N/A | N/A | N/A |
| 2-3 | 77.1 ± 0.1 | 5-Nitroisophthalic acid | 211.1 ± 0.1 | 176.1 ± 0.1 | Powder | 54.6 | 58 | 51.3 | 54.63 ± 3.35 (6.13) |
| 2-4 | 77.1 ± 0.1 | Pamoic acid | 388.3 ± 0.1 | 176.1 ± 0.1 | Paste | N/A | N/A | N/A | N/A |
| 2-5 | 77.1 ± 0.1 | DL-malic acid | 134 ± 0.1 | 176.1 ± 0.1 | Powder | 89.3 | 85.4 | 88.5 | 87.73 ± 2.06 (2.35) |
| 2-6 | 77.1 ± 0.1 | 2,5-Dihydroxybenzoic acid | 154.1 ± 0.1 | 176.1 ± 0.1 | Powder | 92.7 | 102.6 | 94 | 96.43 ± 5.38 (5.58) |
| 2-7 | 77.1 ± 0.1 | Glycolic acid | 76 ± 0.1 | 176.1 ± 0.1 | Oily | N/A | N/A | N/A | N/A |
| 2-8 | 77.1 ± 0.1 | α-Ketoglutaric acid | 146 ± 0.1 | 176.1 ± 0.1 | Sticky | N/A | N/A | N/A | N/A |
| 2-9 | 77.1 ± 0.1 | 1-Hydroxy-2-Naphthoic acid | 188.1 ± 0.1 | 176.1 ± 0.1 | Powder | 65.9 | 60.2 | 67.4 | 64.5 ± 3.80 (5.89) |
| 2-10 | 77.1 ± 0.1 | Malonic acid | 104 ± 0.1 | 176.1 ± 0.1 | Oily | N/A | N/A | N/A | N/A |
| 2-11 | 77.1 ± 0.1 | Phenoxyacetic acid | 152.1 ± 0.1 | 176.1 ± 0.1 | Powder | 80.5 | 85.2 | 78.6 | 81.43 ± 3.40 (4.17) |
| 2-12 | 77.1 ± 0.1 | 3-Hydroxy-2-naphthoic acid | 188.1 ± 0.1 | 176.1 ± 0.1 | Powder | 45.5 | 52.1 | 47.9 | 48.50 ± 3.34 (6.89) |
| 2-13 | 77.1 ± 0.1 | Fumaric acid | 116 ± 0.1 | 176.1 ± 0.1 | Powder | 37 | 45.3 | 39.2 | 40.50± 4.30 (10.62) |
| 2-14 | 77.1 ± 0.1 | L-(-)Malic acid | 134 ± 0.1 | 176.1 ± 0.1 | Powder | 59 | 65.2 | 56.5 | 60.23 ± 4.48 (7.44) |
| 2-15 | 77.1 ± 0.1 | 4-(Dimethylamino)Benzoic acid | 165.1 ± 0.1 | 176.1 ± 0.1 | Powder | 23.3 | 38.2 | 28.2 | 29.9 ± 7.59 (25.40) |
| 2-16 | 77.1 ± 0.1 | L-Ascorbic acid | 176.1 ± 0.1 | 0 | Oily | N/A | N/A | N/A | N/A |
| 2-17 | 77.1 ± 0.1 | Vanillic acid | 168.1 ± 0.1 | 176.1 ± 0.1 | Paste | N/A | N/A | N/A | N/A |
| 2-18 | 77.1 ± 0.1 | 3,5 Dihydroxybenzoic acid | 154.1 ± 0.1 | 176.1 ± 0.1 | Powder | 19.8 | 28.4 | 21.6 | 23.27± 4.54 (19.49) |
| 2-19 | 77.1 ± 0.1 | Gallic acid | 170.1 ± 0.1 | 176.1 ± 0.1 | Sticky | N/A | N/A | N/A | N/A |
| 2-20 | 77.1 ± 0.1 | 4-Nitrobenzoic acid | 167.1 ± 0.1 | 176.1 ± 0.1 | Powder | 55.7 | 60.5 | 54.4 | 56.87 ± 3.21 (5.65) |
| 2-21 | 77.1 ± 0.1 | 3-3'-thiodipropionic acid | 178.2 ± 0.1 | 176.1 ± 0.1 | Liquid | N/A | N/A | N/A | N/A |
| 2-22 | 77.1 ± 0.1 | Trimesic acid | 210.1 ± 0.1 | 176.1 ± 0.1 | Powder | 67.5 | 55.5 | 60 | 61 ± 6.06 (9.94) |
| 2-23 | 77.1 ± 0.1 | L-Glutamic acid | 147.1 ± 0.1 | 176.1 ± 0.1 | Paste | N/A | N/A | N/A | N/A |
| 2-24 | 77.1 ± 0.1 | Zinc nitrate Hexahydrate | 284.4 ± 0.1 | 176.1 ± 0.1 | Oily | N/A | N/A | N/A | N/A |

Table S3. Batch three synthesised by addition of 1:1 moral ratio of Cysteamine: acid-coformer with the addition of 10 µl acetonitrile as the solvent (LAG). The samples were subjected to 5 minutes milling at the frequency of 20 Hz.

| Mixture Code | Cysteamine (mg) | Acid-coformer | Acid (mg) | ACN (µl) | Texture | Free thiol % Yield R1 | Free thiol % Yield R2 | Free thiol % Yield R3 | Mean ± SD (RSD%) |
|---|---|---|---|---|---|---|---|---|---|
| 3-1 | 77.1 ± 0.1 | Stearic acid | 284.4 ± 0.1 | 10 | powder | 14.8 | 21.4 | 9.5 | 15.23 ± 5.96 (39.14) |
| 3-2 | 77.1 ± 0.1 | D-Isoascorbic acid | 176.1 ± 0.1 | 10 | Gummy | N/A | N/A | N/A | N/A |
| 3-3 | 77.1 ± 0.1 | 5-Nitroisophthalic acid | 211.1 ± 0.1 | 10 | powder | 83.5 | 78.2 | 73.8 | 78.5 ± 3.97 (5.06) |
| 3-4 | 77.1 ± 0.1 | Pamoic acid | 388.3 ± 0.1 | 10 | powder | 82.6 | 67.4 | 80.1 | 76.70 ± 8.15 (10.63) |
| 3-5 | 77.1 ± 0.1 | DL-malic acid | 134 ± 0.1 | 10 | powder | 93.7 | 99.2 | 92.2 | 95.03 ± 3.01 (3.88) |
| 3-6 | 77.1 ± 0.1 | 2,5-Dihydroxybenzoic acid | 154.1 ± 0.1 | 10 | powder | 73.6 | 81 | 79.2 | 77.93 ± 3.86 (4.95) |
| 3-7 | 77.1 ± 0.1 | Glycolic acid | 76 ± 0.1 | 10 | Oily | N/A | N/A | N/A | N/A |
| 3-8 | 77.1 ± 0.1 | α-Ketoglutaric acid | 146 ± 0.1 | 10 | Paste | N/A | N/A | N/A | N/A |
| 3-9 | 77.1 ± 0.1 | 1-Hydroxy-2-Naphthoic acid | 188.1 ± 0.1 | 10 | powder | 42.2 | 60.3 | 55 | 52.50 ± 9.31 (17.72) |
| 3-10 | 77.1 ± 0.1 | Malonic acid | 104 ± 0.1 | 10 | Oily | N/A | N/A | N/A | N/A |
| 3-11 | 77.1 ± 0.1 | Phenoxyacetic acid | 152.1 ± 0.1 | 10 | powder | 32.7 | 35 | 49.2 | 38.97 ± 8.94 (22.93) |
| 3-12 | 77.1 ± 0.1 | 3-Hydroxy-2-naphthoic acid | 188.1 ± 0.1 | 10 | powder | 85.3 | 89.1 | 83.7 | 86.03 ± 2.77 (3.22) |
| 3-13 | 77.1 ± 0.1 | Fumaric acid | 116 ± 0.1 | 10 | Soft creamy | N/A | N/A | N/A | N/A |
| 3-14 | 77.1 ± 0.1 | L-(-)Malic acid | 134 ± 0.1 | 10 | powder | 87.2 | 85.7 | 114.5 | 95.8 ± 16.21 (16.92) |
| 3-15 | 77.1 ± 0.1 | 4-(Dimethylamino)Benzoic acid | 165.1 ± 0.1 | 10 | powder | 3.8 | 12.3 | 5.7 | 7.27 ± 4.46 (61.39) |
| 3-16 | 77.1 ± 0.1 | L-Ascorbic acid | 176.1 ± 0.1 | 10 | Soft creamy | N/A | N/A | N/A | N/A |
| 3-17 | 77.1 ± 0.1 | Vanillic acid | 168.1 ± 0.1 | 10 | powder | 86.1 | 84.6 | 87.9 | 86.2 ± 1.65 (1.92) |
| 3-18 | 77.1 ± 0.1 | 3,5 Dihydroxybenzoic acid | 154.1 ± 0.1 | 10 | powder | 71.3 | 89.5 | 79 | 79.93 ± 9.14 (11.43) |
| 3-19 | 77.1 ± 0.1 | Gallic acid | 170.1 ± 0.1 | 10 | powder | 88.9 | 85.7 | 80.1 | 84.9 ± 4.45 (5.25) |
| 3-20 | 77.1 ± 0.1 | 4-Nitrobenzoic acid | 167.1 ± 0.1 | 10 | powder | 2.1 | 5.4 | 8.5 | 5.33 ± 3.20 (60.01) |
| 3-21 | 77.1 ± 0.1 | 3-3'-thiodipropionic acid | 178.2 ± 0.1 | 10 | Paste | N/A | N/A | N/A | N/A |
| 3-22 | 77.1 ± 0.1 | Trimesic acid | 210.1 ± 0.1 | 10 | powder | 87 | 70.4 | 82.8 | 80.07 ± 8.63 (10.78) |
| 3-23 | 77.1 ± 0.1 | L-Glutamic acid | 147.1 ± 0.1 | 10 | Sticky | N/A | N/A | N/A | N/A |
| 3-24 | 77.1 ± 0.1 | Zinc nitrate Hexahydrate | 284.4 ± 0.1 | 10 | Oily | N/A | N/A | N/A | N/A |

Table S4. Batch four synthesised in a 1:1:1 moral ratio of cysteamine: acid-coformer: ascorbic acid with the addition of 10 µl acetonitrile as the solvent (LAG) at 20 Hz frequency and milling duration of 5 minutes.

| Mixture Code | Cysteamine (mg) | Acid-coformer | Acid (mg) | Ascorbic acid (mg) | ACN (µl) | Texture | Free thiol % Yield R1 | Free thiol % Yield R2 | Free thiol % Yield R3 | Mean ± SD (RSD%) |
|---|---|---|---|---|---|---|---|---|---|---|
| 4-1 | 77.1 ± 0.1 | Stearic acid | 284.4 ± 0.1 | 176.1 ± 0.1 | 10 | Powder | 56.5 | 60 | 58.1 | 58.2 ± 1.75 (3.01) |
| 4-2 | 77.1 ± 0.1 | D-Isoascorbic acid | 176.1 ± 0.1 | 0 | 10 | Oily | N/A | N/A | N/A | N/A |
| 4-3 | 77.1 ± 0.1 | 5-Nitroisophthalic acid | 211.1 ± 0.1 | 176.1 ± 0.1 | 10 | Powder | 90 | 87.5 | 110.8 | 96.1 ± 10.44 (10.86) |
| 4-4 | 77.1 ± 0.1 | Pamoic acid | 388.3 ± 0.1 | 176.1 ± 0.1 | 10 | Powder | 25.6 | 30.7 | 21.4 | 25.9 ± 4.66 (17.98) |
| 4-5 | 77.1 ± 0.1 | DL-malic acid | 134 ± 0.1 | 176.1 ± 0.1 | 10 | Powder | 100.5 | 93.8 | 104.2 | 99.5 ± 4.30 (4.32) |
| 4-6 | 77.1 ± 0.1 | 2,5-Dihydroxybenzoic acid | 154.1 ± 0.1 | 176.1 ± 0.1 | 10 | Powder | 90.2 | 95.5 | 100.1 | 95.27 ± 4.95 (5.2) |
| 4-7 | 77.1 ± 0.1 | Glycolic acid | 76 ± 0.1 | 176.1 ± 0.1 | 10 | Sticky | N/A | N/A | N/A | N/A |
| 4-8 | 77.1 ± 0.1 | α-Ketoglutaric acid | 146 ± 0.1 | 176.1 ± 0.1 | 10 | Paste | N/A | N/A | N/A | N/A |
| 4-9 | 77.1 ± 0.1 | 1-Hydroxy-2-Naphthoic acid | 188.1 ± 0.1 | 176.1 ± 0.1 | 10 | Powder | 74.9 | 106.4 | 84.2 | 88.50 ± 16.18 (18.29) |
| 4-10 | 77.1 ± 0.1 | Malonic acid | 104 ± 0.1 | 176.1 ± 0.1 | 10 | Liquid | N/A | N/A | N/A | N/A |
| 4-11 | 77.1 ± 0.1 | Phenoxyacetic acid | 152.1 ± 0.1 | 176.1 ± 0.1 | 10 | Powder | 75.8 | 81.6 | 78.7 | 78.7 ± 2.9 (3.68) |
| 4-12 | 77.1 ± 0.1 | 3-Hydroxy-2-naphthoic acid | 188.1 ± 0.1 | 176.1 ± 0.1 | 10 | Powder | 72.3 | 65 | 74.1 | 70.47 ± 4.82 (6.84) |
| 4-13 | 77.1 ± 0.1 | Fumaric acid | 116 ± 0.1 | 176.1 ± 0.1 | 10 | Powder | 15.8 | 10.2 | 18.4 | 14.8 ± 4.19 (28.31) |
| 4-14 | 77.1 ± 0.1 | L-(-)Malic acid | 134 ± 0.1 | 176.1 ± 0.1 | 10 | Powder | 12.7 | 26.5 | 17.4 | 18.87 ± 7.02 (37.19) |
| 4-15 | 77.1 ± 0.1 | 4-(Dimethylamino)Benzoic acid | 165.1 ± 0.1 | 176.1 ± 0.1 | 10 | Powder | 56.4 | 71.8 | 58.9 | 62.37 ± 8.26 (13.25) |
| 4-16 | 77.1 ± 0.1 | L-Ascorbic acid | 176.1 ± 0.1 | 0 | 10 | Sticky | N/A | N/A | N/A | N/A |
| 4-17 | 77.1 ± 0.1 | Vanillic acid | 168.1 ± 0.1 | 176.1 ± 0.1 | 10 | Powder | 82.5 | 84 | 77.5 | 81.33 ± 3.4 (4.18) |
| 4-18 | 77.1 ± 0.1 | 3,5 Dihydroxybenzoic acid | 154.1 ± 0.1 | 176.1 ± 0.1 | 10 | Powder | 49.3 | 61.4 | 58.7 | 56.47 ± 6.35 (11.25) |
| 4-19 | 77.1 ± 0.1 | Gallic acid | 170.1 ± 0.1 | 176.1 ± 0.1 | 10 | Powder | 93.9 | 73 | 80.4 | 82.43 ± 10.6 (12.86) |
| 4-20 | 77.1 ± 0.1 | 4-Nitrobenzoic acid | 167.1 ± 0.1 | 176.1 ± 0.1 | 10 | Powder | 67.7 | 50.6 | 61.6 | 59.97 ± 8.67 (14.45) |
| 4-21 | 77.1 ± 0.1 | 3-3'-thiodipropionic acid | 178.2 ± 0.1 | 176.1 ± 0.1 | 10 | Liquid | N/A | N/A | N/A | N/A |
| 4-22 | 77.1 ± 0.1 | Trimesic acid | 210.1 ± 0.1 | 176.1 ± 0.1 | 10 | Powder | 85.3 | 86.2 | 80.5 | 84 ± 3.06 (3.65) |
| 4-23 | 77.1 ± 0.1 | L-Glutamic acid | 147.1 ± 0.1 | 176.1 ± 0.1 | 10 | Powder | 91.1 | 80.4 | 83.7 | 85.07 ± 5.48 (6.44) |
| 4-24 | 77.1 ± 0.1 | Zinc nitrate Hexahydrate | 284.4 ± 0.1 | 176.1 ± 0.1 | 10 | Liquid | N/A | N/A | N/A | N/A |

Table S5. Batch five synthesised in 1:1:1 moral ratio of cysteamine: acid-coformer: ascorbic acid with the addition of 10 µl acetonitrile as the solvent (LAG). The rotation frequency of 20 Hz and milling duration of 1, 10, 15 & 30 minutes were applied separately resulting in batch 5A, 5B, 5C & 5D, respectively.

| | Mixture Code | Cysteamine (mg) | Acid-coformer | Acid-coformer (mg) | ASC (mg) | ACN (µl) | Texture | Free thiol % Yield R1 | Free thiol % Yield R2 | Free thiol % Yield R3 | Mean ± SD (RSD%) |
|---|---|---|---|---|---|---|---|---|---|---|---|
| Batch 5A | 5-1 | 77.1 ± 0.1 | 5-Nitroisophthalic acid | 211.1 ± 0.1 | 176.1 ± 0.1 | 10 | Powder | 86.2 | 100.7 | 97.6 | 94.83 ± 7.64 (8.05) |
| | 5-2 | 77.1 ± 0.1 | DL-malic acid | 134 ± 0.1 | 176.1 ± 0.1 | 10 | Powder | 98.8 | 102.3 | 93.4 | 98.17 ± 4.48 (4.57) |
| | 5-3 | 77.1 ± 0.1 | 1-Hydroxy-2-Naphthoic acid | 188.1 ± 0.1 | 176.1 ± 0.1 | 10 | Powder | 90 | 85.6 | 86.8 | 87.47 ± 2.27 (2.60) |
| Batch 5B | 5-4 | 77.1 ± 0.1 | 5-Nitroisophthalic acid | 211.1 ± 0.1 | 176.1 ± 0.1 | 10 | Powder | 100.8 | 99.6 | 100.1 | 100.17 ± 0.60 (0.60) |
| | 5-5 | 77.1 ± 0.1 | DL-malic acid | 134 ± 0.1 | 176.1 ± 0.1 | 10 | Powder | 101.3 | 97.2 | 107.2 | 101.9 ± 5.03 (4.93) |
| | 5-6 | 77.1 ± 0.1 | 1-Hydroxy-2-Naphthoic acid | 188.1 ± 0.1 | 176.1 ± 0.1 | 10 | Powder | 99.2 | 97.7 | 96 | 97.63 ± 1.60 (1.64) |
| Batch 5C | 5-7 | 77.1 ± 0.1 | 5-Nitroisophthalic acid | 211.1 ± 0.1 | 176.1 ± 0.1 | 10 | Powder | 102.5 | 104 | 100.7 | 102.4 ± 1.65 (1.61) |
| | 5-8 | 77.1 ± 0.1 | DL-malic acid | 134 ± 0.1 | 176.1 ± 0.1 | 10 | Powder | 101.6 | 103.9 | 100.5 | 102 ± 1.73 (1.70) |
| | 5-9 | 77.1 ± 0.1 | 1-Hydroxy-2-Naphthoic acid | 188.1 ± 0.1 | 176.1 ± 0.1 | 10 | Powder | 88.3 | 94.5 | 90.8 | 91.2 ± 3.12 (3.42) |
| Batch 5D | 5-10 | 77.1 ± 0.1 | 5-Nitroisophthalic acid | 211.1 ± 0.1 | 176.1 ± 0.1 | 10 | Sticky | N/A | N/A | N/A | N/A |
| | 5-11 | 77.1 ± 0.1 | DL-malic acid | 134 ± 0.1 | 176.1 ± 0.1 | 10 | Powder | 99.8 | 94 | 96.2 | 96.67 ± 2.93 (3.03) |
| | 5-12 | 77.1 ± 0.1 | 1-Hydroxy-2-Naphthoic acid | 188.1 ± 0.1 | 176.1 ± 0.1 | 10 | Powder | 106.3 | 141.9 | 128.5 | 125.57 ± 17.98 (14.32) |

Table S6. Batch six synthesised in a 1:1:2 moral ratio of cysteamine: acid-coformer: ascorbic acid with the addition of 10 µl acetonitrile as the solvent (LAG) at rotation frequency of 20 Hz and milling duration of 5 minutes.

| Mixture Code | Cysteamine (mg) | Acid-coformer | Acid (mg) | Ascorbic acid (mg) | ACN (µl) | Texture | Free thiol % Yield R1 | Free thiol % Yield R2 | Free thiol % Yield R3 | Mean ± SD (RSD%) |
|---|---|---|---|---|---|---|---|---|---|---|
| 6-1 | 77.1 ± 0.1 | Stearic acid | 284.4 ± 0.1 | 352.2 ± 0.1 | 10 | Powder | 81.9 | 85.6 | 81.3 | 82.93 ± 2.33 (2.81) |
| 6-2 | 77.1 ± 0.1 | D-Isoascorbic acid | 176.1 ± 0.1 | 352.2 ± 0.1 | 10 | Gummy | N/A | N/A | N/A | N/A |
| 6-3 | 77.1 ± 0.1 | 5-Nitroisophthalic acid | 211.1 ± 0.1 | 352.2 ± 0.1 | 10 | Powder | 104.1 | 104.5 | 100.6 | 103.07 ± 1.75 (2.08) |
| 6-4 | 77.1 ± 0.1 | Pamoic acid | 388.3 ± 0.1 | 352.2 ± 0.1 | 10 | Powder | 82.7 | 91 | 86.7 | 86.8 ± 4.15 (4.78) |
| 6-5 | 77.1 ± 0.1 | DL-malic acid | 134 ± 0.1 | 352.2 ± 0.1 | 10 | Powder | 102.4 | 105.1 | 104.9 | 104.13 ± 1.23 (1.44) |
| 6-6 | 77.1 ± 0.1 | 2,5-Dihydroxybenzoic acid | 154.1 ± 0.1 | 352.2 ± 0.1 | 10 | Powder | 101.3 | 103.2 | 92.8 | 99.1 ± 5.54 (5.59) |
| 6-7 | 77.1 ± 0.1 | Glycolic acid | 76 ± 0.1 | 352.2 ± 0.1 | 10 | Sticky | N/A | N/A | N/A | N/A |
| 6-8 | 77.1 ± 0.1 | α-Ketoglutaric acid | 146 ± 0.1 | 352.2 ± 0.1 | 10 | Gummy | N/A | N/A | N/A | N/A |
| 6-9 | 77.1 ± 0.1 | 1-Hydroxy-2-Naphthoic acid | 188.1 ± 0.1 | 352.2 ± 0.1 | 10 | Powder | 104.1 | 101.5 | 103.4 | 103 ± 1.35 (1.31) |
| 6-10 | 77.1 ± 0.1 | Malonic acid | 104 ± 0.1 | 352.2 ± 0.1 | 10 | Sticky | N/A | N/A | N/A | N/A |
| 6-11 | 77.1 ± 0.1 | Phenoxyacetic acid | 152.1 ± 0.1 | 352.2 ± 0.1 | 10 | Powder | 98.2 | 96.3 | 97 | 97.17 ± 0.96 (0.99) |
| 6-12 | 77.1 ± 0.1 | 3-Hydroxy-2-naphthoic acid | 188.1 ± 0.1 | 352.2 ± 0.1 | 10 | Powder | 88.7 | 88.4 | 65.1 | 80.73 ± 13.54 (16.77) |
| 6-13 | 77.1 ± 0.1 | Fumaric acid | 116 ± 0.1 | 352.2 ± 0.1 | 10 | Powder | 62.3 | 61.1 | 47.1 | 56.83 ± 8.45 (14.87) |
| 6-14 | 77.1 ± 0.1 | L-(-)Malic acid | 134 ± 0.1 | 352.2 ± 0.1 | 10 | Powder | 99.2 | 95.7 | 96.8 | 97.23 ± 1.79 (1.84) |
| 6-15 | 77.1 ± 0.1 | 4-(Dimethylamino)Benzoic acid | 165.1 ± 0.1 | 352.2 ± 0.1 | 10 | Powder | 84 | 83 | 85.6 | 84.2 ± 1.31 (1.56) |
| 6-16 | 77.1 ± 0.1 | L-Ascorbic acid | 176.1 ± 0.1 | 352.2 ± 0.1 | 10 | Gummy | N/A | N/A | N/A | N/A |
| 6-17 | 77.1 ± 0.1 | Vanillic acid | 168.1 ± 0.1 | 352.2 ± 0.1 | 10 | Powder | 87.2 | 88 | 88.9 | 88.03 ± 0.85 (0.97) |
| 6-18 | 77.1 ± 0.1 | 3,5 Dihydroxybenzoic acid | 154.1 ± 0.1 | 352.2 ± 0.1 | 10 | Powder | 76 | 81.4 | 79.4 | 78.93 ± 2.73 (3.46) |
| 6-19 | 77.1 ± 0.1 | Gallic acid | 170.1 ± 0.1 | 352.2 ± 0.1 | 10 | Powder | 99.1 | 97.1 | 100 | 98.73 ± 1.48 (1.5) |
| 6-20 | 77.1 ± 0.1 | 4-Nitrobenzoic acid | 167.1 ± 0.1 | 352.2 ± 0.1 | 10 | Powder | 94.3 | 94.8 | 93.4 | 94.17 ± 0.71 (0.75) |
| 6-21 | 77.1 ± 0.1 | 3-3'-thiodipropionic acid | 178.2 ± 0.1 | 352.2 ± 0.1 | 10 | Gummy | N/A | N/A | N/A | N/A |
| 6-22 | 77.1 ± 0.1 | Trimesic acid | 210.1 ± 0.1 | 352.2 ± 0.1 | 10 | Powder | 93.7 | 88.2 | 94.5 | 92.13 ± 3.43 (3.72) |
| 6-23 | 77.1 ± 0.1 | L-Glutamic acid | 147.1 ± 0.1 | 352.2 ± 0.1 | 10 | Powder | 88.2 | 93.6 | 94.2 | 92 ± 3.3 (3.59) |
| 6-24 | 77.1 ± 0.1 | Zinc nitrate Hexahydrate | 284.4 ± 0.1 | 352.2 ± 0.1 | 10 | Oily | N/A | N/A | N/A | N/A |

Table S7. Batch seven synthesised in a 1:1:5 moral ratio of cysteamine: acid-coformer: ascorbic acid with the addition of 10 µl acetonitrile as the solvent (LAG) at rotation frequency of 20 Hz and milling duration of 5 minutes. PM represents physical mixture.

| Mixture Code | Cysteamine (mg) | Acid co-former | Acid-coformer (mg) | Ascorbic acid (mg) | ACN (µl) | Texture | Free thiol % Yield R1 | Free thiol % Yield R2 | Free thiol % Yield R3 | Mean ± SD (RSD%) |
|---|---|---|---|---|---|---|---|---|---|---|
| 7-1 | 77.1 ± 0.1 | 5-Nitroisophthalic acid | 211.1 ± 0.1 | 880.5 ± 0.1 | 10 | Powder | 98.7 | 92.6 | 86.2 | 92.5 ± 6.25 (6.76) |
| 7-2 | 77.1 ± 0.1 | DL-malic acid | 134 ± 0.1 | 880.5 ± 0.1 | 10 | Powder | 86 | 94.1 | 88.1 | 89.4 ± 4.20 (4.70) |
| 7-3 | 77.1 ± 0.1 | 1-Hydroxy-2-Naphthoic acid | 188.1 ± 0.1 | 880.5 ± 0.1 | 10 | Powder | PM* | PM* | PM* | PM* |

*Mainly Ascorbic acid peaks.

**Stacks of PXRD patterns for green cysteamine containing mixtures of batch 6.**

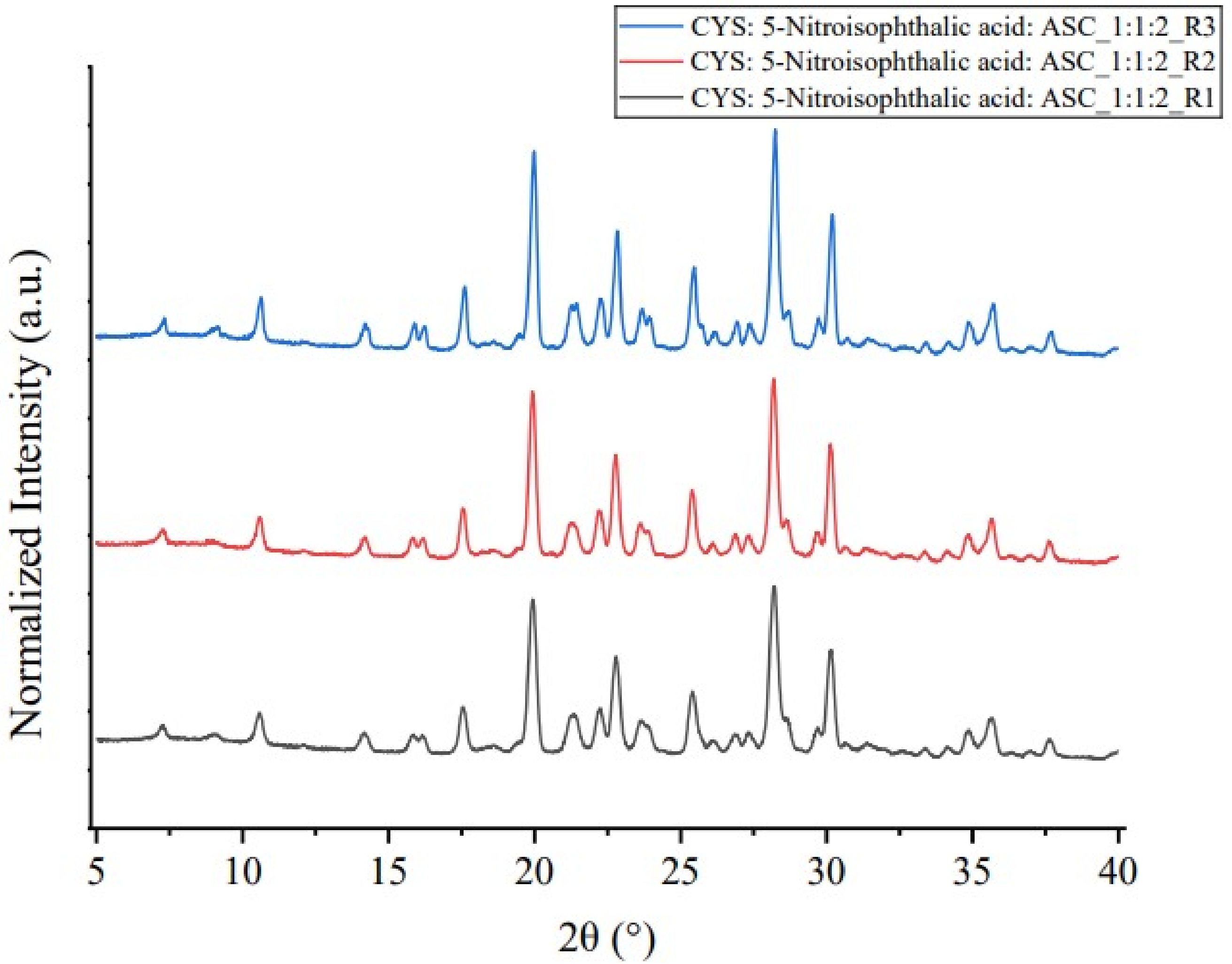


Figure S1. Presenting 3 repeats of the obtained ball milled mixture of cysteamine: 5-Nitroisophthalic acid: ascorbic acid with molar ratio of 1:1:2 (batch 6) and cysteamine percentage yield in range of 90-110%.

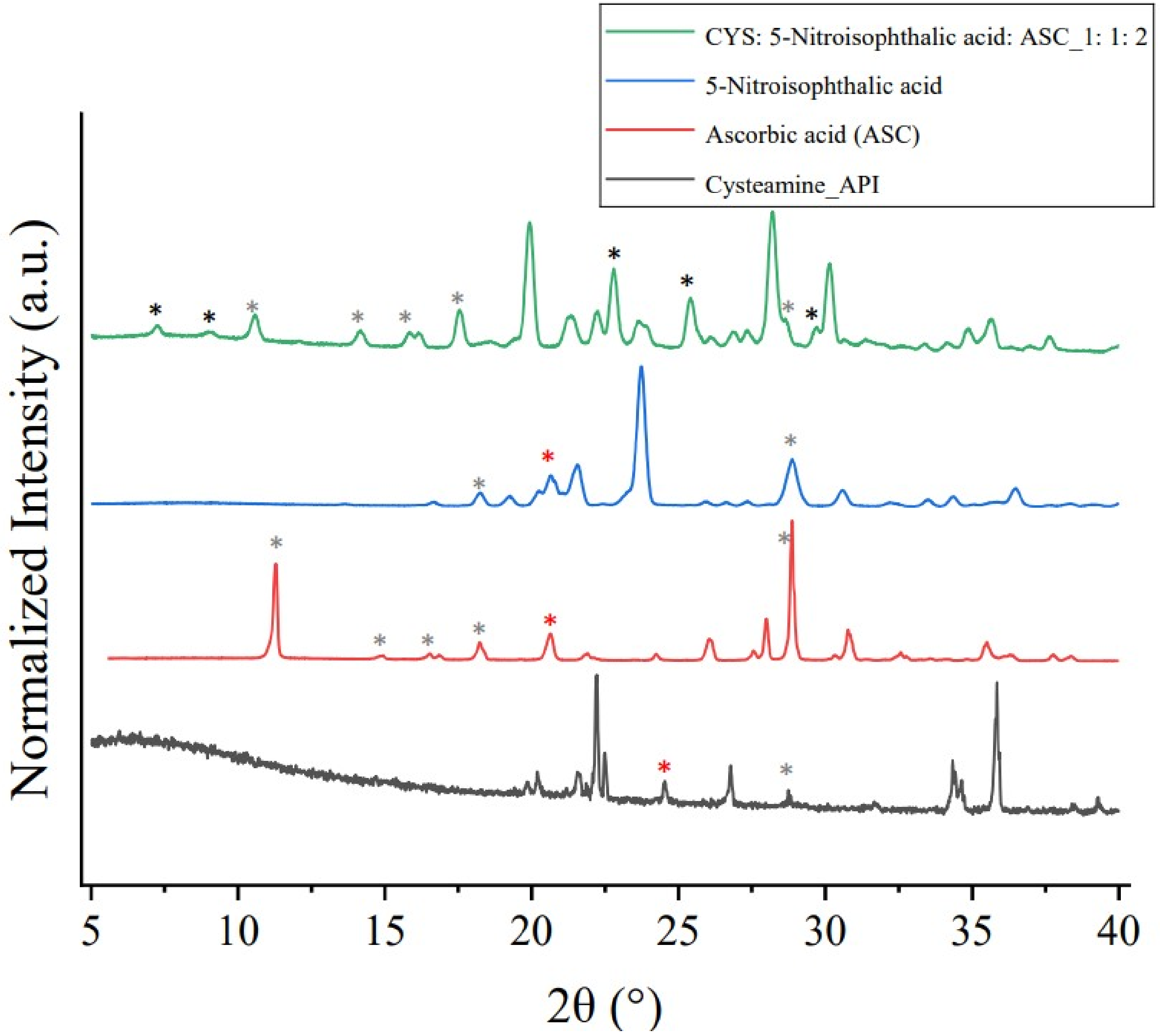


Figure S2. Presenting the stack of PXRD patterns for cysteamine: 5-Nitroisophthalic acid: ascorbic acid with molar ratio of 1:1:2 (batch 6). Comparing the starting materials vs the final mixture. * Black asterisk represents new peaks in the final mixture. * Grey asterisk represents the shifted peaks in the starting materials and final mixture. * Red asterisk represents the omitted peaks of starting materials in the final mixture.

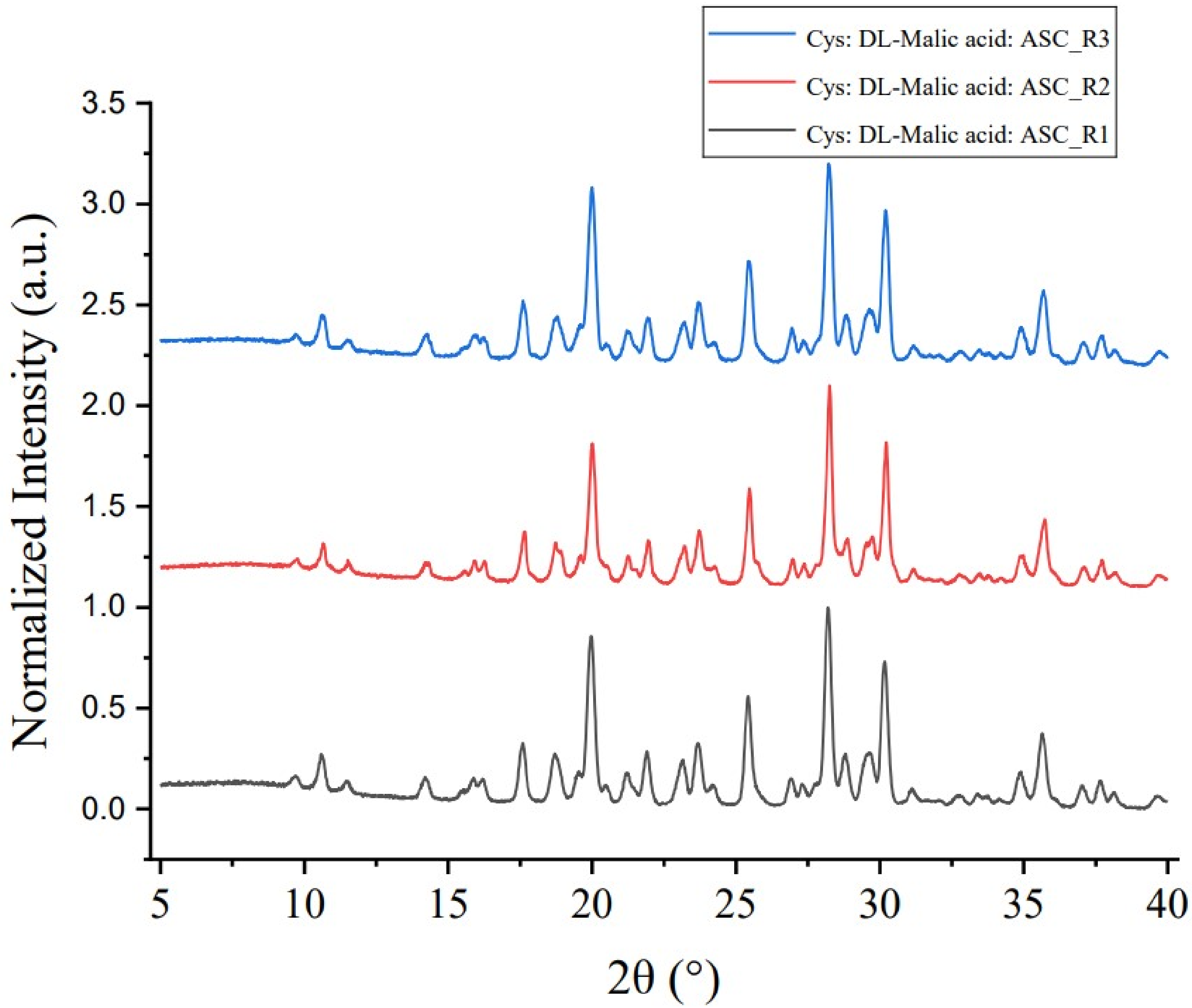


Figure S3. Presenting 3 repeats of the obtained ball milled mixture of cysteamine: DL-malic acid: ascorbic acid with molar ratio of 1:1:2 (batch 6) and cysteamine percentage yield in range of 90-110%.

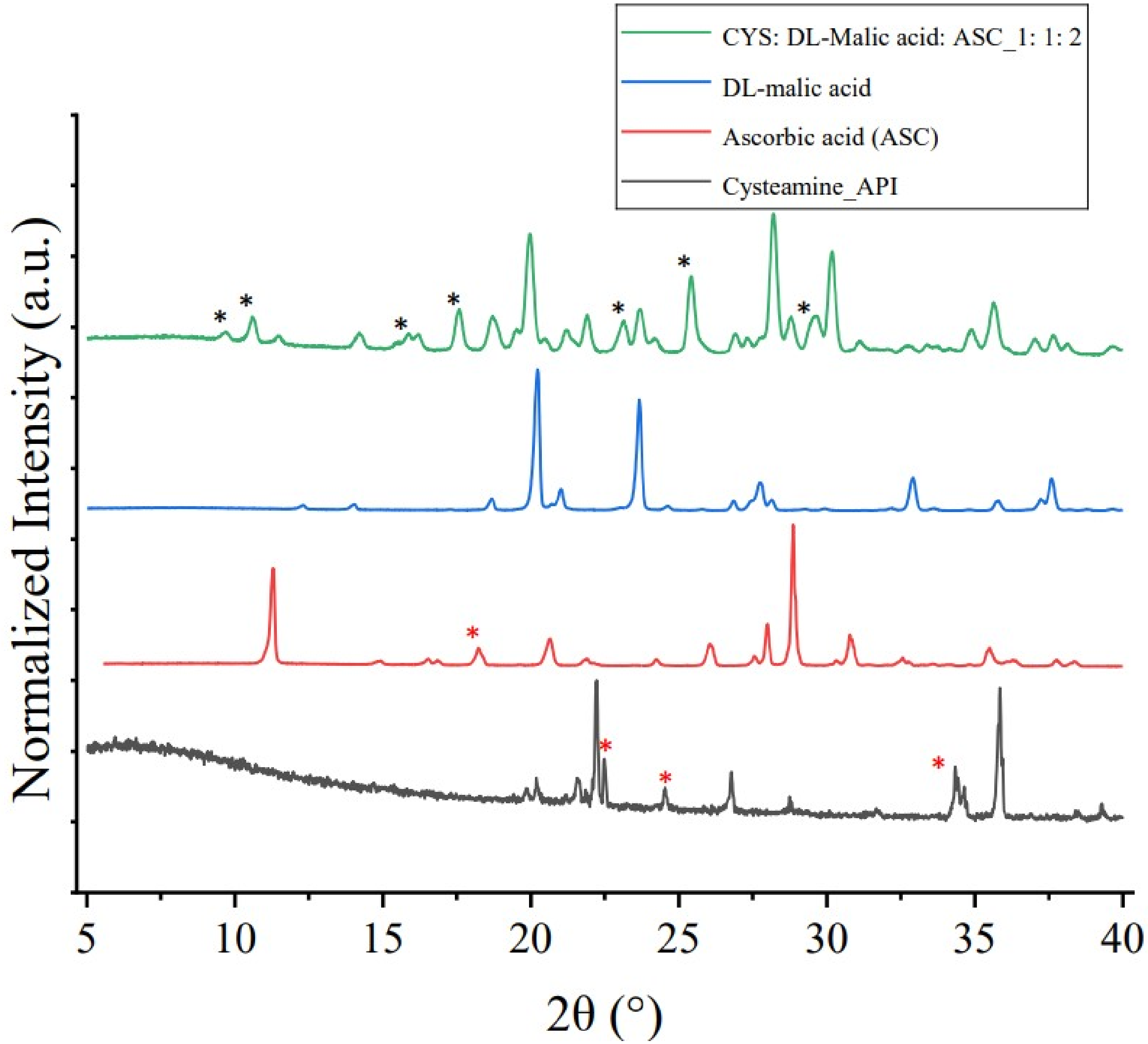


Figure S4. Presenting the stack of PXRD patterns for cysteamine: DL-malic acid: ascorbic acid with molar ratio of 1:1:2 (batch 6). Comparing the starting materials vs the final mixture. * Black asterisk represents new peaks in the final mixture. * Grey asterisk represents the shifted peaks in the starting materials and final mixture. * Red asterisk represents the omitted peaks of starting materials in the final mixture.

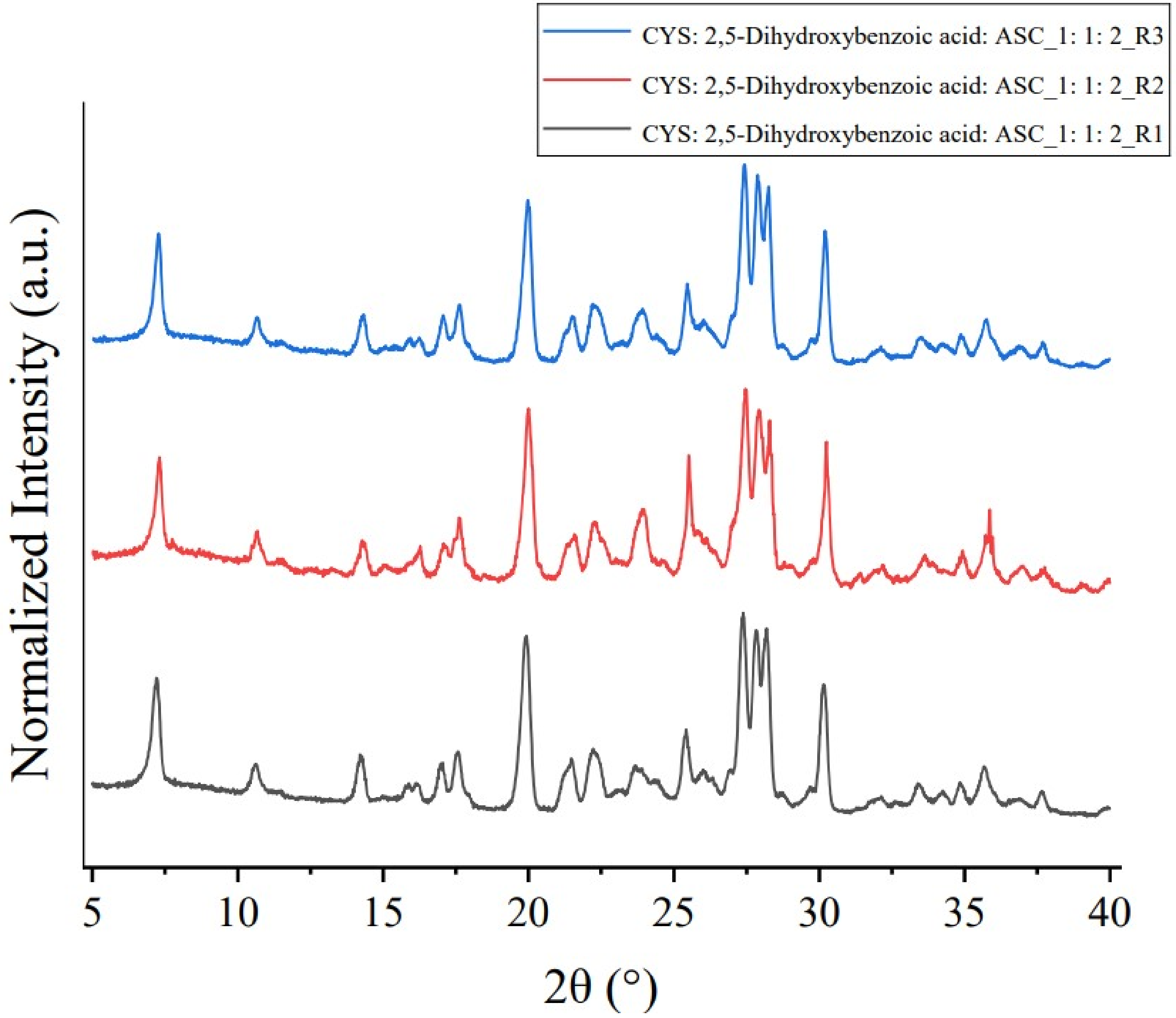


Figure S5. Presenting 3 repeats of the obtained ball milled mixture of cysteamine: 2,5-Dihydroxybenzoic acid: ascorbic acid with molar ratio of 1:1:2 (batch 6) and cysteamine percentage yield in range of 90-110%.

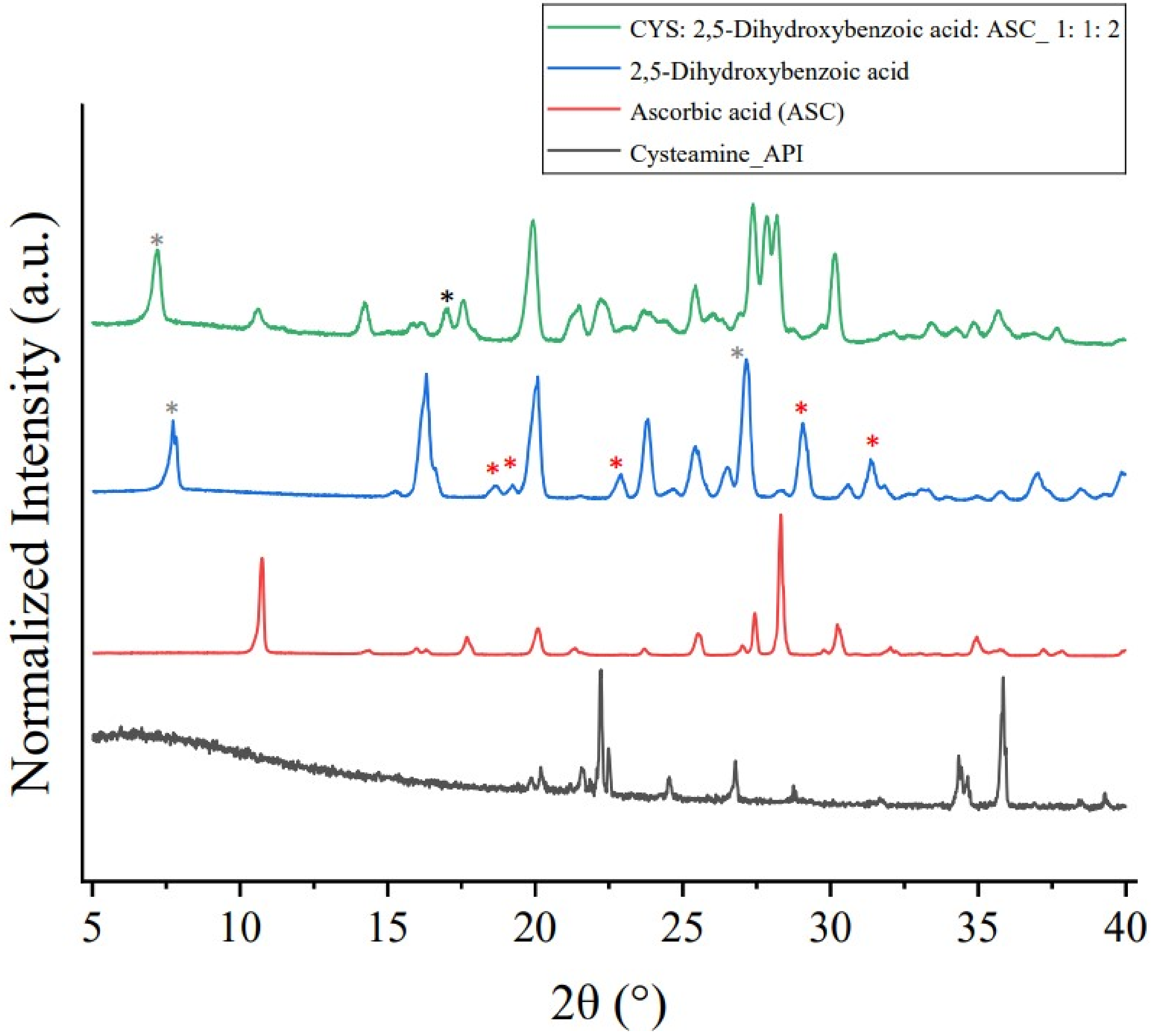


Figure S6. Presenting the stack of PXRD patterns for cysteamine: 2,5-Dihydroxybenzoic acid: ascorbic acid with molar ratio of 1:1:2 (batch 6). Comparing the starting materials vs the final mixture. * Black asterisk represents new peaks in the final mixture. * Grey asterisk represents the shifted peaks in the starting materials and final mixture. * Red asterisk represents the omitted peaks of starting materials in the final mixture.

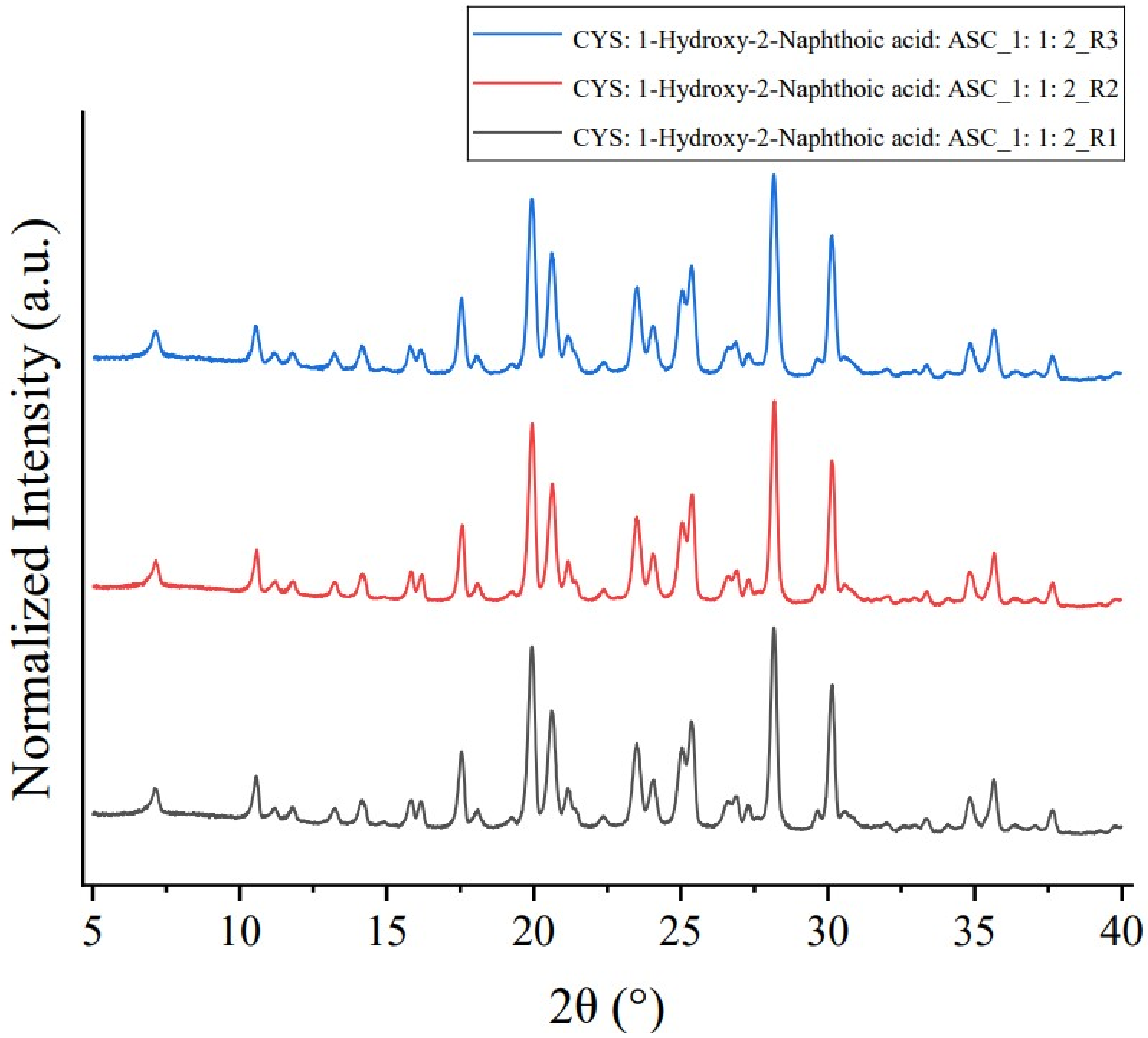


Figure S7. Presenting 3 repeats of the obtained ball milled mixture of cysteamine: 1-Hydroxy-2-Naphthoic acid: ascorbic acid with molar ratio of 1:1:2 (batch 6) and cysteamine percentage yield in range of 90-110%.

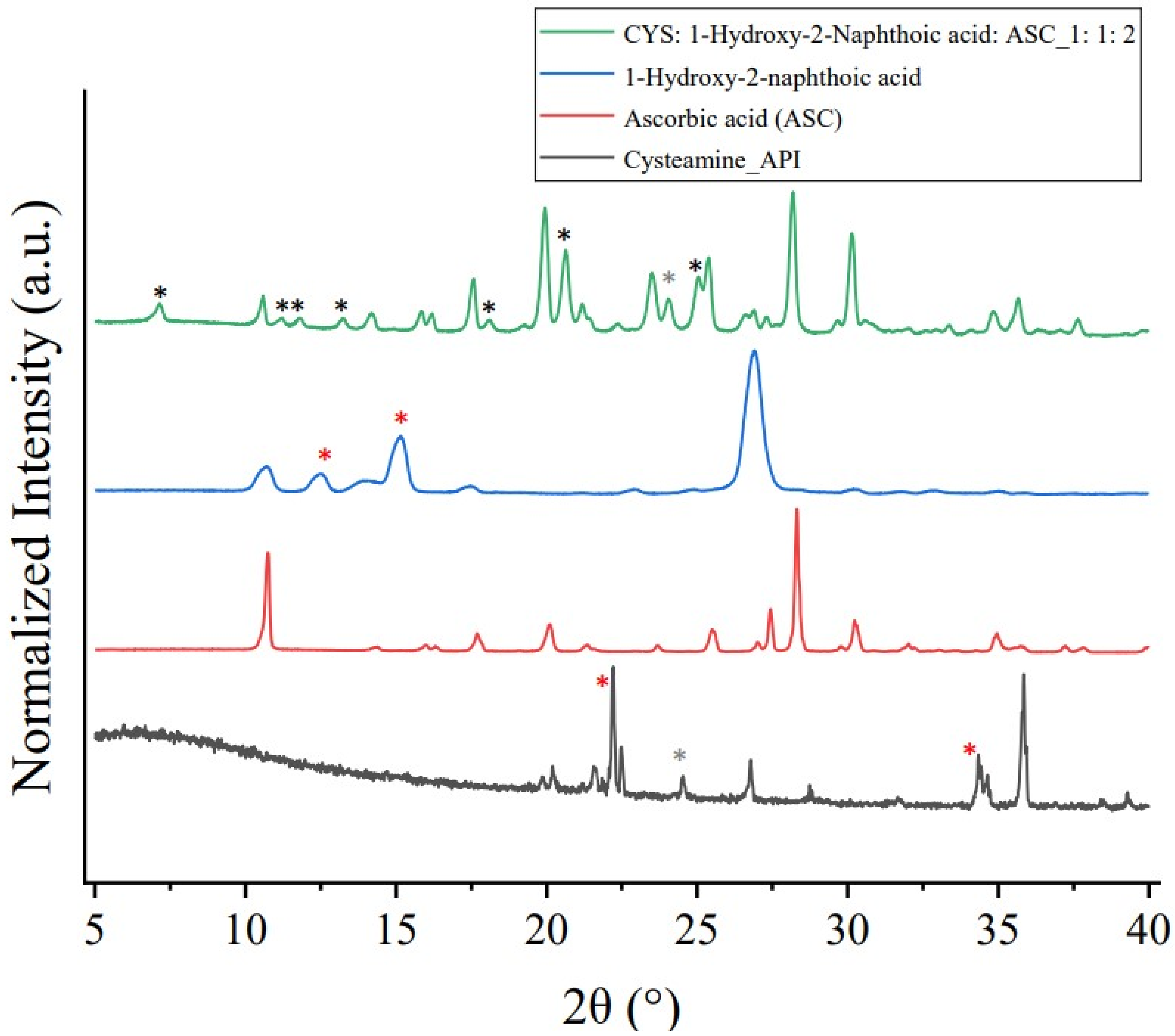


Figure S8. Presenting the stack of PXRD patterns for cysteamine: 1-Hydroxy-2-Naphthoic acid: ascorbic acid with molar ratio of 1:1:2 (batch 6). Comparing the starting materials vs the final mixture. * Black asterisk represents new peaks in the final mixture. * Grey asterisk represents the shifted peaks in the starting materials and final mixture. * Red asterisk represents the omitted peaks of starting materials in the final mixture.

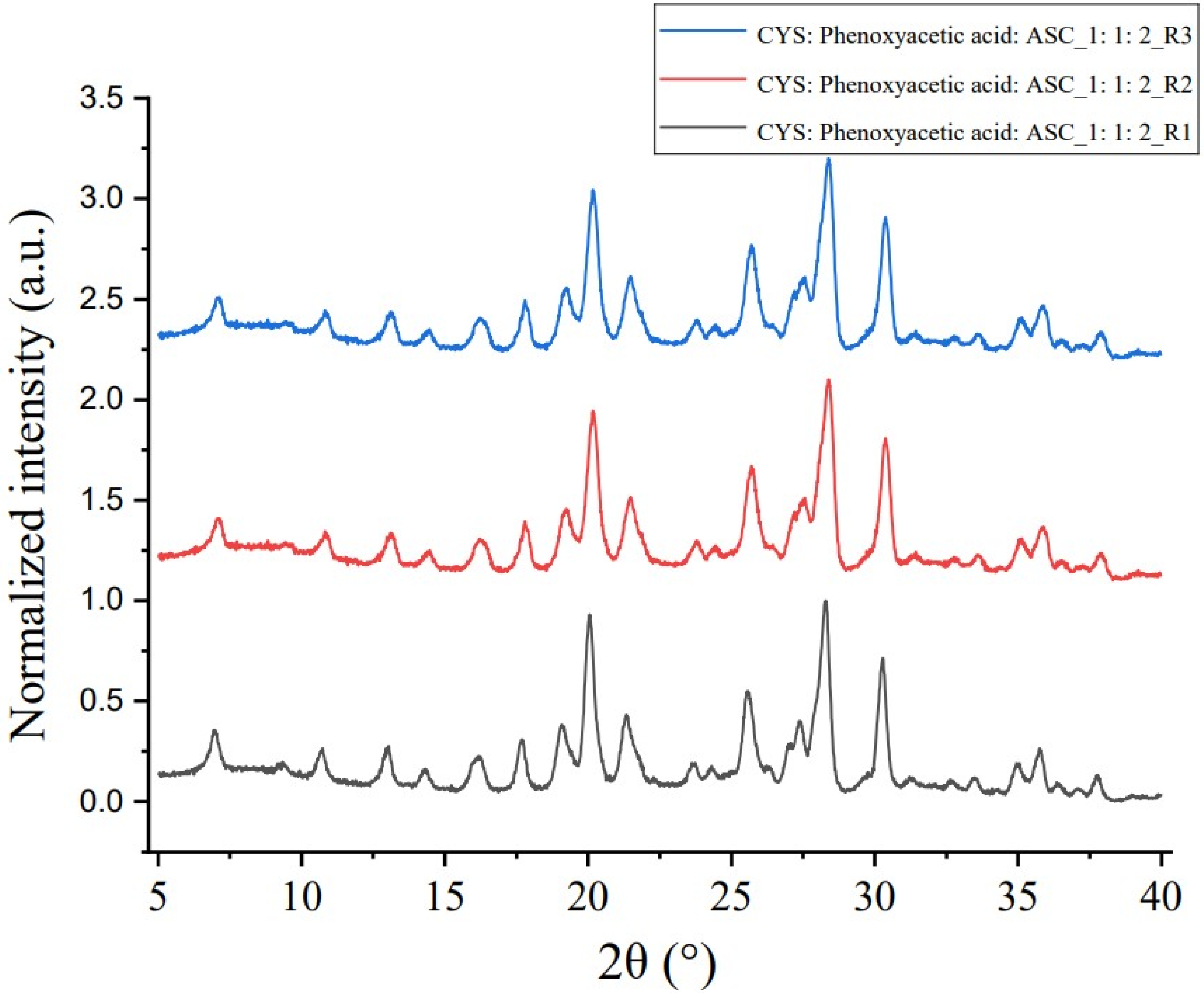


Figure S9. Presenting 3 repeats of the obtained ball milled mixture of cysteamine: Phenoxyacetic acid: ascorbic acid with molar ratio of 1:1:2 (batch 6) and cysteamine percentage yield in range of 90-110%.

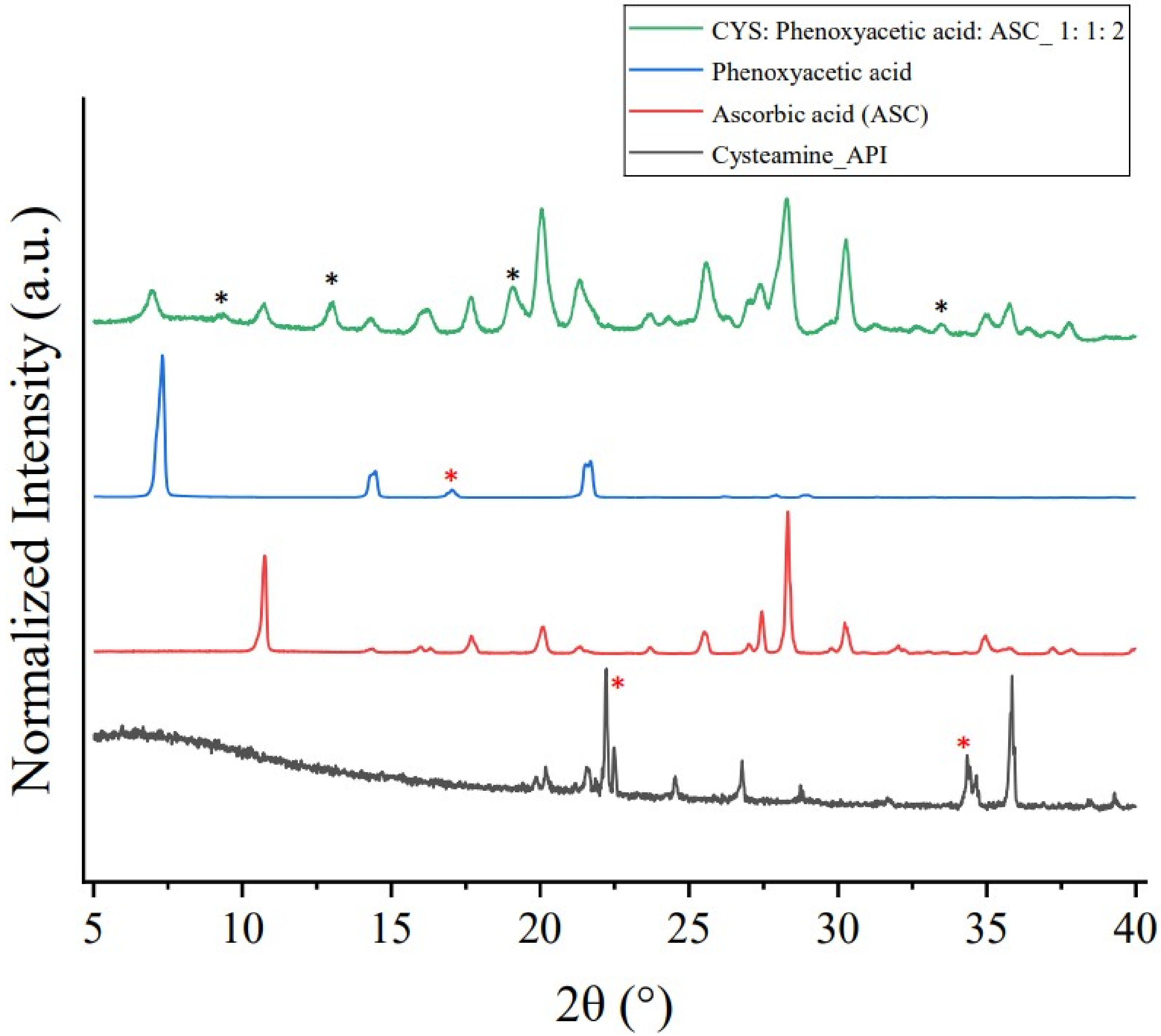


Figure S10. Presenting the stack of PXRD patterns for cysteamine: Phenoxyacetic acid: ascorbic acid with molar ratio of 1:1:2 (batch 6). Comparing the starting materials vs the final mixture. * Black asterisk represents new peaks in the final mixture. * Grey asterisk represents the shifted peaks in the starting materials and final mixture. * Red asterisk represents the omitted peaks of starting materials in the final mixture.

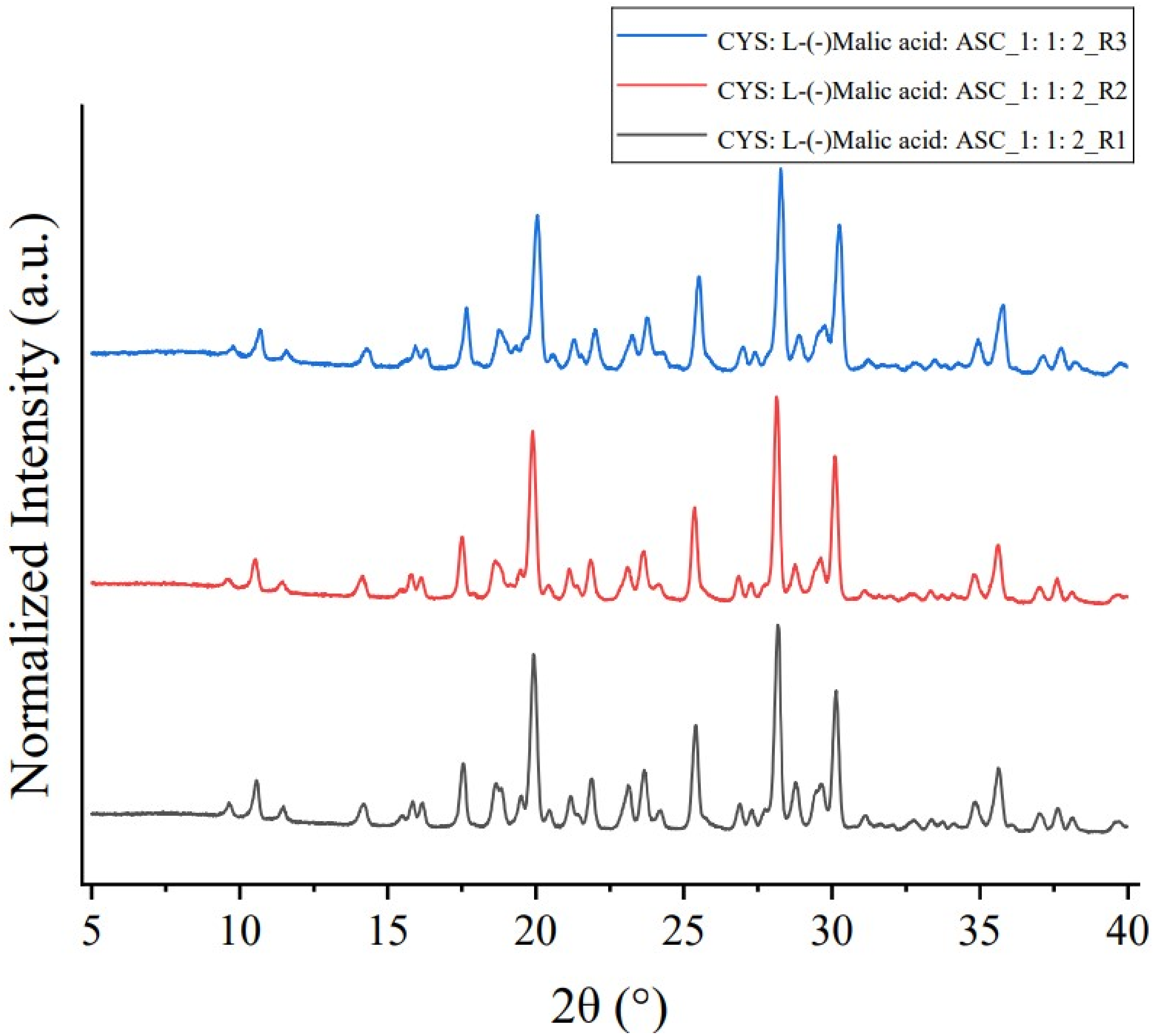


Figure S11. Presenting 3 repeats of the obtained ball milled mixture of cysteamine: L-(-)Malic acid: ascorbic acid with molar ratio of 1:1:2 (batch 6) and cysteamine percentage yield in range of 90-110%.

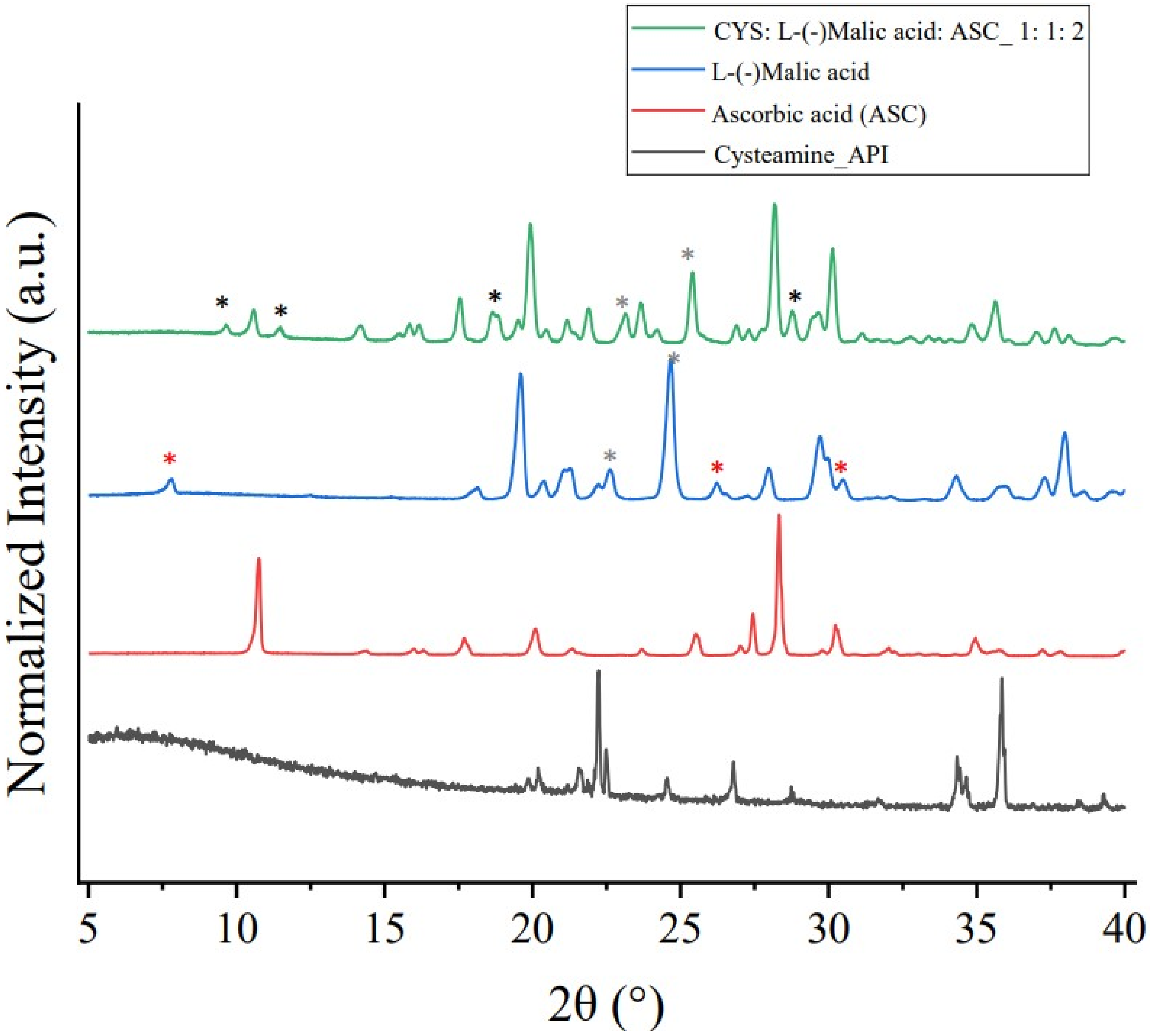


Figure S12. Presenting the stack of PXRD patterns for cysteamine: L-(-)Malic acid: ascorbic acid with molar ratio of 1:1:2 (batch 6). Comparing the starting materials vs the final mixture. * Black asterisk represents new peaks in the final mixture. * Grey asterisk represents the shifted peaks in the starting materials and final mixture. * Red asterisk represents the omitted peaks of starting materials in the final mixture.

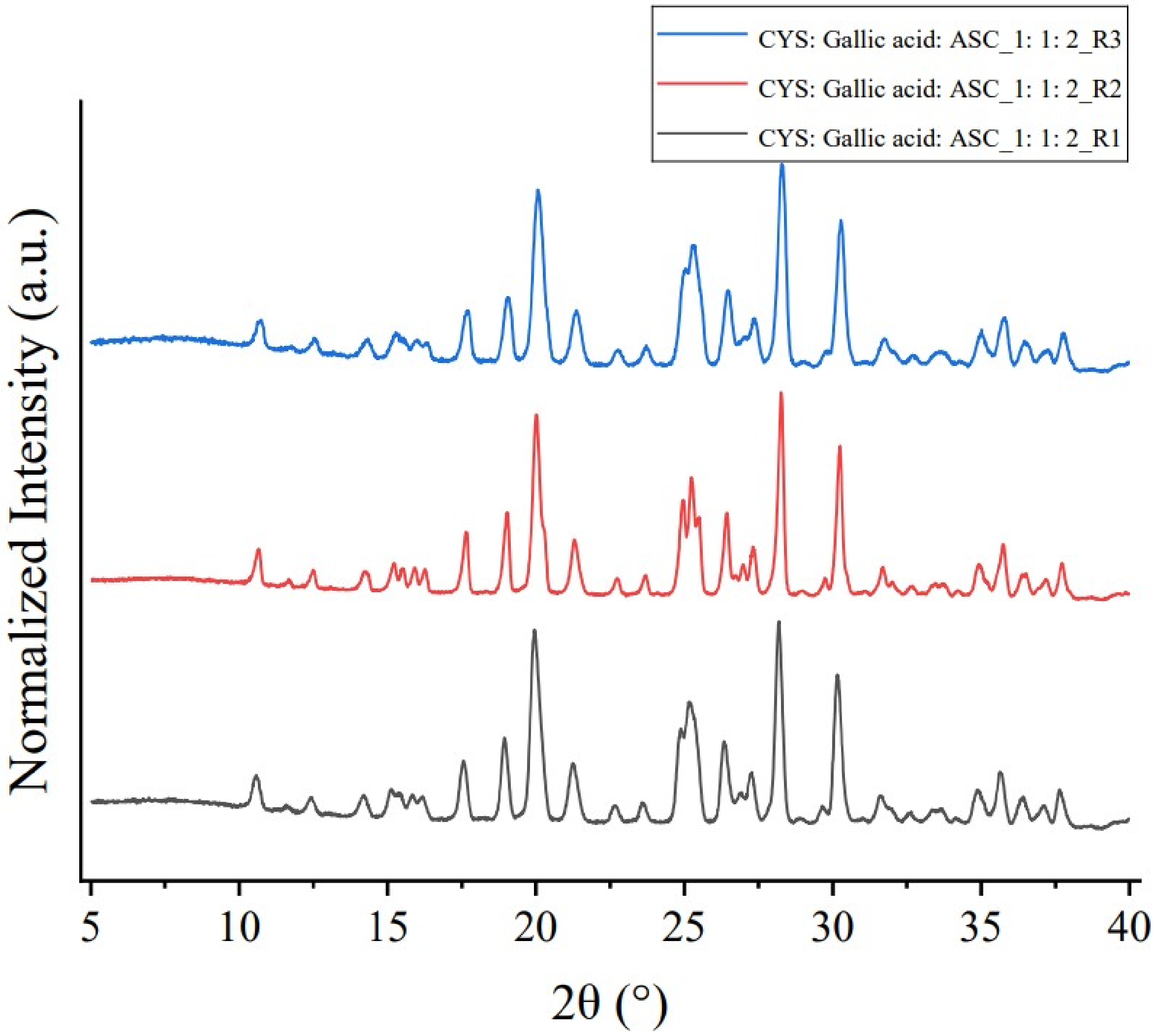


Figure S13. Presenting 3 repeats of the obtained ball milled mixture of cysteamine: Gallic acid: ascorbic acid with molar ratio of 1:1:2 (batch 6) and cysteamine percentage yield in range of 90-110%.

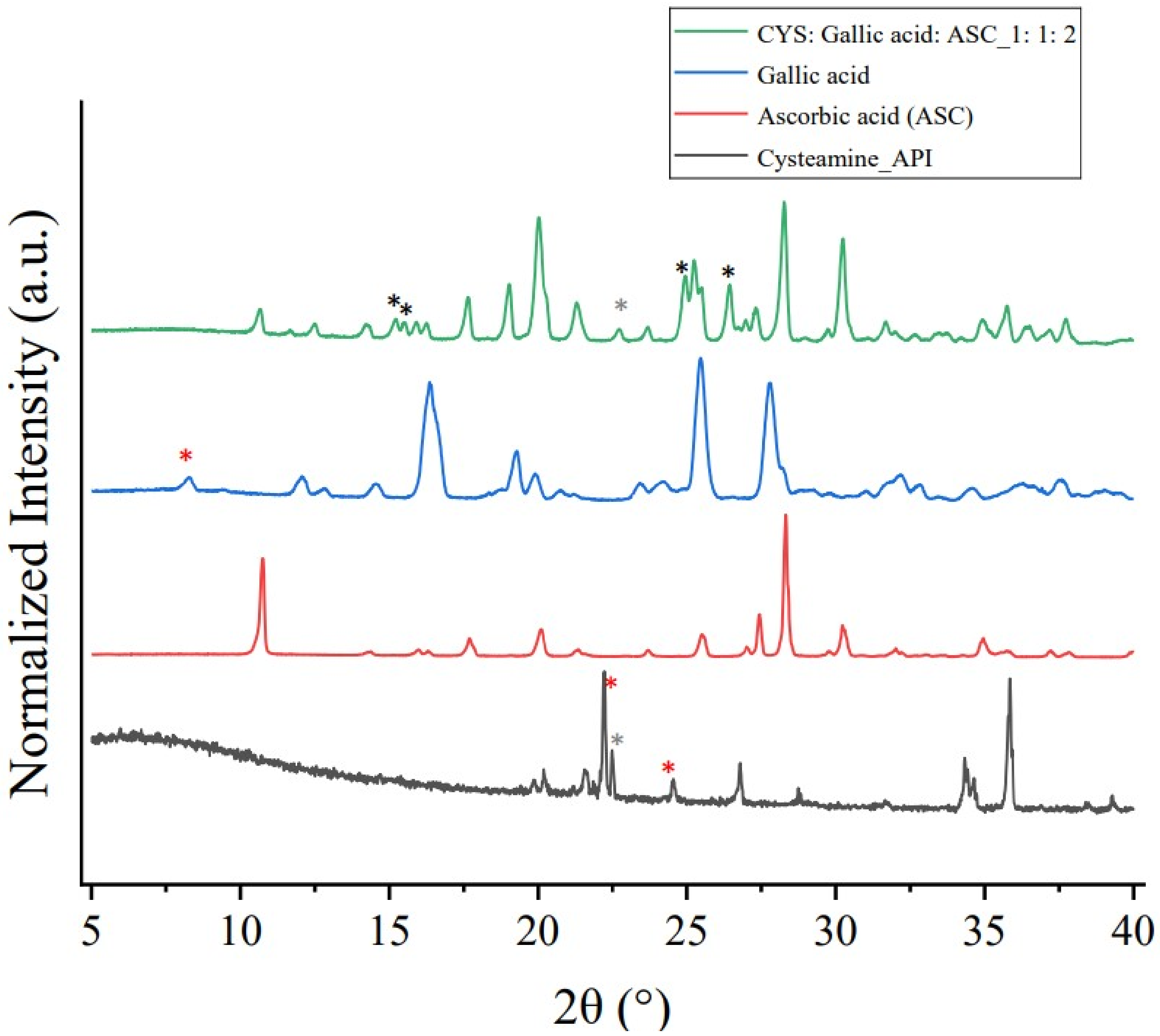


Figure S14. Presenting the stack of PXRD patterns for cysteamine: galic acid: ascorbic acid with molar ratio of 1:1:2 (batch 6). Comparing the starting materials vs the final mixture. * Black asterisk represents new peaks in the final mixture. * Grey asterisk represents the shifted peaks in the starting materials and final mixture. * Red asterisk represents the omitted peaks of starting materials in the final mixture.

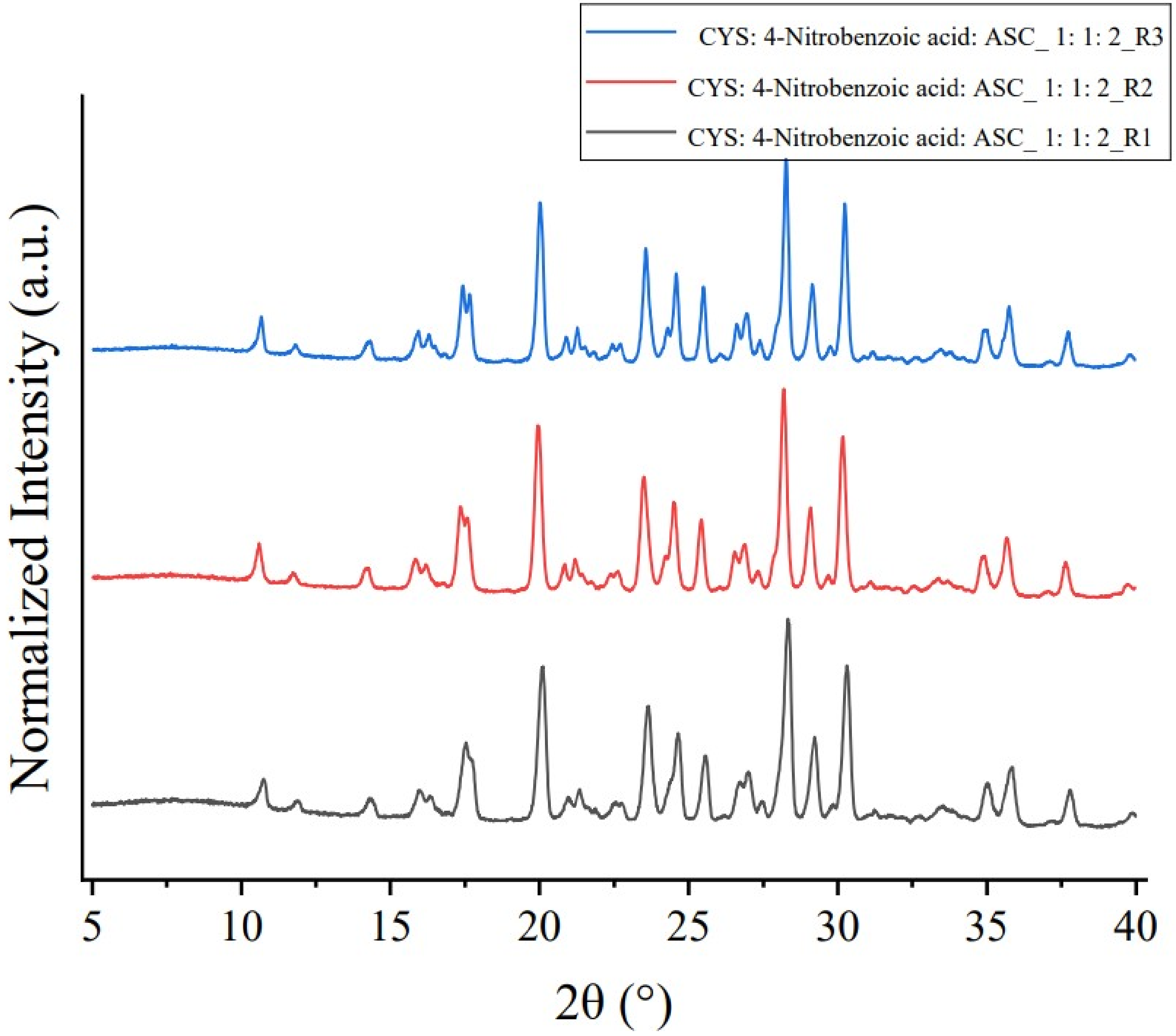


Figure S15. Presenting 3 repeats of the obtained ball milled mixture of cysteamine: 4-Nitrobenzoic acid: ascorbic acid with molar ratio of 1:1:2 (batch 6) and cysteamine percentage yield in range of 90-110%.

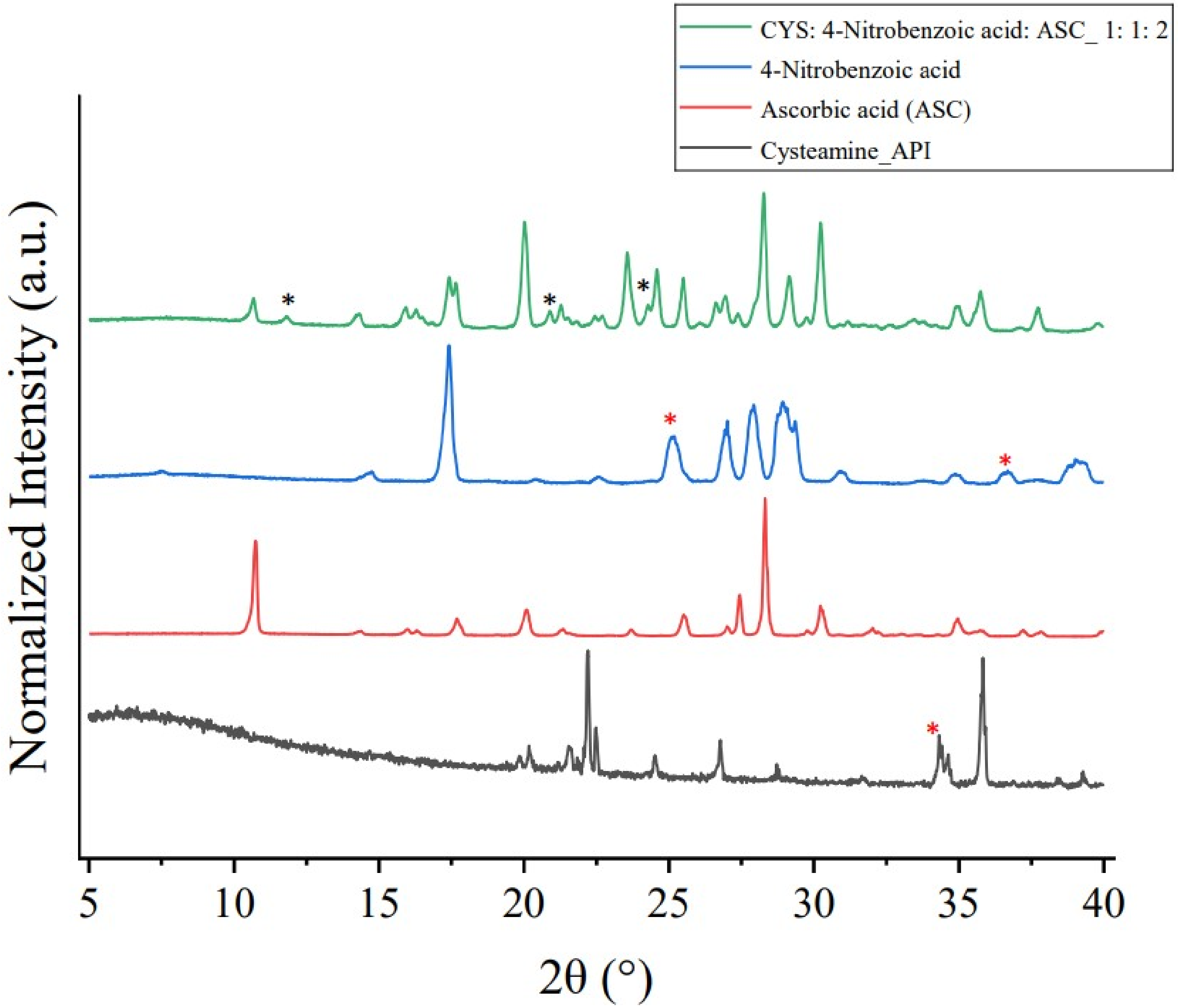


Figure S16. Presenting the stack of PXRD patterns for cysteamine: 4-Nitrobenzoic acid: ascorbic acid with molar ratio of 1:1:2 (batch 6). Comparing the starting materials vs the final mixture. * Black asterisk represents new peaks in the final mixture. * Grey asterisk represents the shifted peaks in the starting materials and final mixture. * Red asterisk represents the omitted peaks of starting materials in the final mixture.

**Stacks of PXRD patterns investigating ascorbic acid addition vs ball to powder ratio**

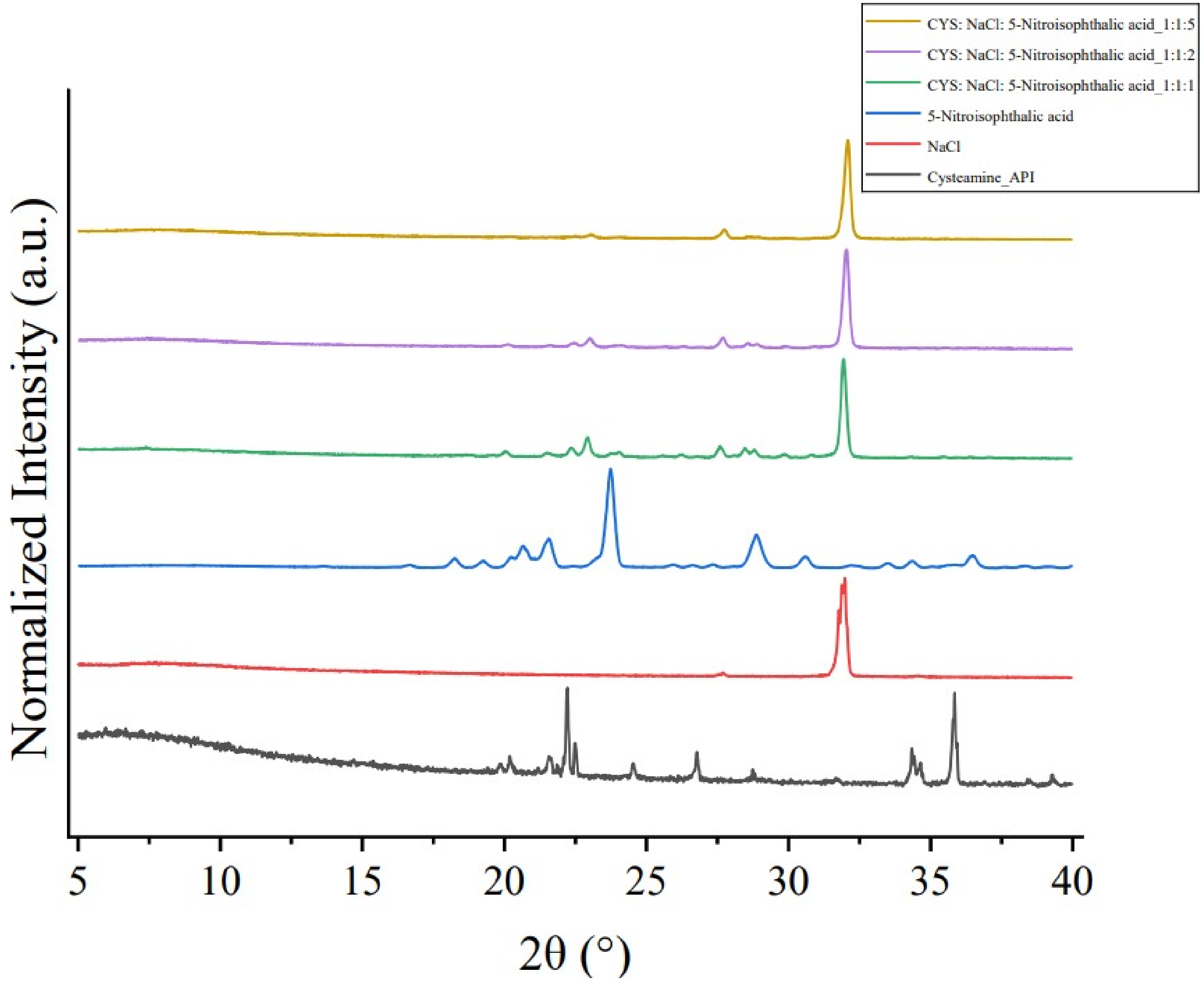


Figure S17. Investigating the influence of ascorbic acid addition to cysteamine and 5-Nitroisophthalic acid mixtures in LAG condition vs potential ball to powder ratio effect, by replacing ASC with inert NaCl. The obtained PXRDs for cysteamine: coformer: NaCl in variable molar ratios compared with starting

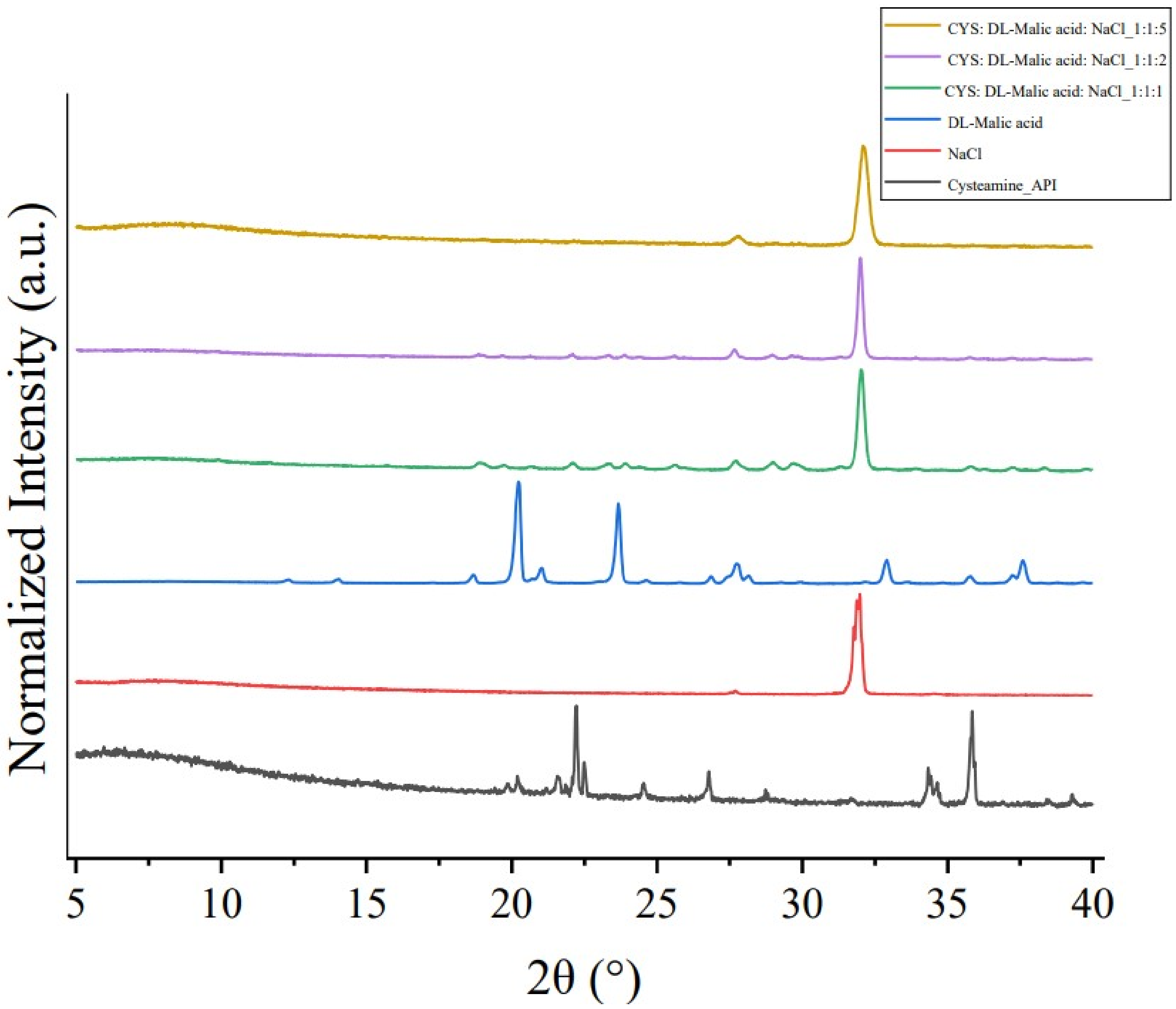


Figure S18. Investigating the influence of ascorbic acid addition to cysteamine and DL-malic acid mixtures in LAG condition vs potential ball to powder ratio effect, by replacing ASC with inert NaCl. The obtained PXRDs for cysteamine: coformer: NaCl in variable molar ratios compared with starting materials.

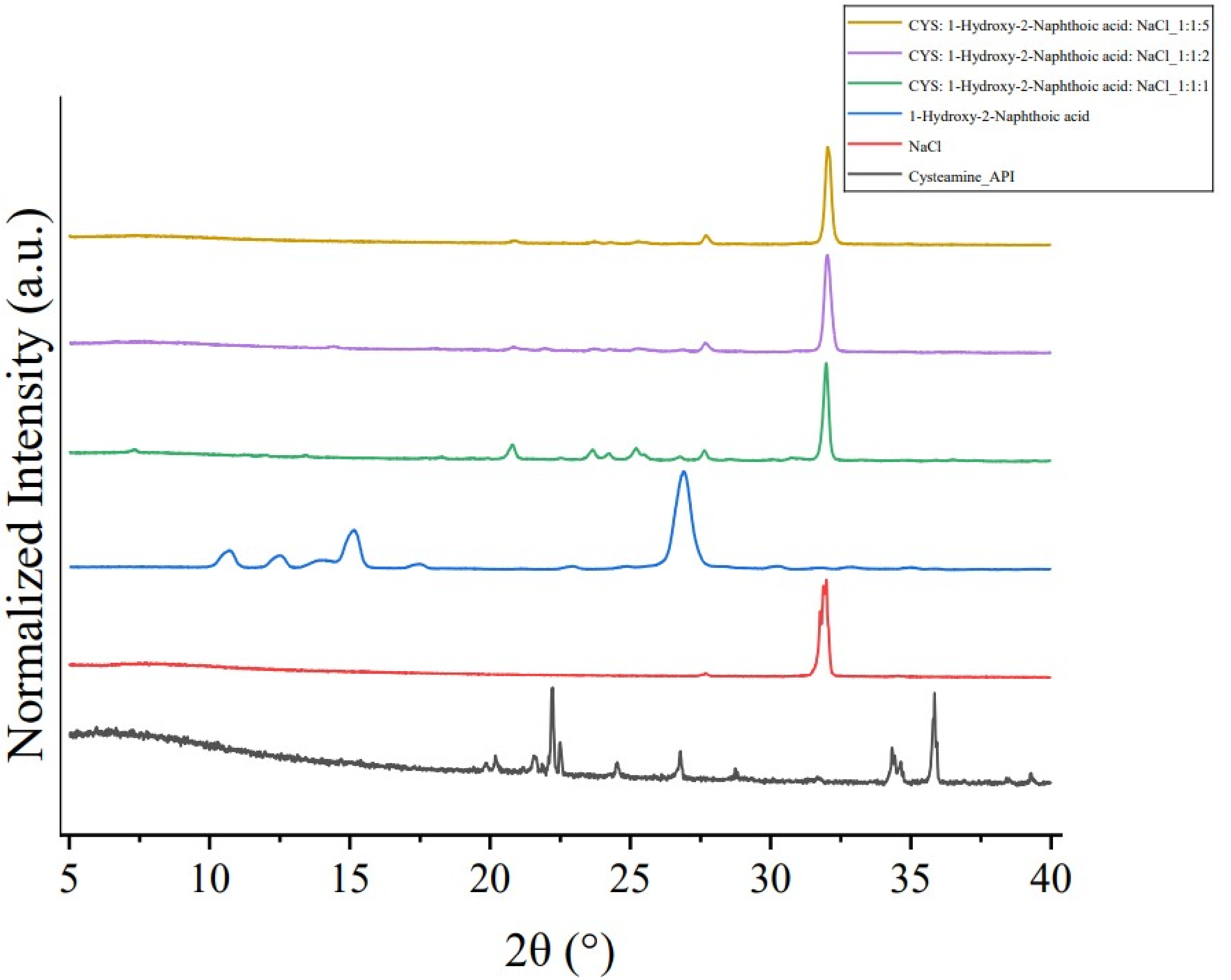


Figure S19. Investigating the influence of ascorbic acid addition to cysteamine and 1-Hydroxy-2-Naphthoic acid mixtures in LAG condition vs potential ball to powder ratio effect, by replacing ASC with inert NaCl. The obtained PXRDs for cysteamine: coformer: NaCl in variable molar ratios compared with starting materials.

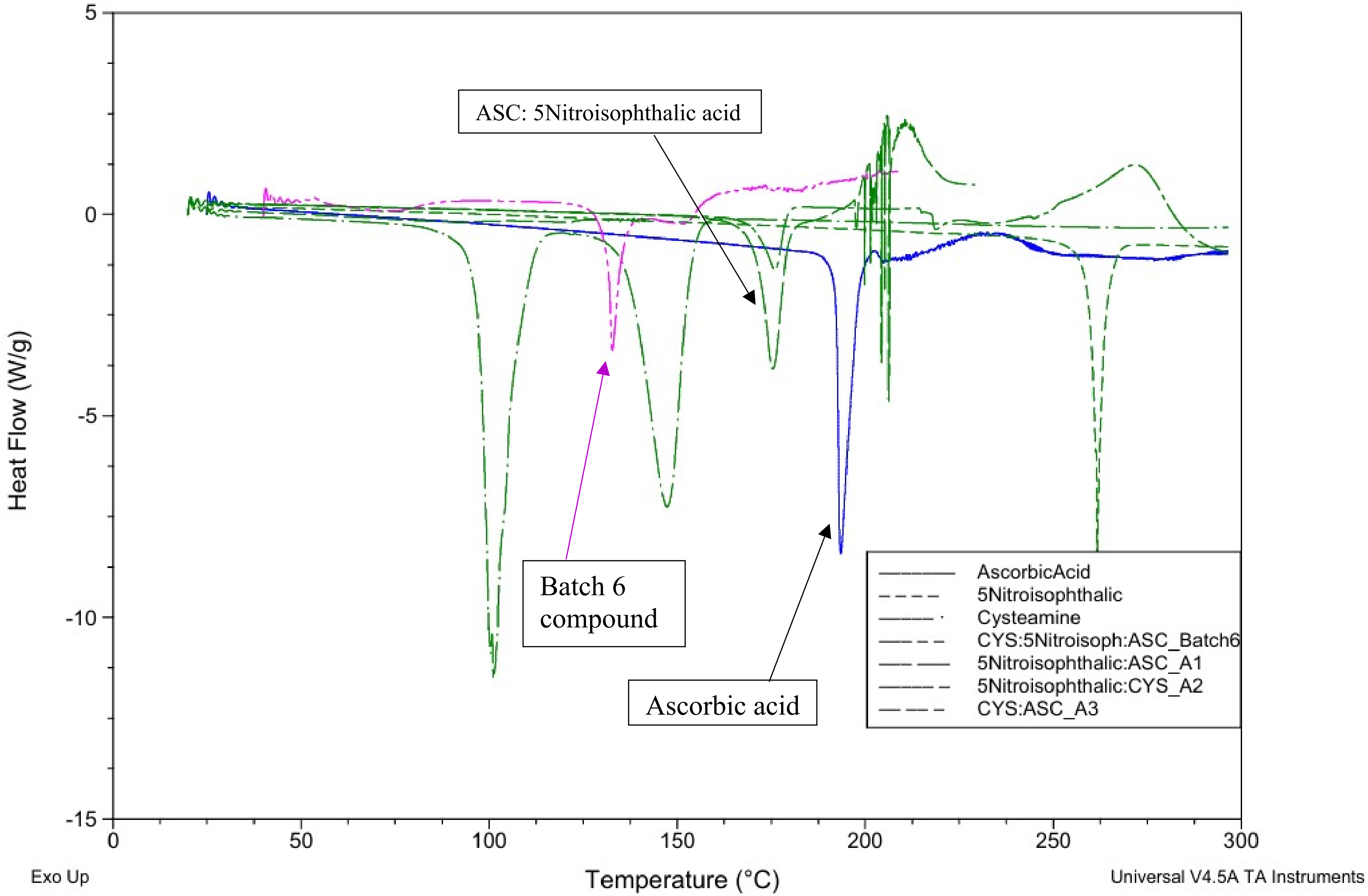


Figure S20. Stacked Differential Scanning Calorimetry (DSC) profile of CYS: 5-Nitroisophthalic acid: ASC molar ratio 1: 1: 2 (batch 6). DSC thermograms of starting materials along DSC thermograms for NaCl: 5-Nitroisophthalic acid: ASC 1: 1: 2 (A1), 5-Nitroisophthalic acid: CYS: NaCl 1: 1: 2 (A2), and NaCl: CYS: ASC 1: 1: 2 (A3) are presented on the same graph. The final ball milled mixture demonstrates a clear distinct endothermic peak @ about 130ºC (coloured in pink).

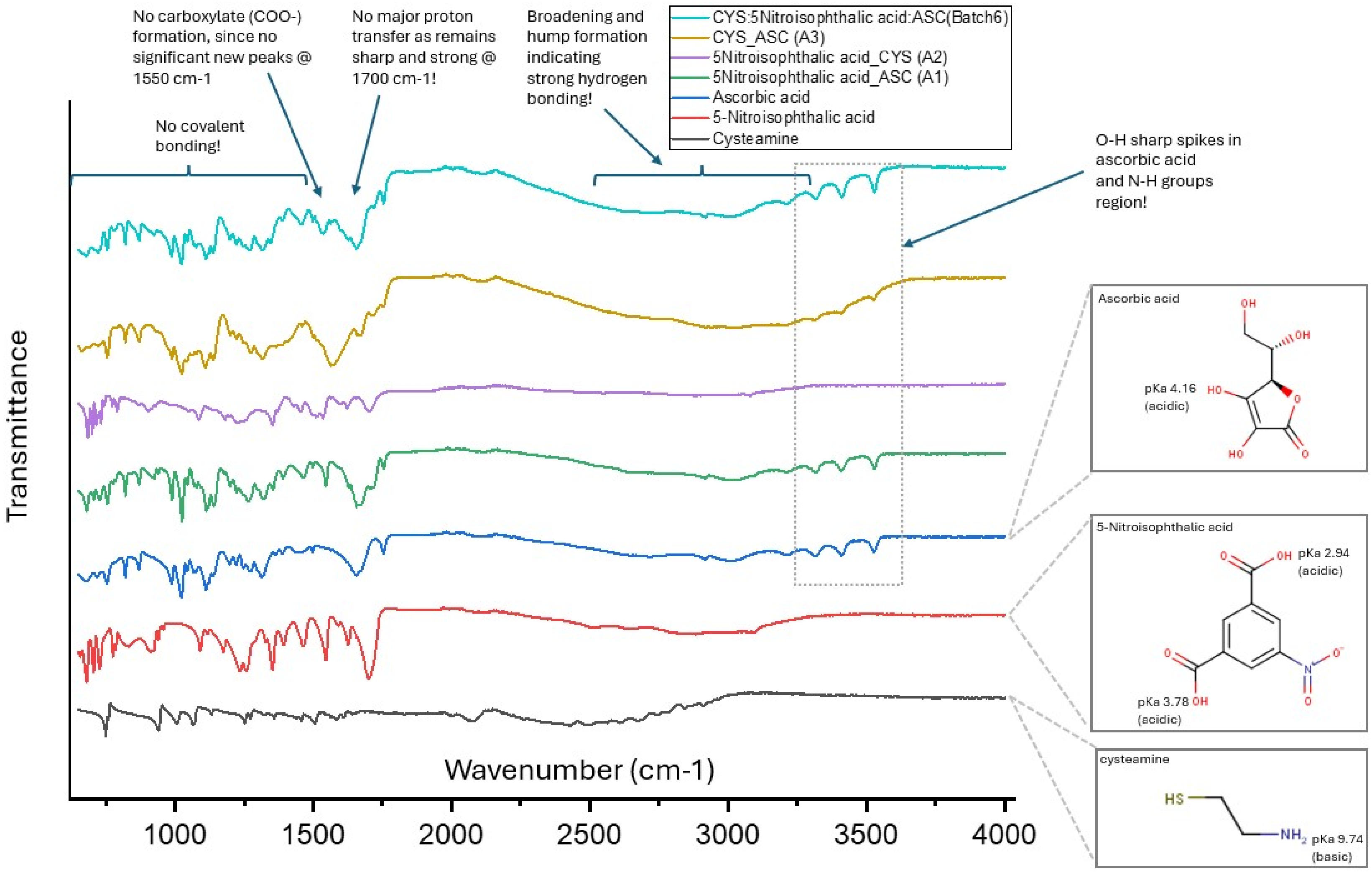


Figure S21. Stacked Fourier Transform Infrared Spectroscopy (FTIR) profile of CYS: 5-Nitroisophthalic acid: ASC molar ratio 1: 1: 2 (batch 6). FTIR spectrums of starting materials along FTIR spectrums for NaCl: 5-Nitroisophthalic acid: ASC 1: 1: 2 (A1), 5-Nitroisophthalic acid: CYS: NaCl 1: 1: 2 (A2), and NaCl: CYS: ASC 1: 1: 2 (A3) are presented.

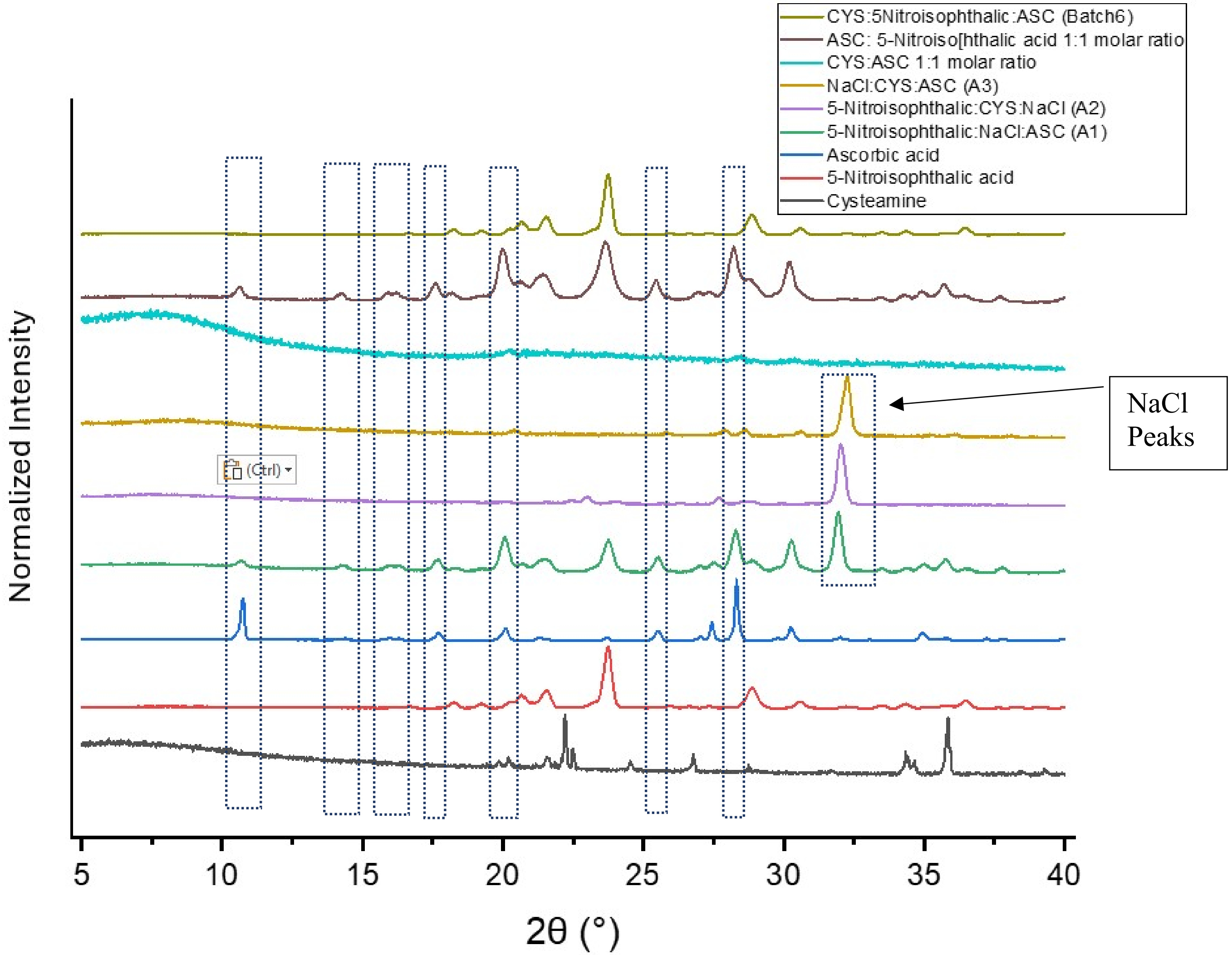


Figure S22. PXRD stack of starting materials along potential binary crystals of contents against the final green mixture of cysteamine:5-Nitroisophthalic acid:ascorbic acid in the ratio of 1:1:2 molar ratio. For further clarification PXRD of cysteamine: ascorbic acid and ascorbic acid: 5-Nitroisophthalic acid both in 1:1 molar ratios also added. NaCl as an inert compound used to replace each component individually (exact mass of replaced component) in exact milling conditions to batch 6 compound to facilitate comparison of potential binary crystals (A1-A3). The newly formed or omitted peaks, in the final green mixtures (batch 6) compared to the other PXRD patterns, are highlighted using dash line boxes.

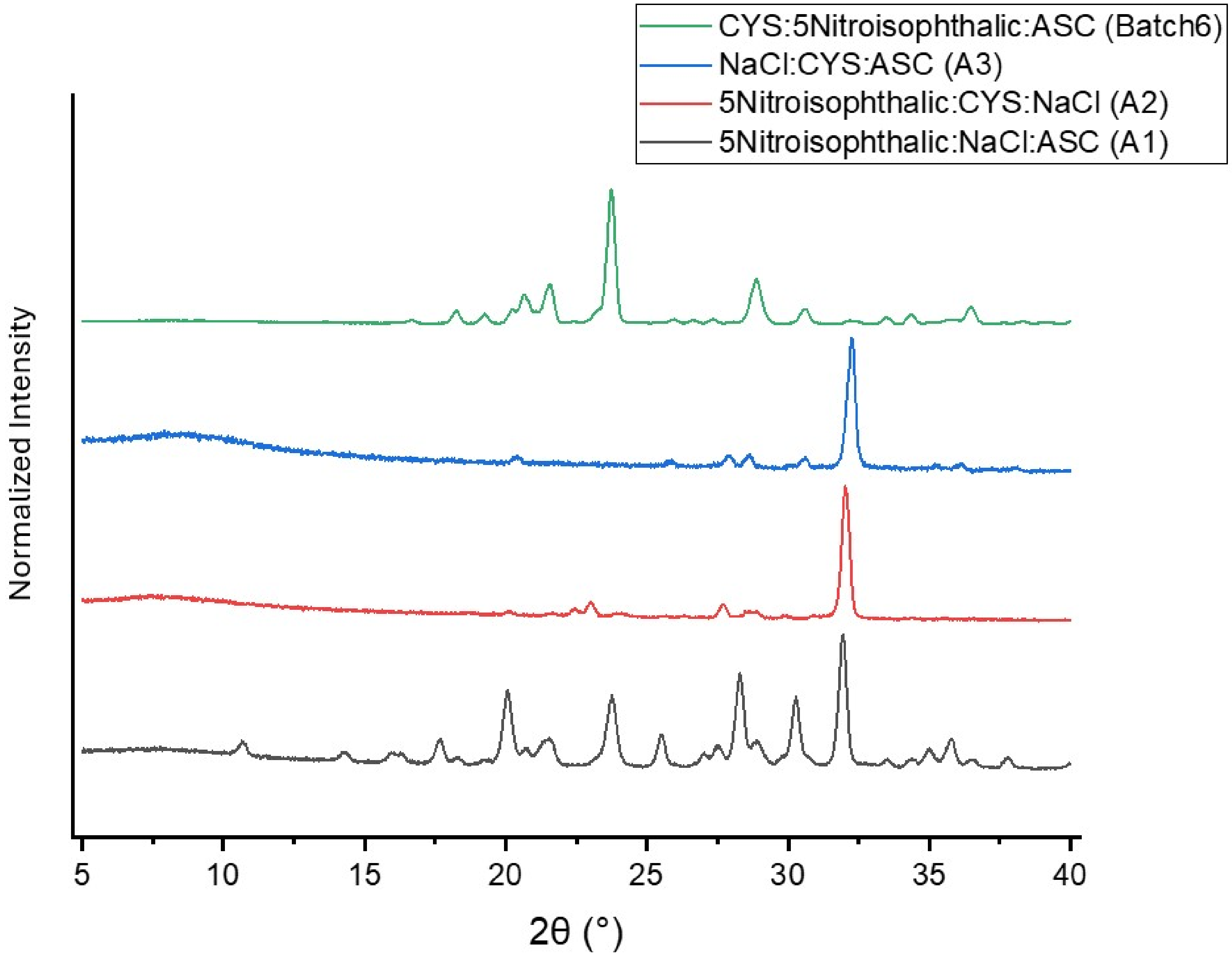


Figure S23. Magnifying the stacked PXRD patterns of A1, A2, A3 vs the final ball milled mixture of cysteamine: 5-Nitroisophthalic acid: ascorbic acid 1:1:2 molar ratio (batch 6).

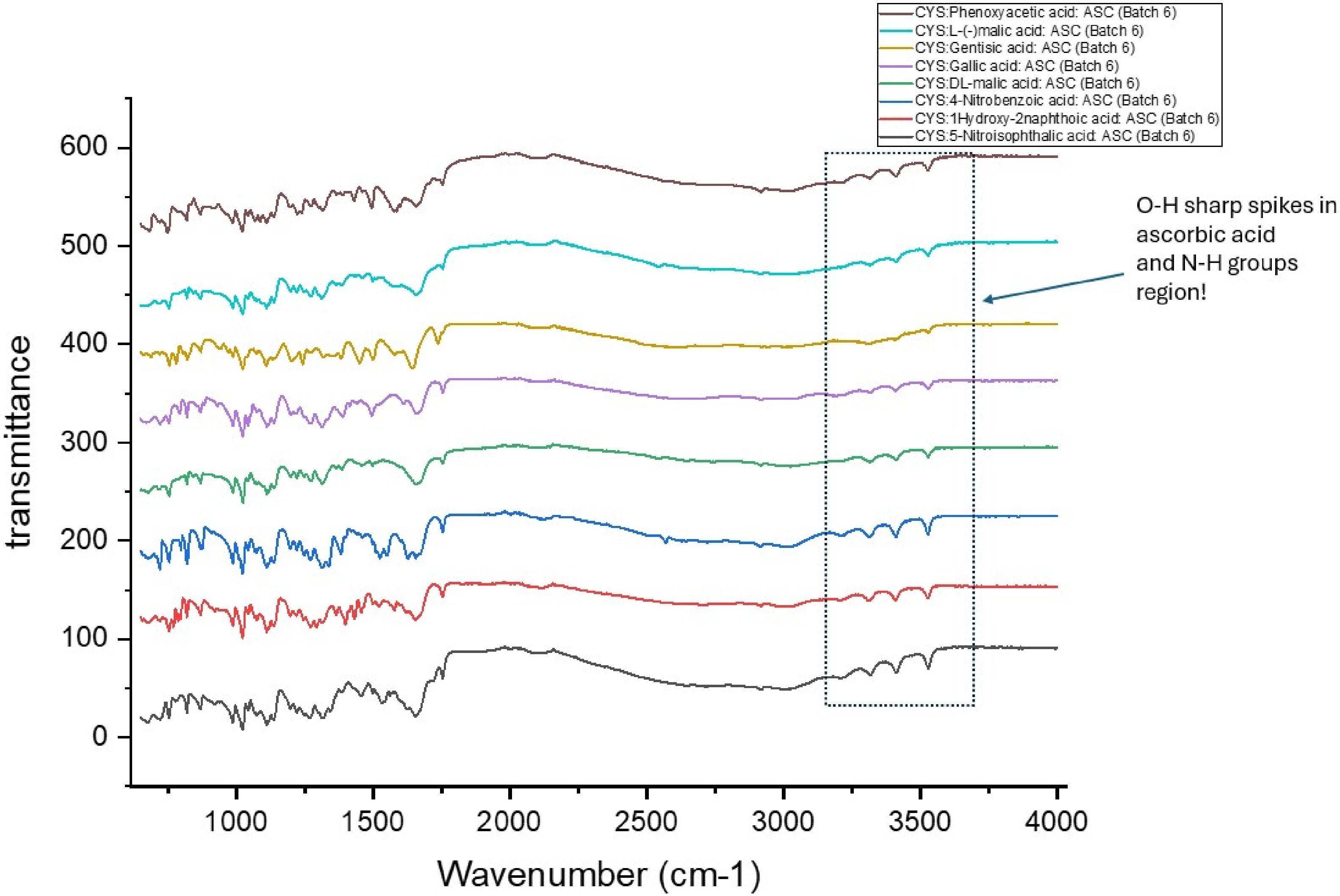


Figure S24. Stacked FTIR spectrums of the final green ball milled mixtures of batch 6. The presence of sharp spikes in the O-H group region could represent the presence of excess Ascorbic acid in the final collected samples.

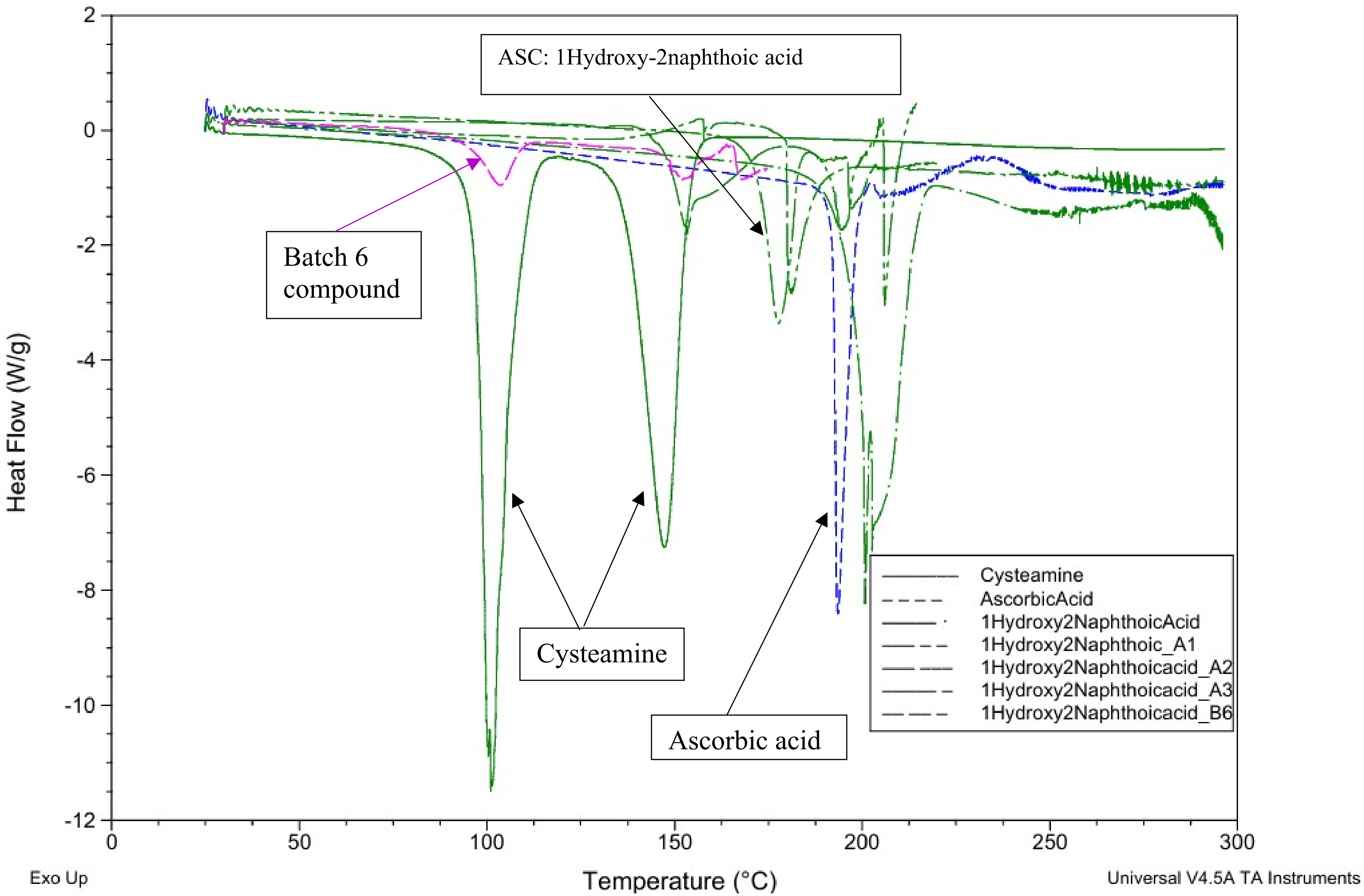


Figure S25. Stacked Differential Scanning Calorimetry (DSC) profile of CYS: 1Hydroxy-2naphthoic acid: ASC molar ratio 1: 1: 2 (batch 6). DSC thermograms of starting materials along DSC thermograms for NaCl: 1Hydroxy-2naphthoic acid: ASC 1: 1: 2 (A1), 1Hydroxy-2naphthoic acid: CYS: NaCl 1: 1: 2 (A2), and NaCl: CYS: ASC 1: 1: 2 (A3) are presented on the same graph. The final ball milled mixture demonstrates a clear distinct endothermic peak @ about 100ºC and 150ºC (coloured in pink).

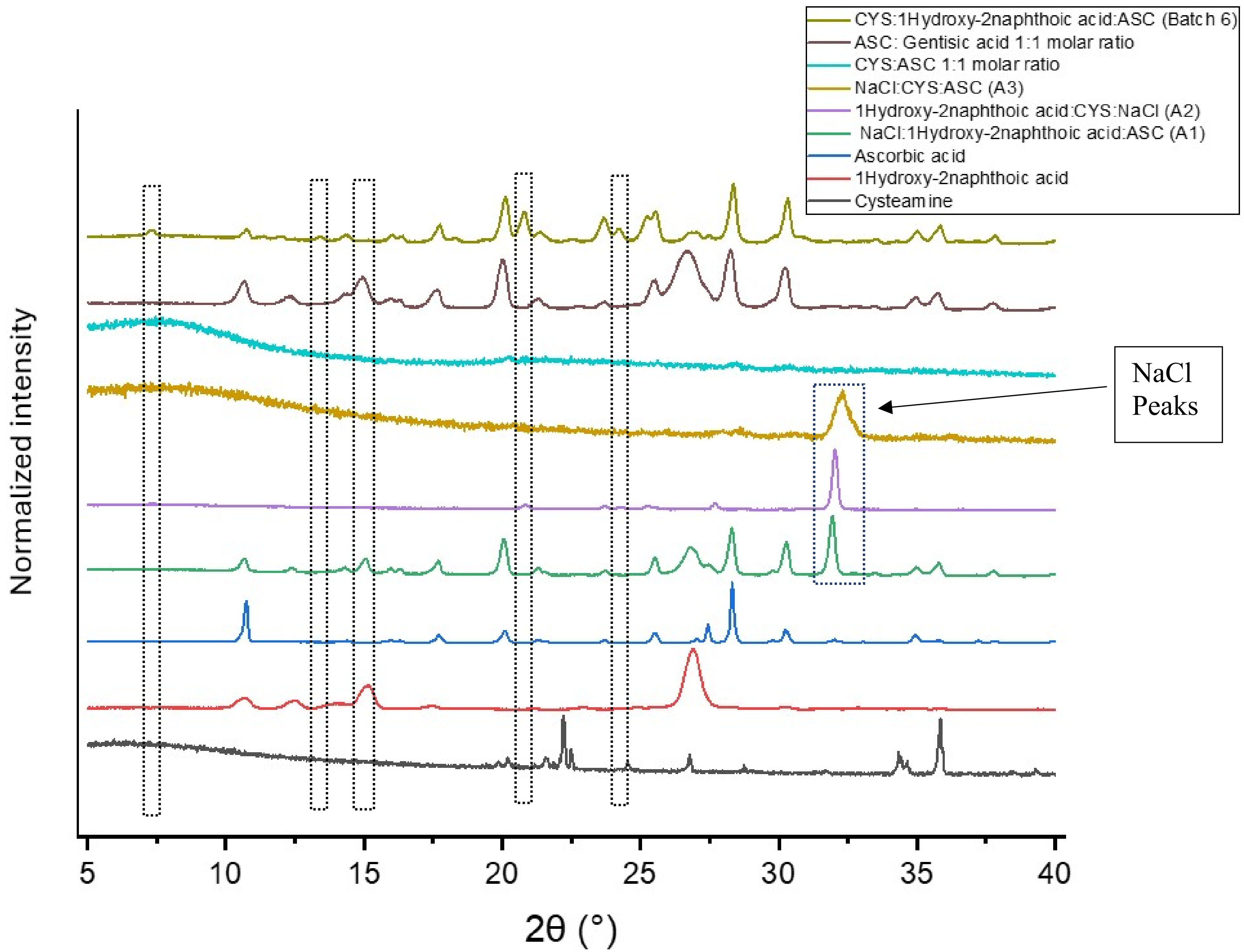


Figure S26. PXRD stack of starting materials along potential binary crystals of contents against the final green mixture of cysteamine:1Hydroxy-2naphthoic acid:ascorbic acid in the ratio of 1:1:2 molar ratio. For further clarification PXRD of cysteamine: ascorbic acid and ascorbic acid: 1Hydroxy-2naphthoic acid both in 1:1 molar ratios also added. NaCl as an inert compound used to replace each component individually (exact mass of replaced component) in exact milling conditions to batch 6 compound to facilitate comparison of potential binary crystals (A1-A3). The newly formed or omitted peaks, in the final green mixtures (batch 6) compared to the other PXRD patterns, are highlighted using dash line boxes.

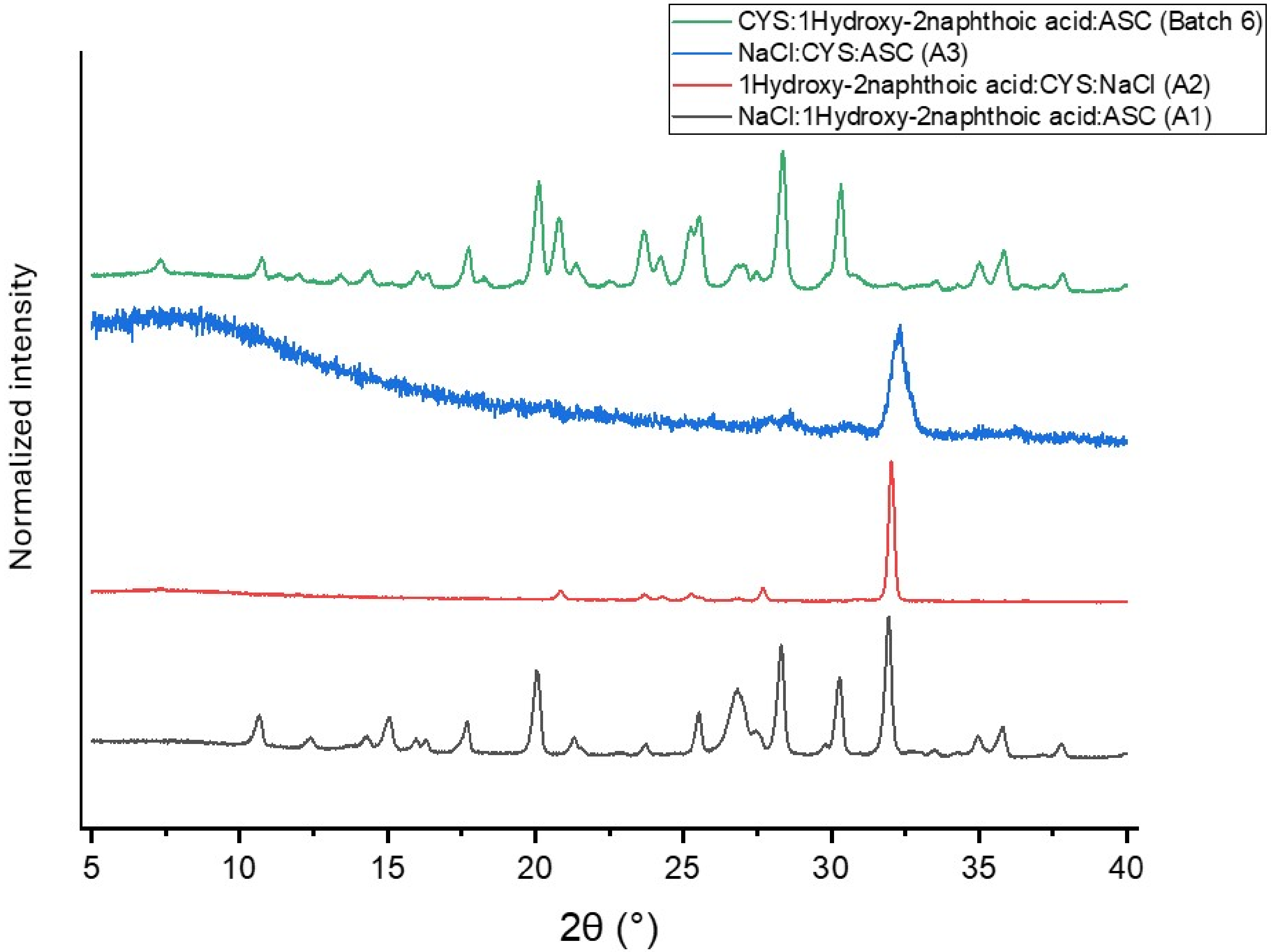


Figure S27. Magnifying the stacked PXRD patterns of A1, A2, A3 vs the final ball milled mixture of cysteamine: 1Hydroxy-2naphthoic acid: ascorbic acid 1:1:2 molar ratio (batch 6).

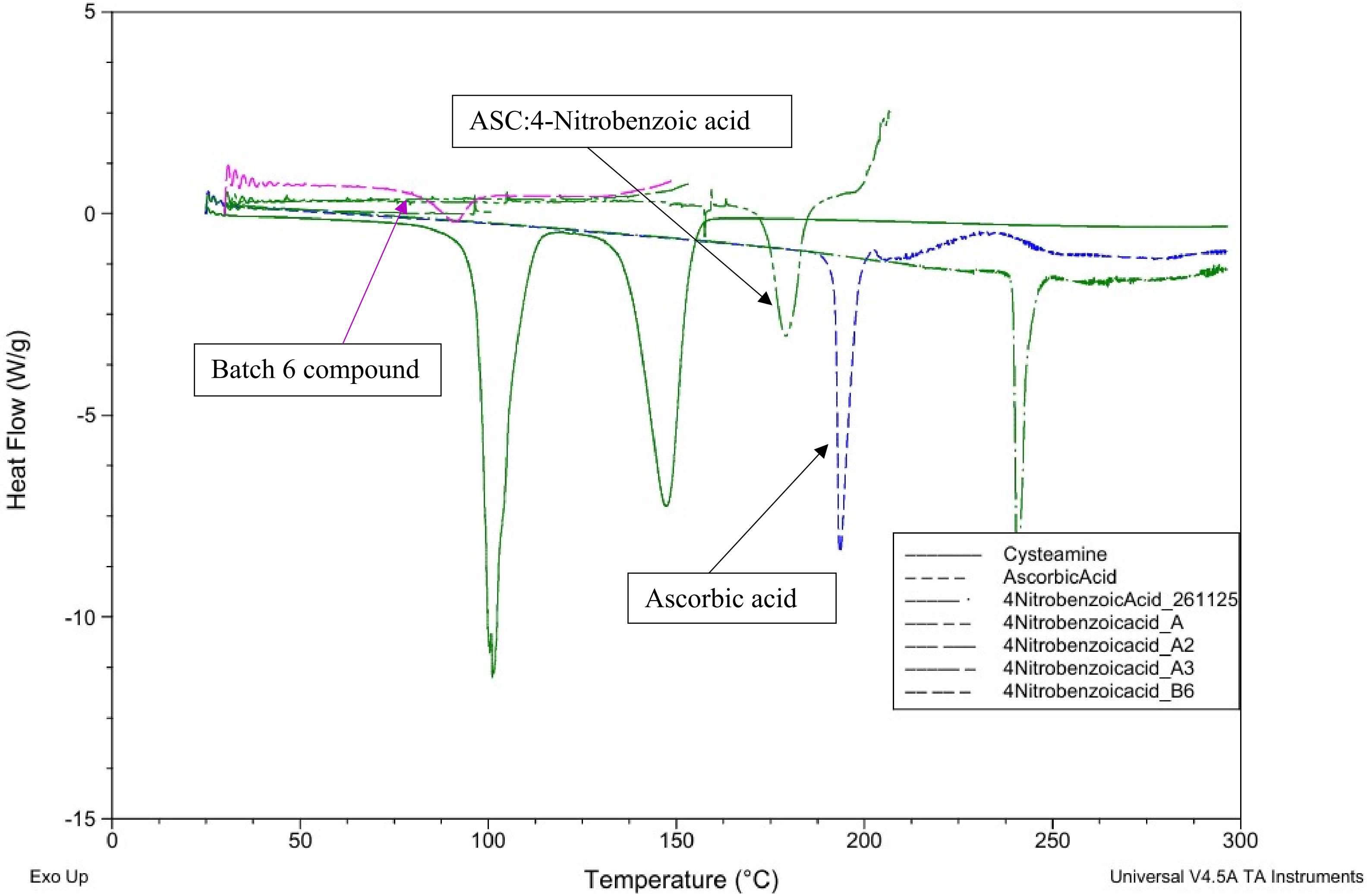


Figure S28. Stacked Differential Scanning Calorimetry (DSC) profile of CYS: 4-Nitrobenzoic acid: ASC molar ratio 1: 1: 2 (batch 6). DSC thermograms of starting materials along DSC thermograms for NaCl: 4-Nitrobenzoic acid: ASC 1: 1: 2 (A1), 4-Nitrobenzoic acid: CYS: NaCl 1: 1: 2 (A2), and NaCl: CYS: ASC 1: 1: 2 (A3) are presented on the same graph. The final ball milled mixture demonstrates a clear distinct endothermic peak @ about 90ºC (coloured in pink).

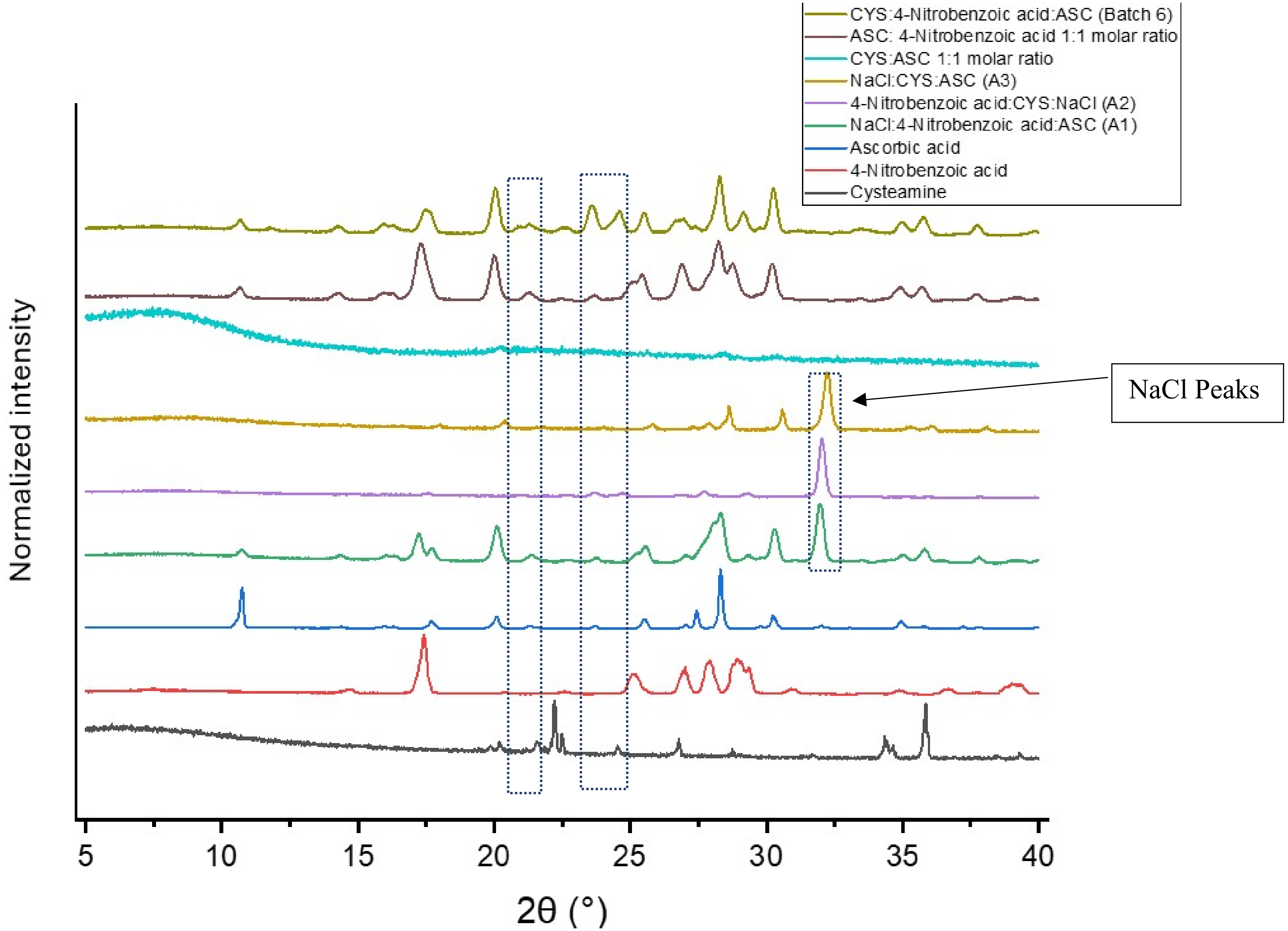


Figure S29. PXRD stack of starting materials along potential binary crystals of contents against the final green mixture of cysteamine:4-Nitrobenzoic acid: ascorbic acid in the ratio of 1:1:2 molar ratio. For further clarification PXRD of cysteamine: ascorbic acid and ascorbic acid: 4-Nitrobenzoic acid both in 1:1 molar ratios also added. NaCl as an inert compound used to replace each component individually (exact mass of replaced component) in exact milling conditions to batch 6 compound to facilitate comparison of potential binary crystals (A1-A3). The newly formed or omitted peaks, in the final green mixtures (batch 6) compared to the other PXRD patterns, are highlighted using dash line boxes.

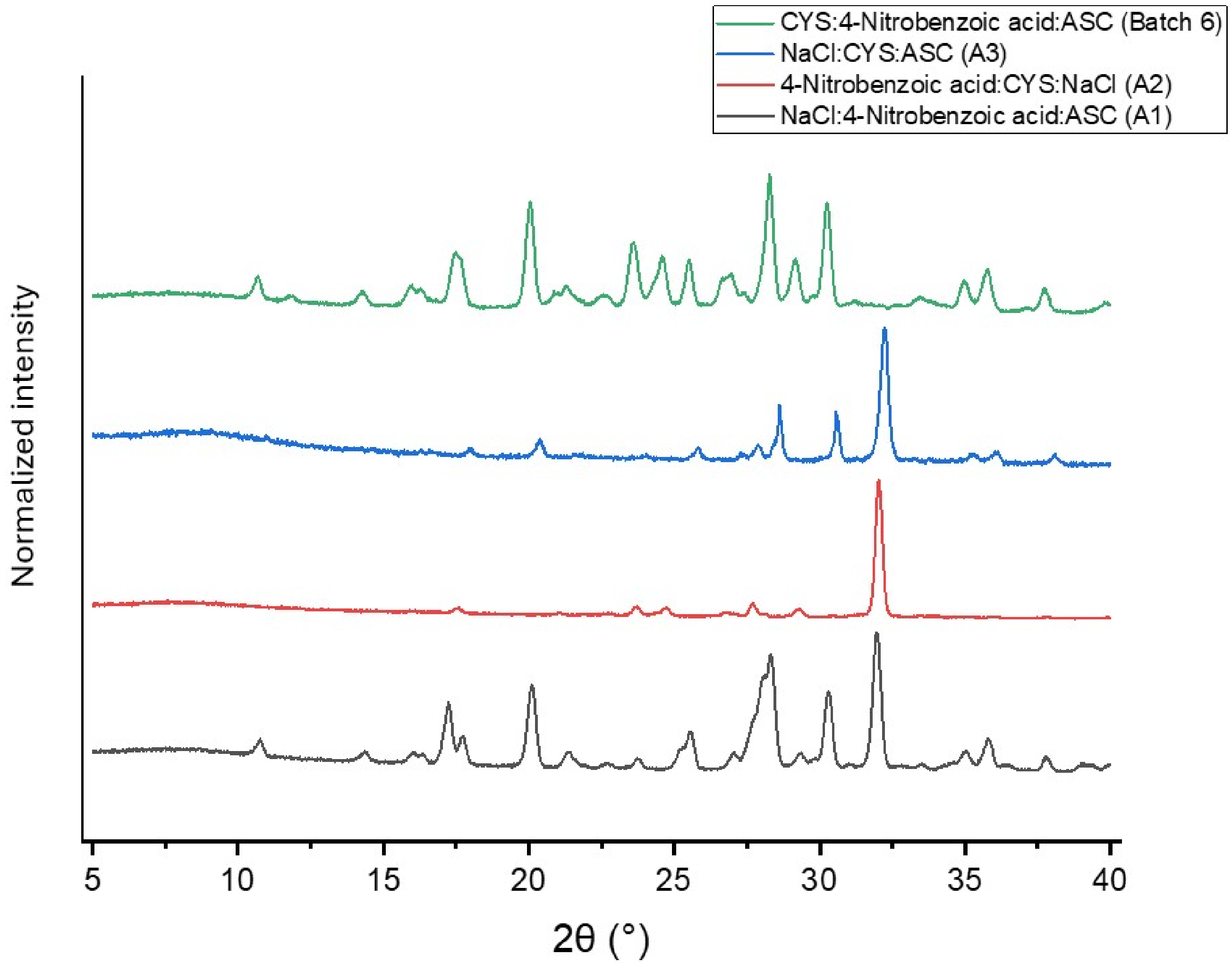


Figure S30. Magnifying the stacked PXRD patterns of A1, A2, A3 vs the final ball milled mixture of cysteamine: 4-Nitrobenzoic acid: ascorbic acid 1:1:2 molar ratio (batch 6).

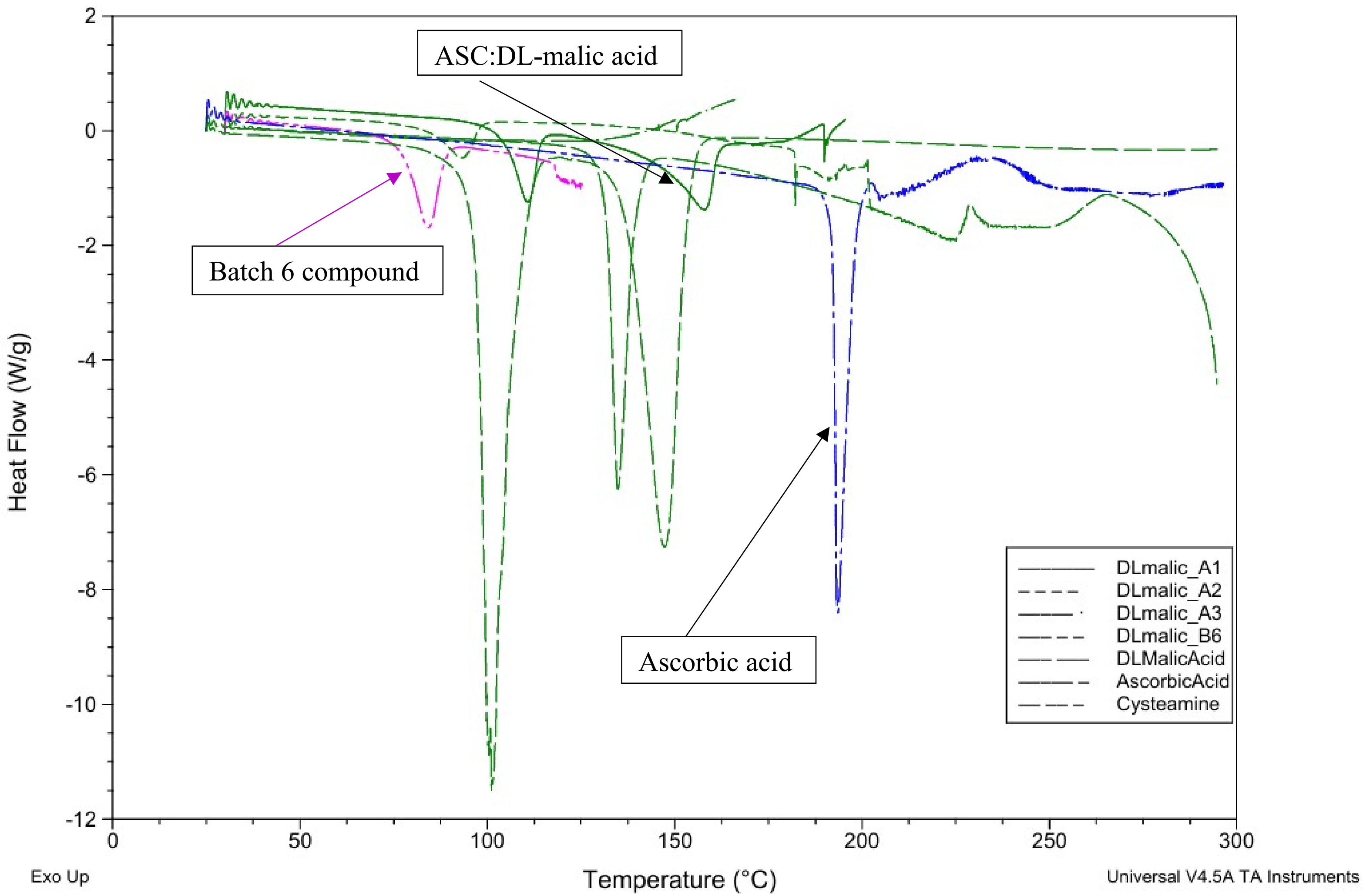


Figure S31. Stacked Differential Scanning Calorimetry (DSC) profile of CYS: DL-malic acid: ASC molar ratio 1: 1: 2 (batch 6). DSC thermograms of starting materials along DSC thermograms for NaCl: DL-malic acid: ASC 1: 1: 2 (A1), DL-malic acid: CYS: NaCl 1: 1: 2 (A2), and NaCl: CYS: ASC 1: 1: 2 (A3) are presented on the same graph. The final ball milled mixture demonstrates a clear distinct endothermic peak @ about 90ºC (coloured in pink)

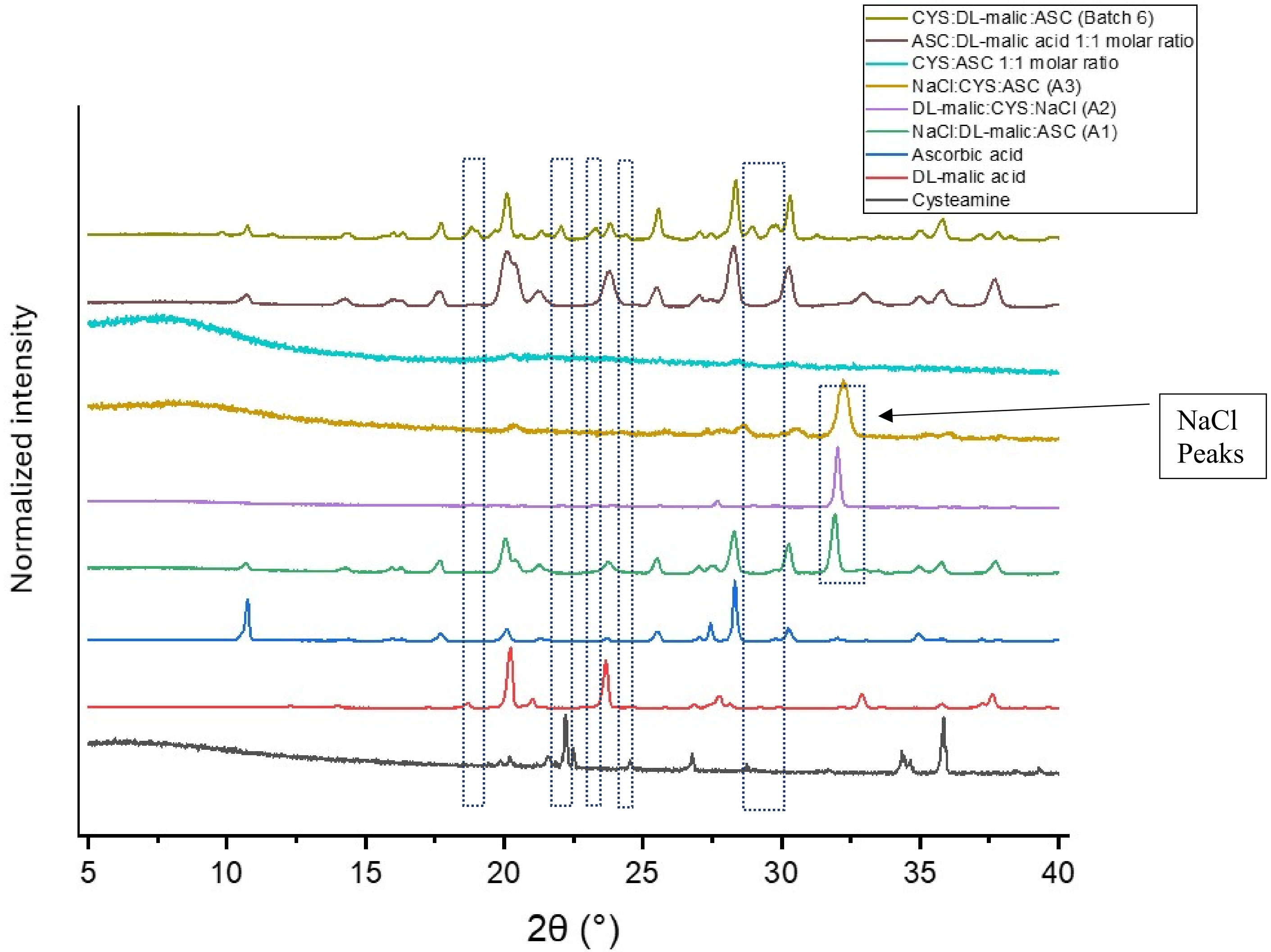


Figure S32. PXRD stack of starting materials along potential binary crystals of contents against the final green mixture of cysteamine:DL-malic acid:ascorbic acid in the ratio of 1:1:2 molar ratio. For further clarification PXRD of cysteamine: ascorbic acid and ascorbic acid: DL-malic acid both in 1:1 molar ratios also added. NaCl as an inert compound used to replace each component individually (exact mass of replaced component) in exact milling conditions to batch 6 compound to facilitate comparison of potential binary crystals (A1-A3). The newly formed or omitted peaks, in the final green mixtures (batch 6) compared to the other PXRD patterns, are highlighted using dash line boxes.

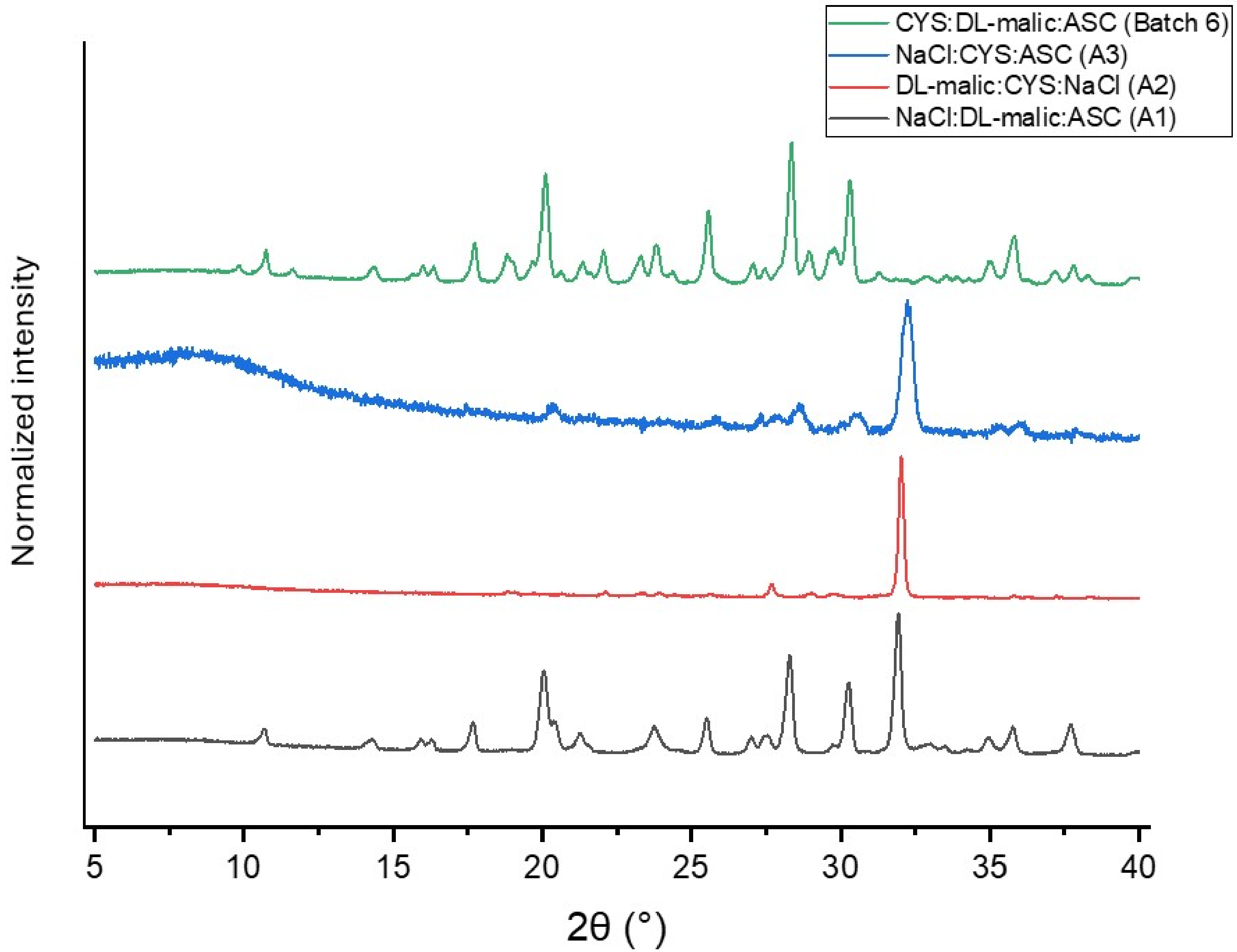


Figure S33. Magnifying the stacked PXRD patterns of A1, A2, A3 vs the final ball milled mixture of cysteamine: DL-malic acid: ascorbic acid 1:1:2 molar ratio (batch 6).

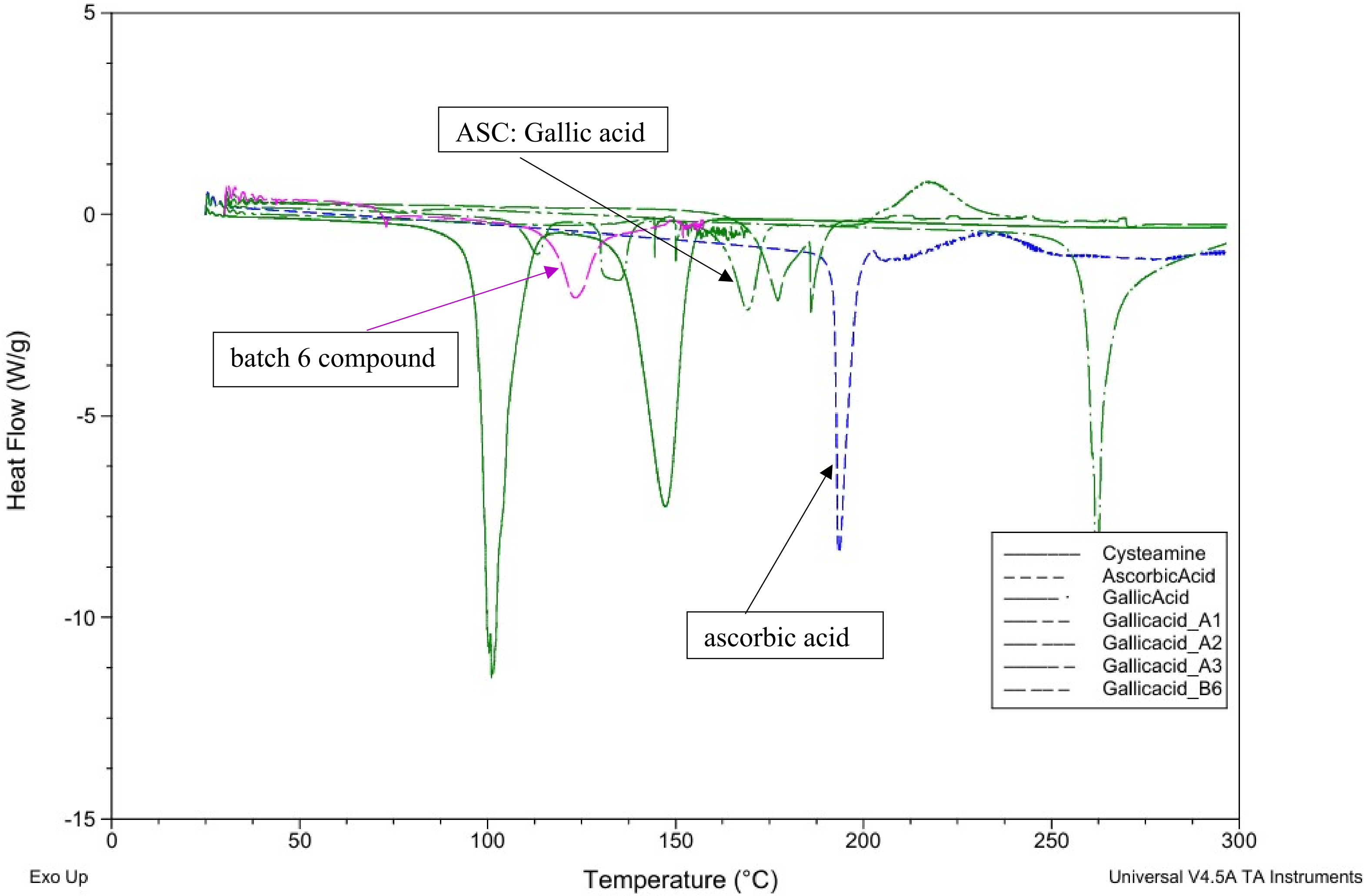


Figure S34. Stacked Differential Scanning Calorimetry (DSC) profile of CYS: Gallic acid: ASC molar ratio 1: 1: 2 (batch 6). DSC thermograms of starting materials along DSC thermograms for NaCl: Gallic acid: ASC 1: 1: 2 (A1), Gallic acid: CYS: NaCl 1: 1: 2 (A2), and NaCl: CYS: ASC 1: 1: 2 (A3) are presented on the same graph. The final ball milled mixture demonstrates a clear distinct endothermic peak @ about 130ºC (coloured in pink).

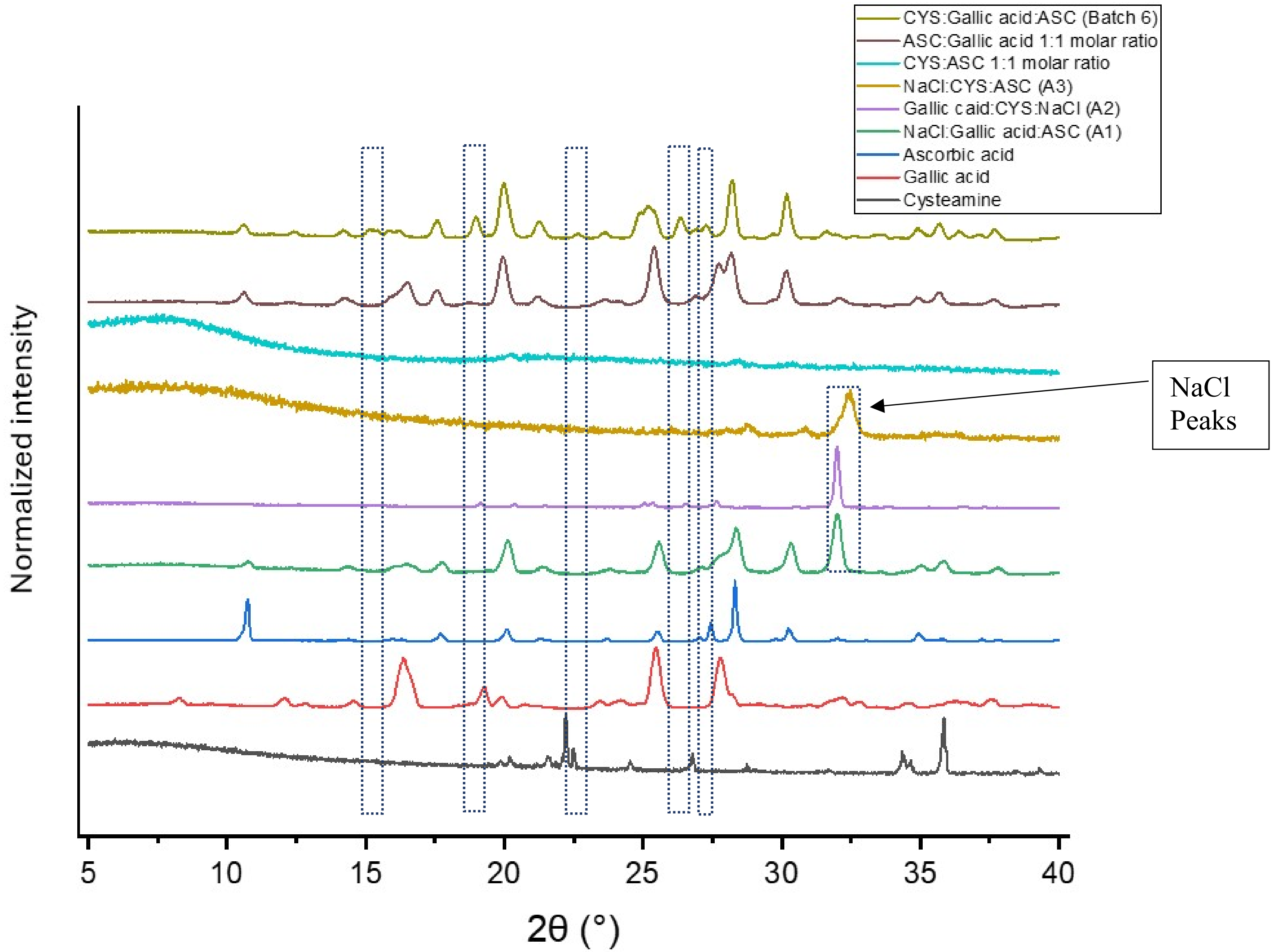


Figure S35. PXRD stack of starting materials along potential binary crystals of contents against the final green mixture of cysteamine:Gallic acid:ascorbic acid in the ratio of 1:1:2 molar ratio. For further clarification PXRD of cysteamine: ascorbic acid and ascorbic acid: Gallic acid both in 1:1 molar ratios also added. NaCl as an inert compound used to replace each component individually (exact mass of replaced component) in exact milling conditions to batch 6 compound to facilitate comparison of potential binary crystals (A1-A3). The newly formed or omitted peaks, in the final green mixtures (batch 6) compared to the other PXRD patterns, are highlighted using dash line boxes.

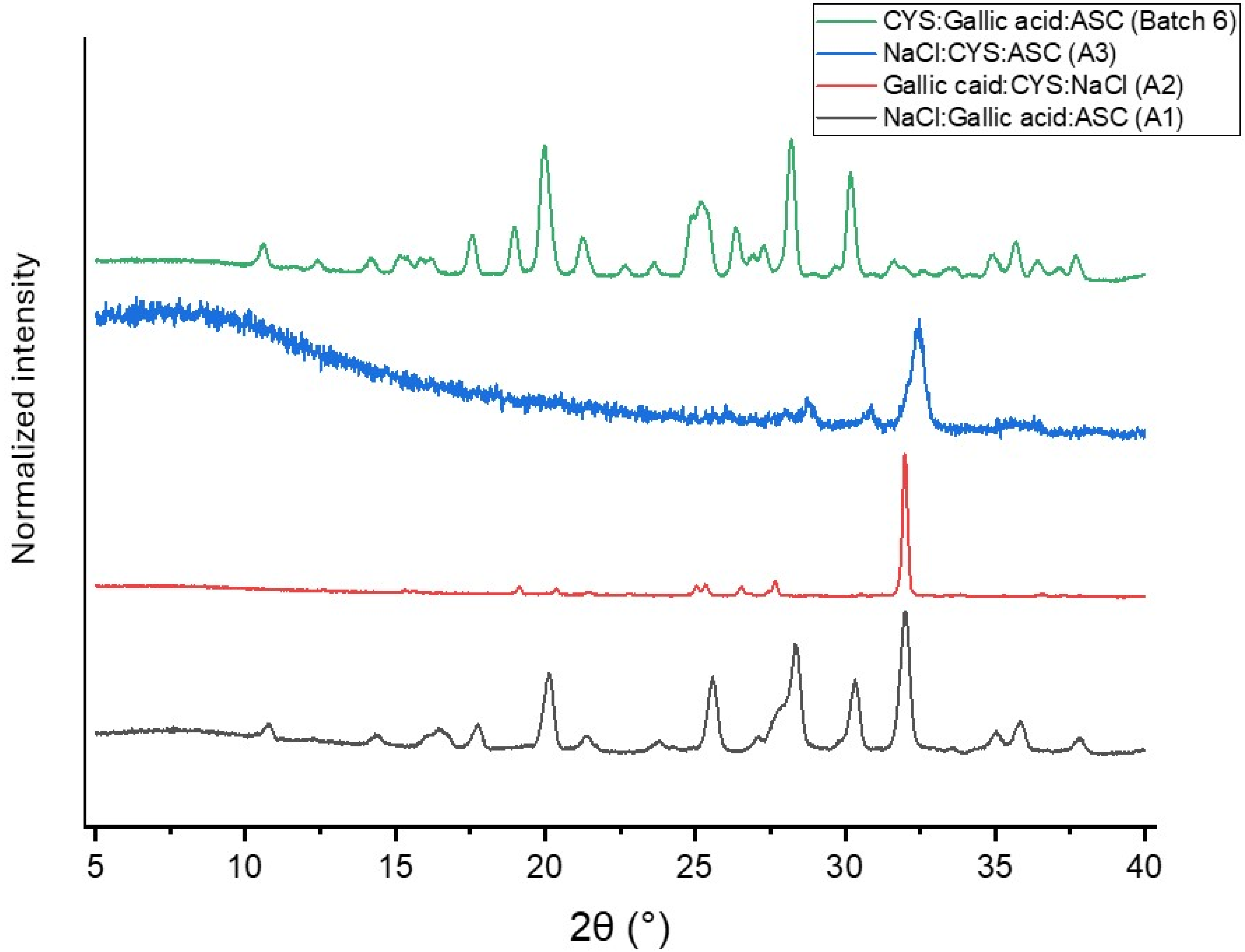


Figure S36. Magnifying the stacked PXRD patterns of A1, A2, A3 vs the final ball milled mixture of cysteamine: Gallic acid: ascorbic acid 1:1:2 molar ratio (batch 6).

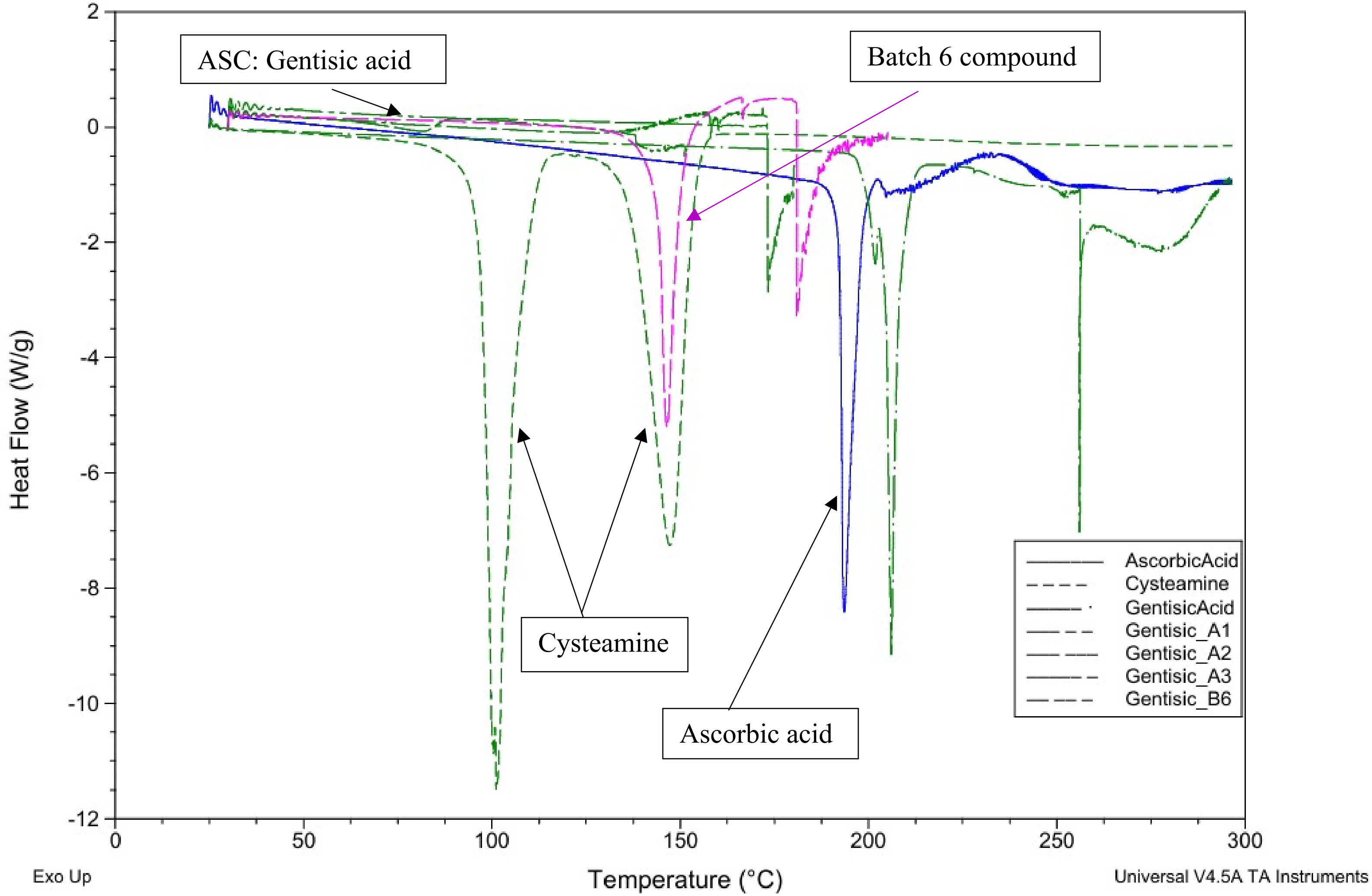


Figure S37. Stacked Differential Scanning Calorimetry (DSC) profile of CYS: Gentisic acid: ASC molar ratio 1: 1: 2 (batch 6). DSC thermograms of starting materials along DSC thermograms for NaCl: Gentisic acid: ASC 1: 1: 2 (A1), Gentisic acid: CYS: NaCl 1: 1: 2 (A2), and NaCl: CYS: ASC 1: 1: 2 (A3) are presented on the same graph. The final ball milled mixture demonstrates a clear distinct endothermic peak @ about 150ºC (coloured in pink).

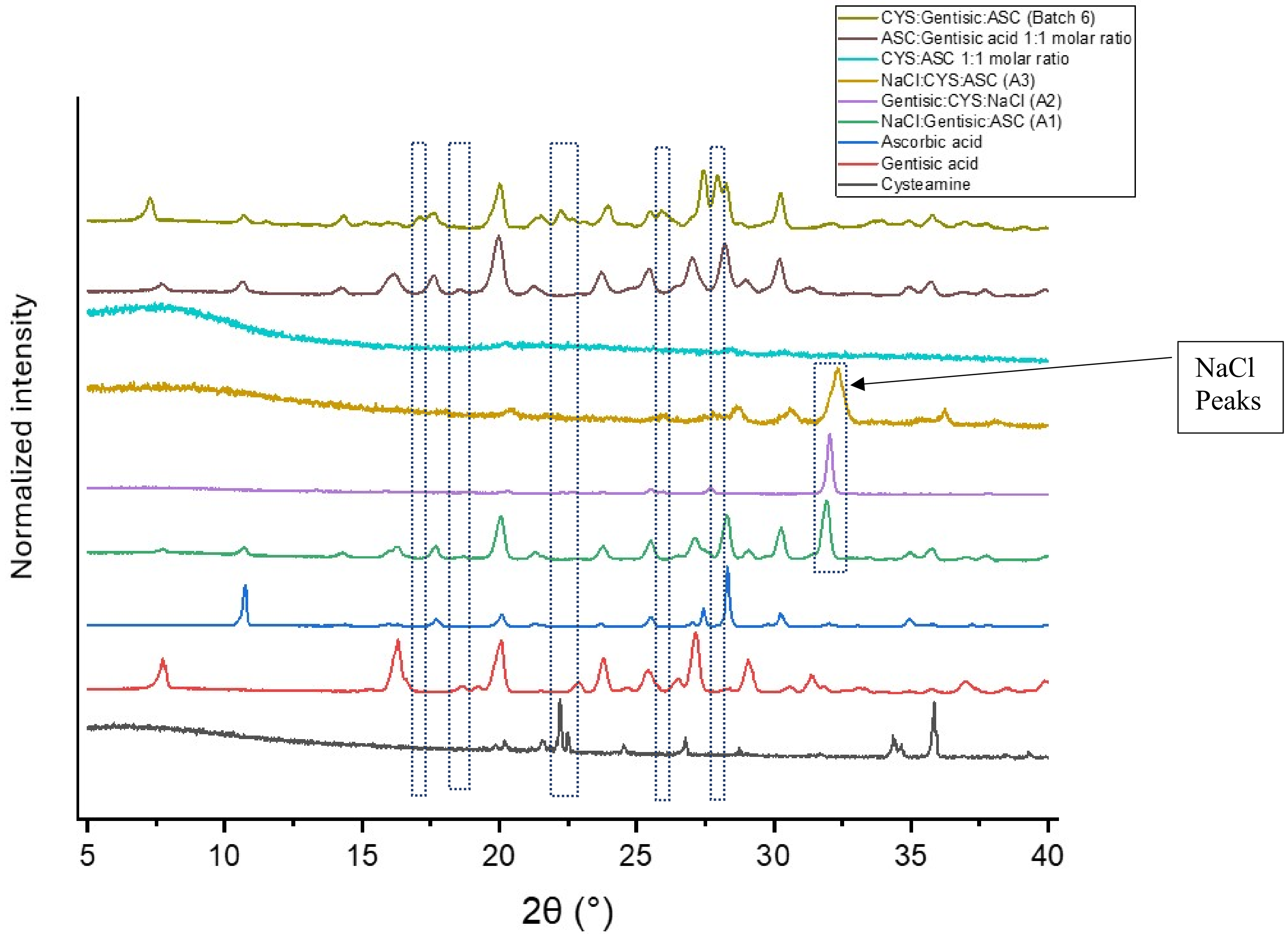


Figure S38. PXRD stack of starting materials along potential binary crystals of contents against the final green mixture of cysteamine: Gentisic acid:ascorbic acid in the ratio of 1:1:2 molar ratio. For further clarification PXRD of cysteamine: ascorbic acid and ascorbic acid: Gentisic acid both in 1:1 molar ratios also added. NaCl as an inert compound used to replace each component individually (exact mass of replaced component) in exact milling conditions to batch 6 compound to facilitate comparison of potential binary crystals (A1-A3). The newly formed or omitted peaks, in the final green mixtures (batch 6) compared to the other PXRD patterns, are highlighted using dash line boxes.

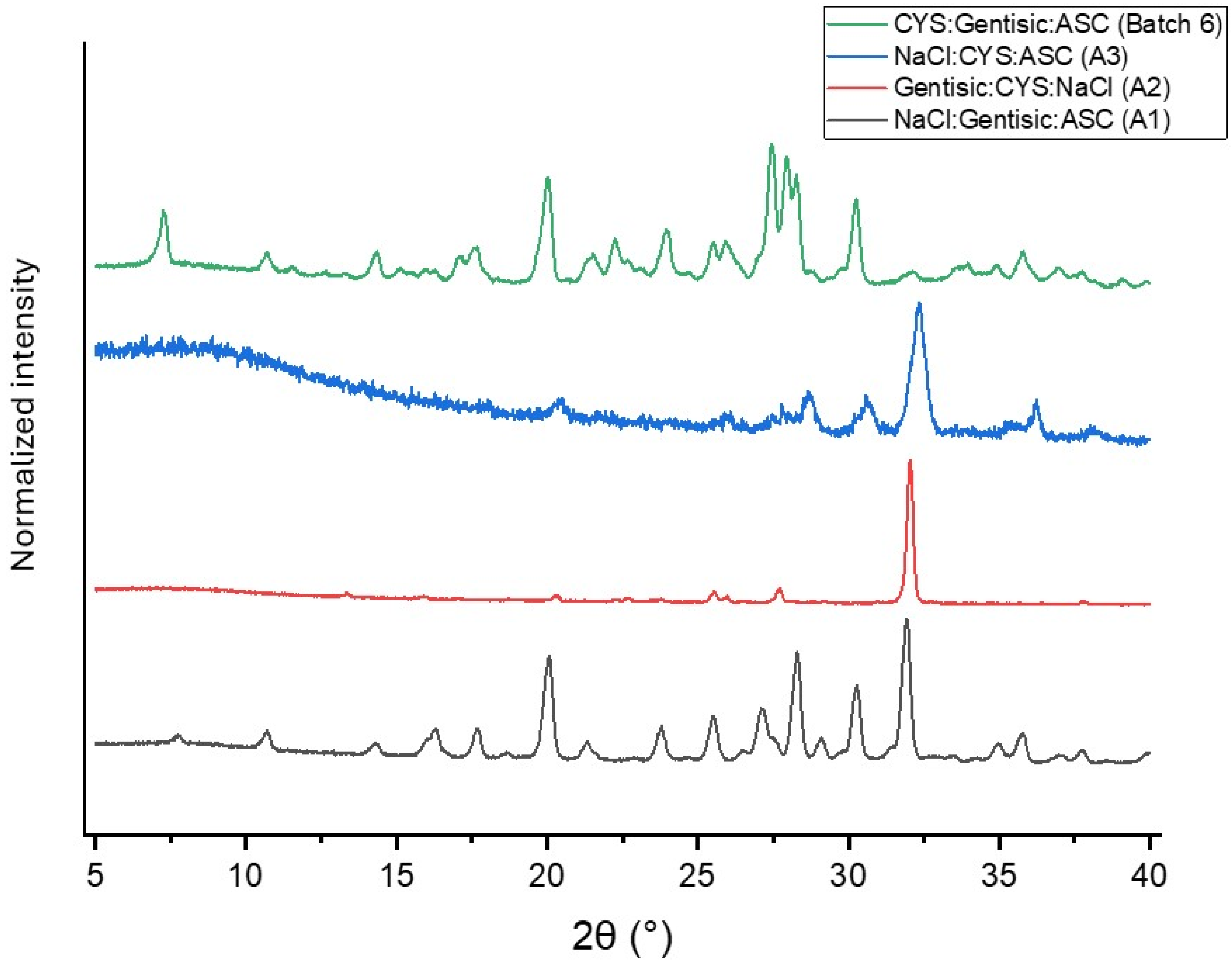

Figure S39. Magnifying the stacked PXRD patterns of A1, A2, A3 vs the final ball milled mixture of cysteamine: Gentisic acid: ascorbic acid 1:1:2 molar ratio (batch 6).

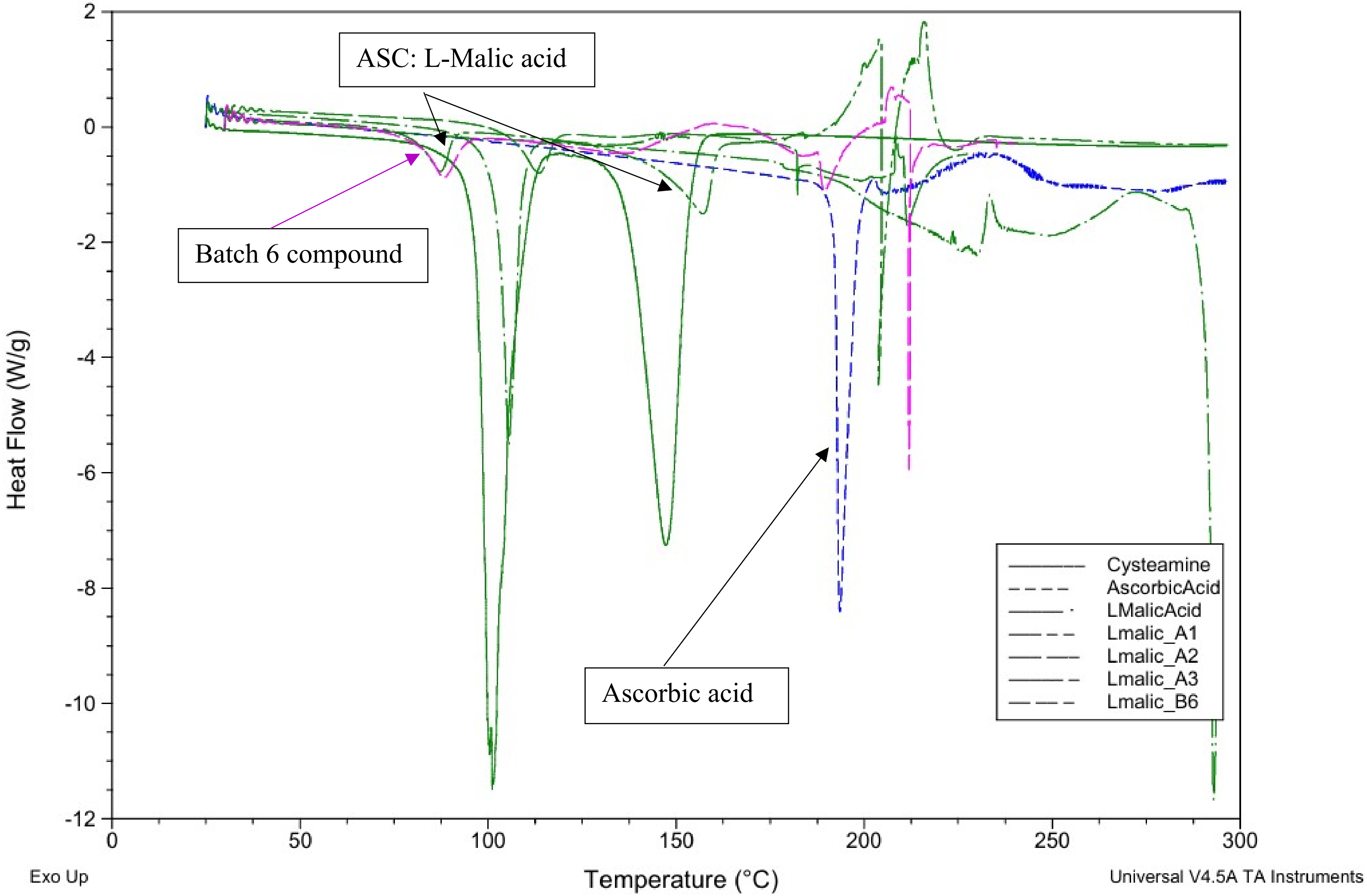


Figure S40. Stacked Differential Scanning Calorimetry (DSC) profile of CYS: L-malic acid: ASC molar ratio 1: 1: 2 (batch 6). DSC thermograms of starting materials along DSC thermograms for NaCl: L-malic acid: ASC 1: 1: 2 (A1), L-malic acid: CYS: NaCl 1: 1: 2 (A2), and NaCl: CYS: ASC 1: 1: 2 (A3) are presented on the same graph. The final ball milled mixture demonstrates a clear distinct endothermic peak @ about 90ºC (coloured in pink).

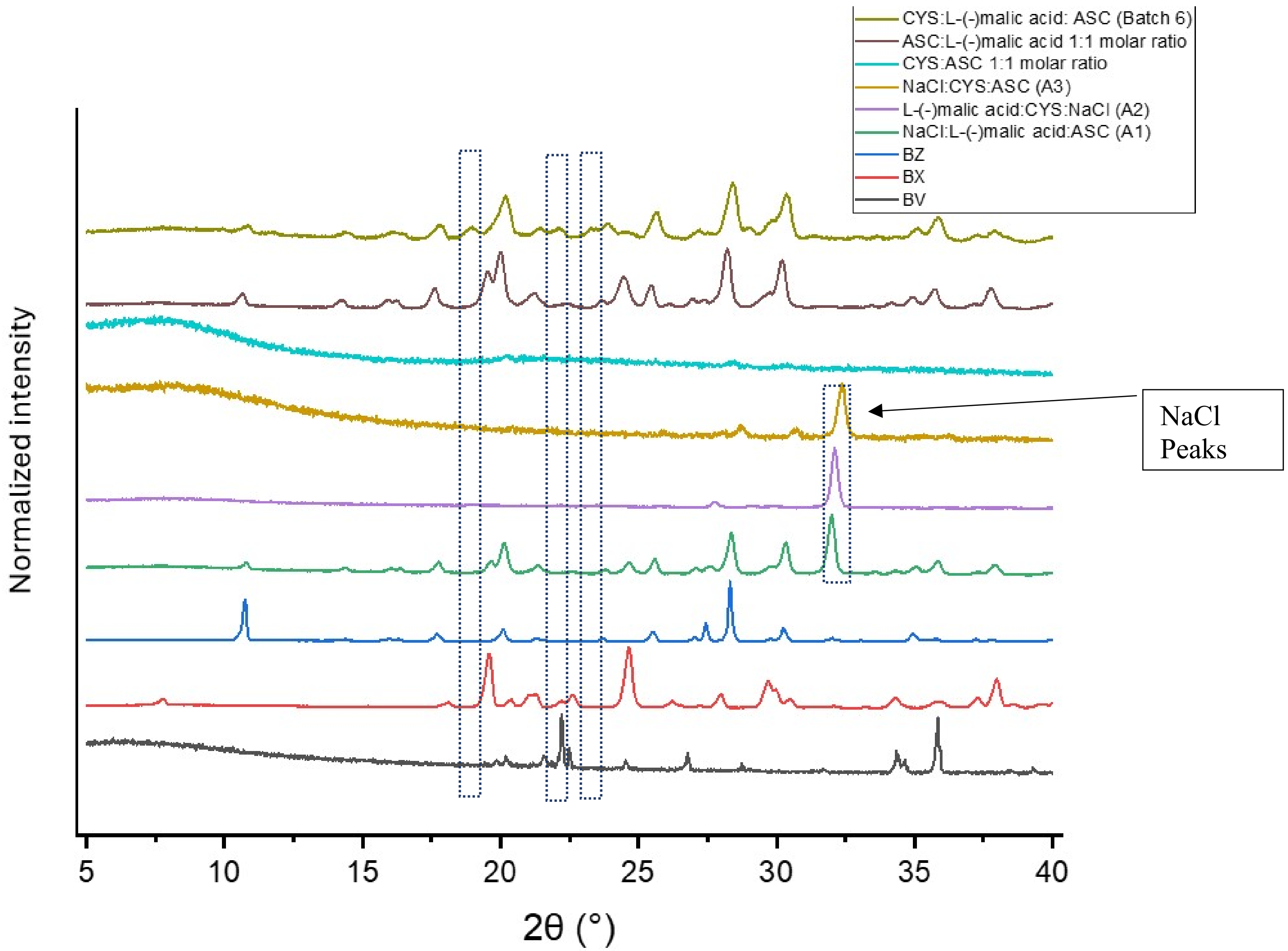


Figure S41. PXRD stack of starting materials along potential binary crystals of contents against the final green mixture of cysteamine: L-malic acid: ascorbic acid in the ratio of 1:1:2 molar ratio. For further clarification PXRD of cysteamine: ascorbic acid and ascorbic acid: L-malic acid both in 1:1 molar ratios also added. NaCl as an inert compound used to replace each component individually (exact mass of replaced component) in exact milling conditions to batch 6 compound to facilitate comparison of potential binary crystals (A1-A3). The newly formed or omitted peaks, in the final green mixtures (batch 6) compared to the other PXRD patterns, are highlighted using dash line boxes.

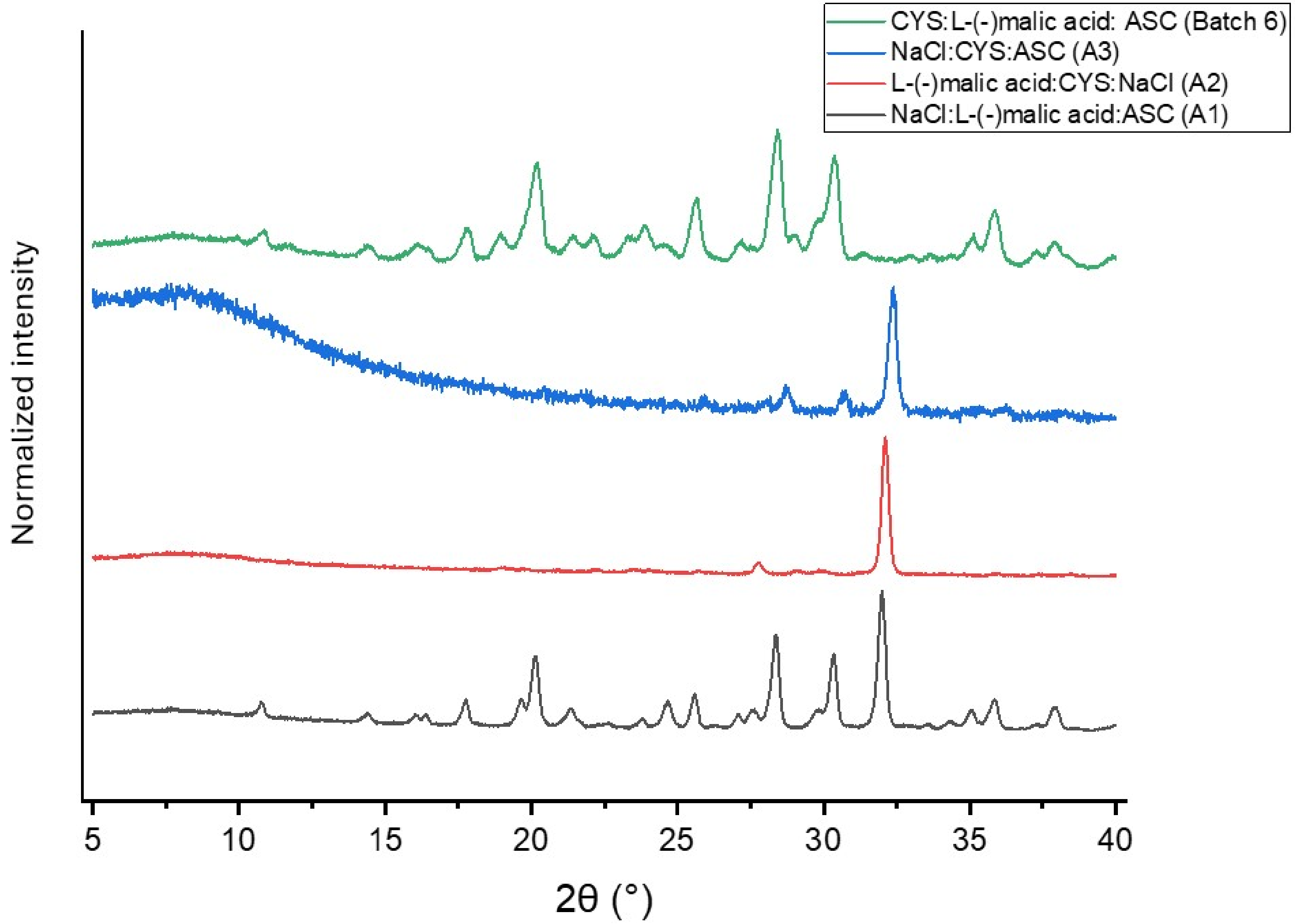


Figure S42. Magnifying the stacked PXRD patterns of A1, A2, A3 vs the final ball milled mixture of cysteamine: L-(-)malic acid: ascorbic acid 1:1:2 molar ratio (batch 6).

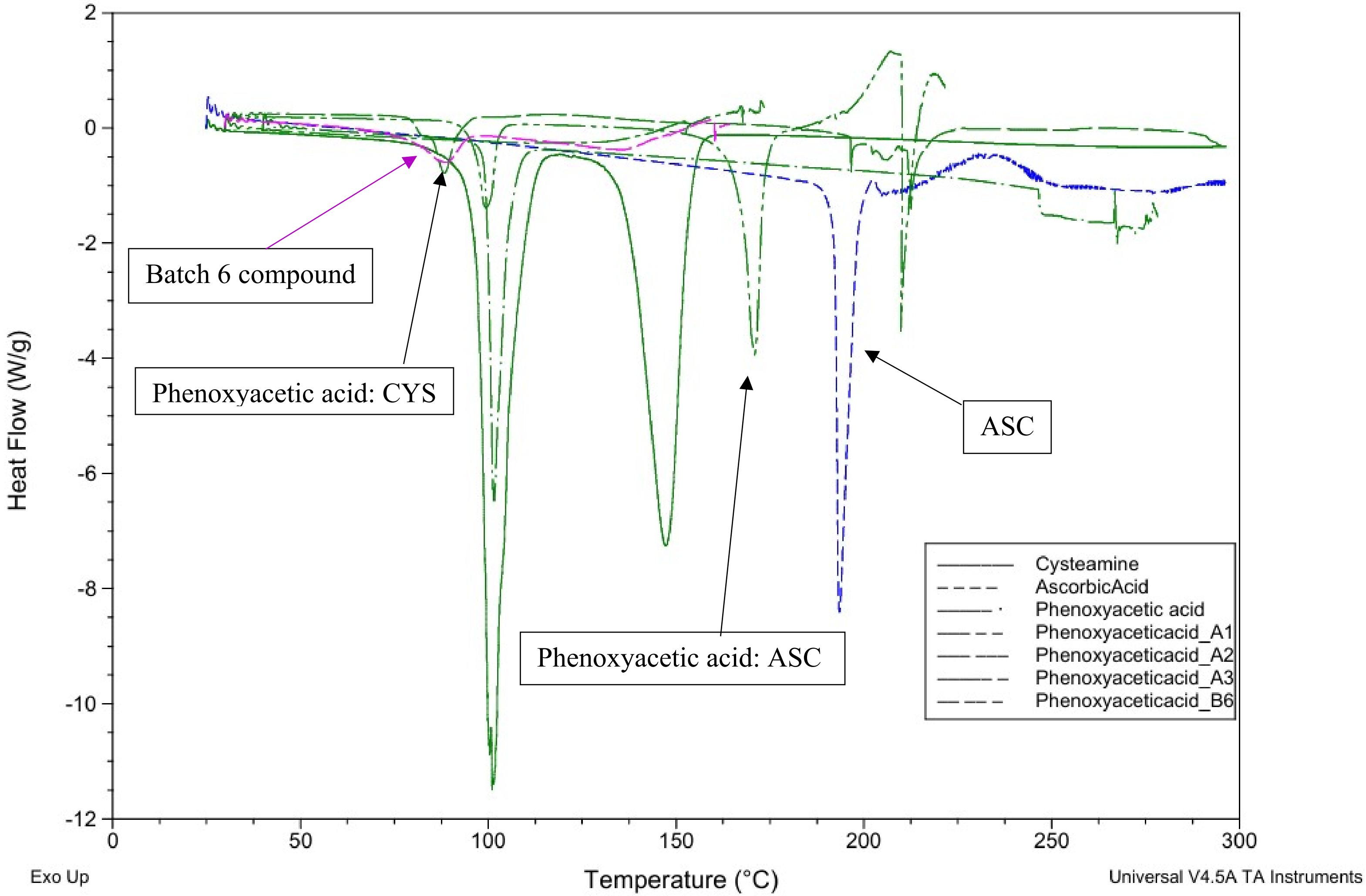


Figure S43. Stacked Differential Scanning Calorimetry (DSC) profile of CYS: Phenoxyacetic acid: ASC molar ratio 1: 1: 2 (batch 6). DSC thermograms of starting materials along DSC thermograms for NaCl: Phenoxyacetic acid: ASC 1: 1: 2 (A1), Phenoxyacetic acid: CYS: NaCl 1: 1: 2 (A2), and NaCl: CYS: ASC 1: 1: 2 (A3) are presented on the same graph. The final ball milled mixture demonstrates a clear distinct endothermic peak @ about 90ºC (coloured in pink).

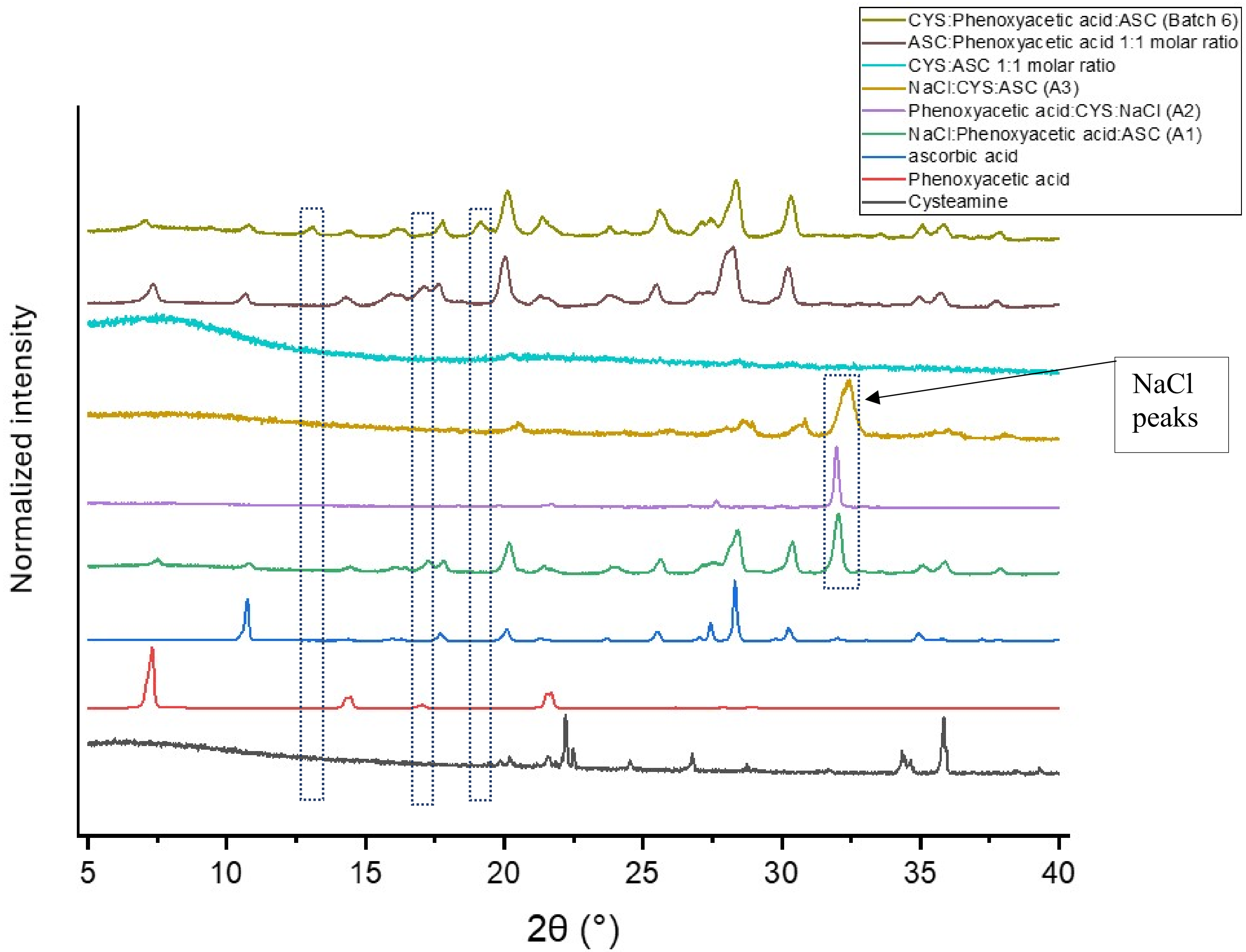


Figure S44. PXRD stack of starting materials along potential binary crystals of contents against the final green mixture of cysteamine:Phenoxyacetic acid:ascorbic acid in the ratio of 1:1:2 molar ratio. For further clarification PXRD of cysteamine: ascorbic acid and ascorbic acid: Phenoxyacetic acid both in 1:1 molar ratios also added. NaCl as an inert compound used to replace each component individually (exact mass of replaced component) in exact milling conditions to batch 6 compound to facilitate comparison of potential binary crystals (A1-A3). The newly formed or omitted peaks, in the final green mixtures (batch 6) compared to the other PXRD patterns, are highlighted using dash line boxes.

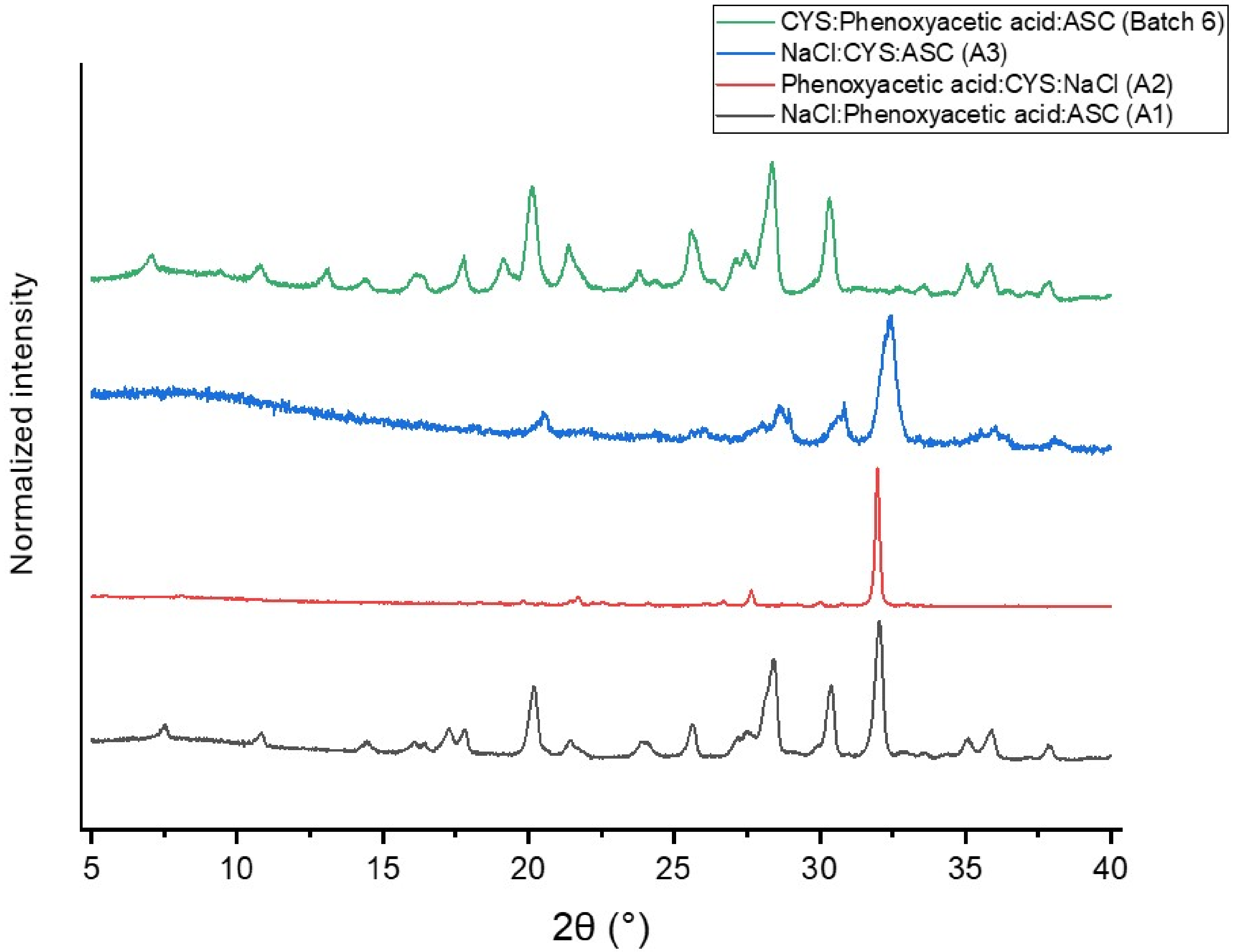


Figure S45. Magnifying the stacked PXRD patterns of A1, A2, A3 vs the final ball milled mixture of cysteamine: Phenoxyacetic acid: ascorbic acid 1:1:2 molar ratio (batch 6).

Table S8. Presents The extent of cysteamine degradation via neat ball-milling process. Milling durations of 5, 10, 15, and 30 minutes performed at 20 Hz frequency for both cysteamine and cysteamine-ascorbic acid (1:1 molar ratio) to investigate the potential anti-oxidant effect of ascorbic acid on cysteamine percentage yield in the final collected samples (n=3). The results are presented in average ± relative standard deviation. Cysteamine: ascorbic acid at 30 minutes milling could not be collected as soft and oily texture.

| Bal-milled content(s) | Milling duration (minutes) | Cysteamine percentage yield R1 | Cysteamine percentage yield R2 | Cysteamine percentage yield R3 | Average Cysteamine percentage yield ± RSD |
|---|---|---|---|---|---|
| Cysteamine | 5 | 77.54 | 80.07 | 89.29 | 82.30 ± 7.51 |
| Cysteamine | 10 | 86.25 | 78 | 81.98 | 82.08 ± 5.03 |
| Cysteamine | 15 | 80 | 83.66 | 81.41 | 81.69 ± 2.26 |
| Cysteamine | 30 | 69.58 | 81.92 | 88.11 | 79.87 ± 11.81 |
| Cysteamine: Ascorbic acid (1:1 molar ratio) | 5 | 98.67 | 99.01 | 97.88 | 98.52 ± 0.59 |
| Cysteamine: Ascorbic acid (1:1 molar ratio) | 10 | 98.43 | 98.35 | 97.37 | 98.05 ± 0.6 |
| Cysteamine: Ascorbic acid (1:1 molar ratio) | 15 | 97.60 | 98.35 | 97.42 | 97.79 ± 0.5 |
| Cysteamine: Ascorbic acid (1:1 molar ratio) | 30 | N/A | N/A | N/A | N/A |

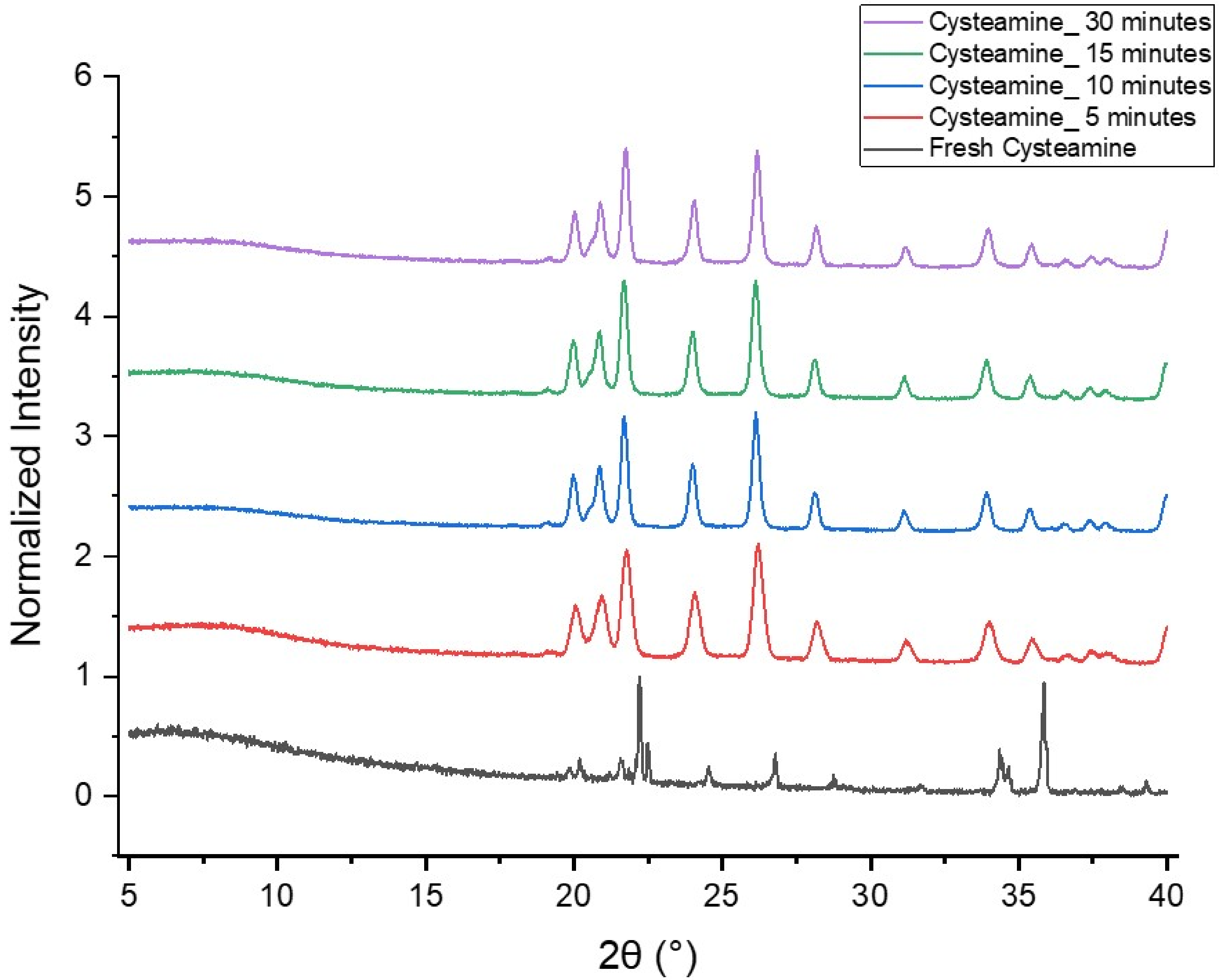


Figure S46. PXRD stack of cysteamine fresh sample vs neat ball milled cysteamine at 5, 10, 15, and 30 minutes. Frequency of 20 Hz was kept constant for all samples.

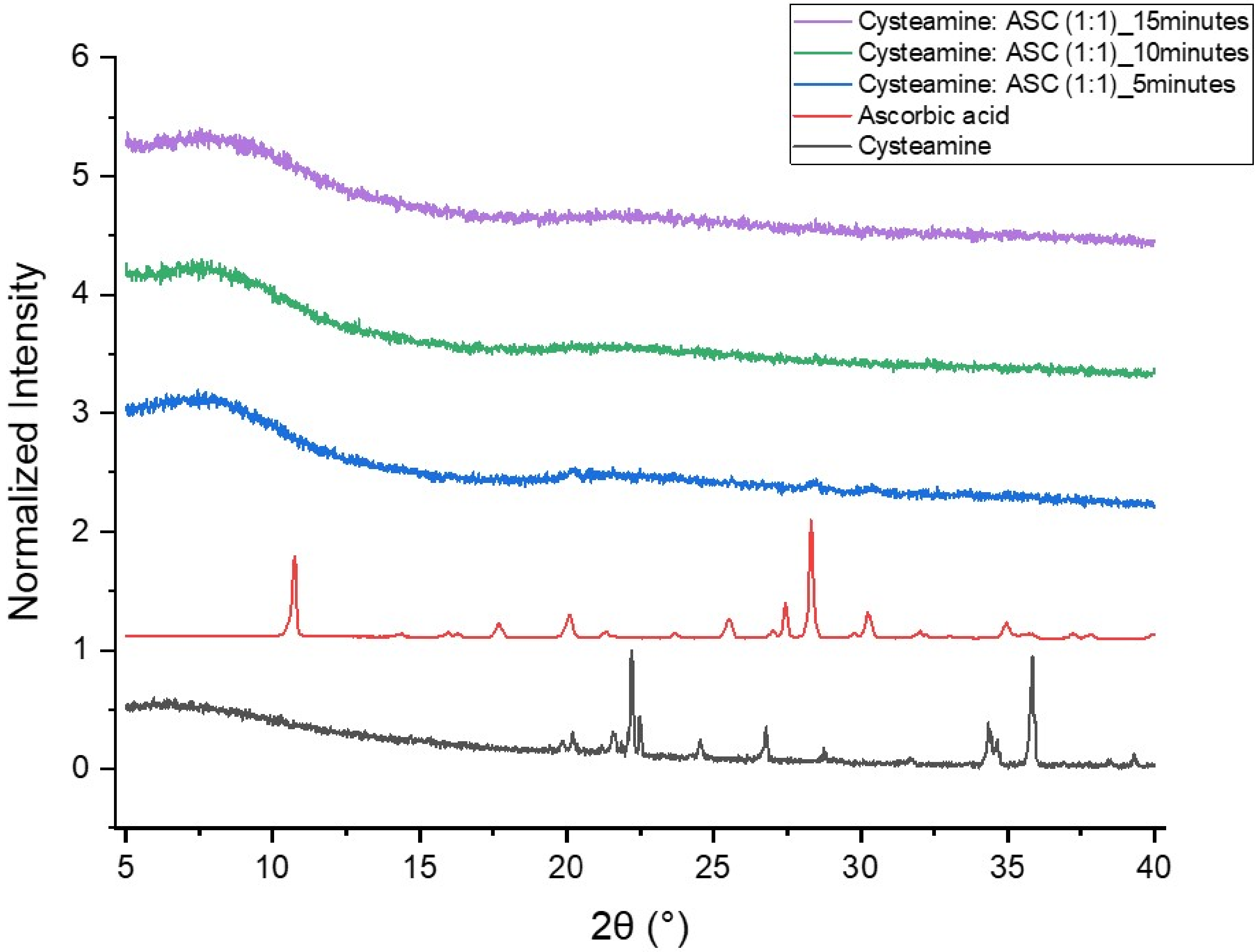


Figure S47. PXRD stack of cysteamine and ascorbic acid vs cysteamine: ascorbic acid (1:1 molar ratio) neat milling for 5, 10, and 15 minutes. Frequency of 20 Hz was kept constant for all samples.

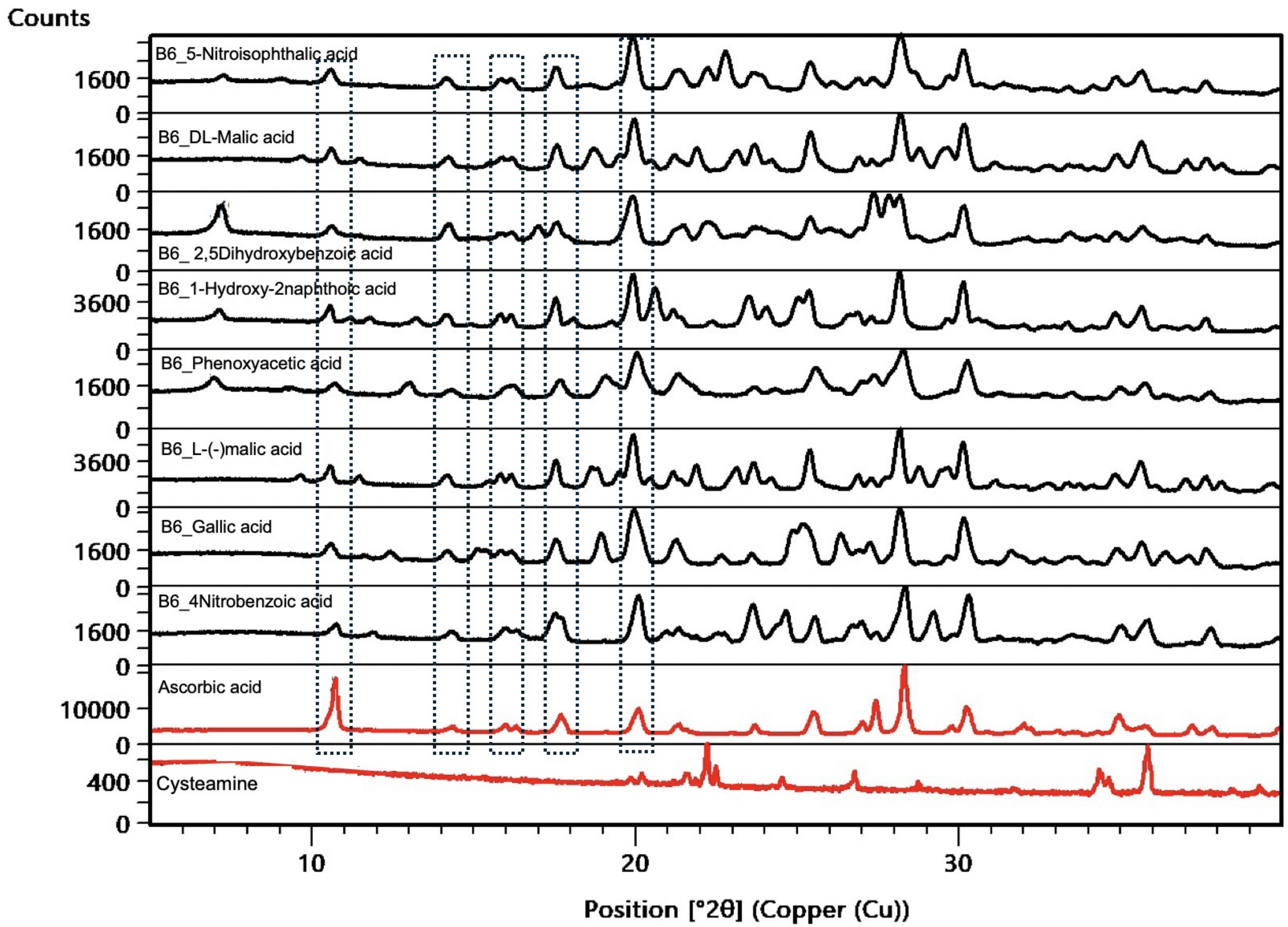


Figure S48. Presenting stack of all green mixtures of batch 6 vs cysteamine and ascorbic acid, further illustrating the presence of excess ascorbic acid, along the new crystal form, in the final mixtures.
